# Stage-specific expression of an odorant receptor underlies olfactory behavioral plasticity in *Spodoptera littoralis* larvae

**DOI:** 10.1186/s12915-021-01159-1

**Published:** 2021-10-28

**Authors:** Santosh V. Revadi, Vito Antonio Giannuzzi, Valeria Rossi, Gert Martin Hunger, Lucie Conchou, Gabriele Rondoni, Eric Conti, Peter Anderson, William B. Walker, Emmanuelle Jacquin-Joly, Fotini Koutroumpa, Paul G. Becher

**Affiliations:** 1 Department of Plant Protection Biology, Swedish University of Agricultural Sciences, Alnarp, Box 190, 23422 Lomma, Sweden; 2 INRAE, Sorbonne Université, CNRS, IRD, UPEC, Université Paris Diderot, Institute of Ecology and Environmental Sciences of Paris, Department of Sensory Ecology, Route de Saint-Cyr, 78026 Versailles Cedex, France; 3 Department of Agricultural, Food and Environmental Sciences, University of Perugia, 06121 Perugia, Italy; 4 AGRIODOR, 6 rue Pierre Joseph Colin, 35000 Rennes, France; 5 United States Department of Agriculture – Agricultural Research Service, Temperate Tree Fruit and Vegetable Research Unit, 5230 Konnowac Pass Road, Wapato, WA 98951 USA

**Keywords:** α-Humulene, *β*-Caryophyllene, CRISPR-Cas9, Electrophysiology, Larval transcriptome, Modulation, Odorant receptor, Olfactometer

## Abstract

**Background:**

The detection of environmental cues and signals via the sensory system directs behavioral choices in diverse organisms. Insect larvae rely on input from the chemosensory system, mainly olfaction, for locating food sources. In several lepidopteran species, foraging behavior and food preferences change across larval instars; however, the molecular mechanisms underlying such behavioral plasticity during larval development are not fully understood. Here, we hypothesize that expression patterns of odorant receptors (ORs) change during development, as a possible mechanism influencing instar-specific olfactory-guided behavior and food preferences.

**Results:**

We investigated the expression patterns of ORs in larvae of the cotton leafworm *Spodoptera littoralis* between the first and fourth instar and revealed that some of the ORs show instar-specific expression. We functionally characterized one OR expressed in the first instar, *SlitOR40*, as responding to the plant volatile, *β*-caryophyllene and its isomer α-humulene. In agreement with the proposed hypothesis, we showed that first but not fourth instar larvae responded behaviorally to *β*-caryophyllene and α-humulene. Moreover, knocking out this odorant receptor via CRISPR-Cas9, we confirmed that instar-specific responses towards its cognate ligands rely on the expression of SlitOR40.

**Conclusion:**

Our results provide evidence that larvae of *S. littoralis* change their peripheral olfactory system during development. Furthermore, our data demonstrate an unprecedented instar-specific behavioral plasticity mediated by an OR, and knocking out this OR disrupts larval behavioral plasticity. The ecological relevance of such behavioral plasticity for *S. littoralis* remains to be elucidated, but our results demonstrate an olfactory mechanism underlying this plasticity in foraging behavior during larval development.

**Supplementary Information:**

The online version contains supplementary material available at 10.1186/s12915-021-01159-1.

## Background

Many animals, including insects, exhibit changes in foraging behavior and food preferences during their life [1–4]. For example, in the insect model *Drosophila melanogaster*, larvae are attracted to and prefer unsaturated fatty acids over saturated fatty acids, while adults prefer saturated fatty acids [3]. In Lepidoptera, larval and adult food resources differ, and within larval instars, food requirements may also change according to the instar. For instance, the older larvae of the buckeye butterfly *Junonia coenia* (Lepidoptera: Nymphalidae) show marked preference for young plants over flowering and fruiting mature plants, contrary to the first instar larvae which are less selective [5]. In the fall armyworm *Spodoptera frugiperda* (Lepidoptera: Noctuidae), the early instar larvae feed predominantly on cotton leaves and progressively change their preferences to fruiting structures (squares and bolls) in late instars [6]. These preference shifts are attributed to the substantial increase of the larval body size, changes in digestive physiology, dietary needs across instars, plant phenology, and competition avoidance at late stages [5, 7–9]. However, the molecular mechanisms underlying behavioral plasticity during larval development are not fully understood.

The detection of relevant cues or signals via the sensory system is an initial step towards the display of behavior in insects. In larvae as in adults, the chemosensory system encompassing gustation and olfaction mediates the detection of chemicals emitted by a variety of sources, including food [10–12]. In particular, the olfactory system is of prime importance as it detects at distance food cues, such as specific plant odors for herbivorous insects [13]. It could be thus hypothesized that changes in the foraging behavior and food preference during larval development would be mediated by modifications in olfactory abilities, through changes either in sensing chemical signals in the peripheral olfactory organs or in the processing of chemical signals at higher brain centers. Changes in odor detection at the peripheral olfactory system during larval development have not been explored.

The peripheral olfactory organs of Lepidoptera larvae consist of antennae and maxillary palps [14]. Together, they house olfactory sensory neurons (OSNs) that express the chemosensory receptors responsible for olfaction: odorant receptors (ORs) and ionotropic receptors (IRs) [13]. The axons from these OSNs innervate one or several glomeruli in the antennal lobe, before the signals are projected into the higher brain center [15, 16]. Lepidoptera larvae generally have fewer sensilla and OSNs on their chemosensory organs than adults and a smaller repertoire of expressed ORs [17–21]. In vivo, ORs form a heteromeric complex with a universal co-receptor *Orco*, a prerequisite for signal transduction [22, 23]. Despite the relatively simple olfactory system of larvae, several examples show that it detects numerous host plant volatiles [21, 24, 25]. For example, Di et al. [21] showed that larvae of the cotton bollworm *Helicoverpa armigera* express only 17 ORs in the antennae and maxillary palps, but detect a wide range of odorants such as green leaf volatiles, terpenoids, and various other aromatic and aliphatic compounds.

We hypothesize that expression patterns of ORs change during development, as a possible mechanism influencing instar-specific olfactory-guided behavior and food preferences. To date, studies of OR differential expression in insects have mainly focused on the comparison between species, sexes, and physiological conditions (feeding/mating status) and comparison between larvae and adults [19, 20, 26–28]. Such studies revealed, for instance, male-biased pheromone receptors in adults and female-specific ORs related to host finding. For example, in *Bombyx mori*, females specifically express some ORs that respond to volatiles likely involved in oviposition site selection [29]. Differential expression of ORs between larval stages has not been investigated yet. To address this, we examined whether larvae of the Egyptian cotton leafworm, *Spodoptera littoralis* (Lepidoptera: Noctuidae), express different sets of ORs during development, and propose a novel mechanism of olfactory-guided behavioral modulation in insect larvae.

*Spodoptera littoralis* is a polyphagous pest across Africa and the Middle East whose larvae can feed and successfully complete their life cycle on plants belonging to more than 40 families [30]. In this species, large OR repertoires have already been described in both adults and larvae [19, 31] and odor-guided behavioral traits are well characterized [25, 32–34], providing a good basis to investigate differential expression of ORs during larval development. We selected two larval stages, first and fourth instars, to cover the developmental period where larvae actively search for food.

We first used Illumina-based RNA sequencing (RNA-seq) to compare the transcriptomes of heads of first and fourth instar larvae and revealed that some ORs were differentially expressed between the two larval stages, further confirmed by PCR analyses. We functionally characterized one of these ORs as responding to *β*-caryophyllene and α-humulene, two volatiles commonly found in *S. littoralis* host plants. In agreement with the proposed hypothesis, we showed that *β*-caryophyllene and α-humulene triggered different behavioral responses in first and fourth instar larvae. Knocking out this OR via CRISPR-Cas9, we confirmed that instar-specific responses towards its cognate ligands did rely on this OR expression. In adults, knocking out this OR impaired electrophysiological responses to *β*-caryophyllene and α-humulene, comparing the function of this receptor in vivo.

## Results

### Transcriptome sequencing and identification of odorant receptors in *S. littoralis* larvae

First, we used RNA-seq to identify ORs from the transcriptomes of first and fourth instar larval heads. A total of 15,763,159 and 30,022,191 raw sequence reads were obtained from the first and fourth instar larvae, respectively. After trimming and removing low-quality reads, we obtained 95.97% (first instar) and 94.88% (fourth instar) paired reads. Using Trinity, we assembled one transcriptome with first instar larval reads, one with fourth instars, and one with both instars + previously published adult antennae reads [31]. This last assembly yielded 50,680 sequences, with a N50 of 1954 nt, mean sequence length of 744.79 nt, and 24,264 contigs greater than 1000 nt. Benchmarking Universal Single-Copy Orthologs (BUSCO, [35]) analysis of the assembled transcriptome (first and fourth instar + adult) using the insecta_obd9 reference database showed 96.4% complete BUSCO, 75.1% complete and single copy, 21.3% complete and duplicate BUSCO, and 2.8 and 0.8% fragmented and missing BUSCOs, respectively (Additional file 1: Table S1).

Mapping first and fourth instar reads to the assembled transcriptome and estimation of expression levels revealed 34 ORs expressed in the first instar and 18 ORs in the fourth instar (Additional file 2: Table S2). Most of these ORs corresponded to previously identified receptors in *S. littoralis.* In addition, we identified one new OR, named *SlitOR70* (amino acid sequence in Additional file 3: Table S3), expressed in both first and fourth instar transcriptomes, and RT-PCR quantification confirmed the expression in both the larval stages and adult antennae (Additional file 4: Fig. S1). We followed the same OR nomenclature previously described for *S. littoralis* [19, 31].

A previous study on the same species retrieved 22 ORs expressed in the fourth instar [19]. The differences in OR numbers between our study and the already published one may have resulted from tissue collection methodology or other unknown reasons. Overall expression levels of ORs were low in our larval transcriptomes. Hence, we performed an additional exhaustive OR screening in both larval instars via RT-PCR using primer pairs designed to all the 60 previously described *S. littoralis* ORs [19, 31]. We retrieved a set of 36 ORs expressed in larvae including OR co-receptor (Additional file 2: Table S2 and Additional file 4: Fig. S1). The comparison between our transcriptome analysis and the RT-PCR analysis revealed several false positives (first instar = 16.39 %; fourth instar = 8.19 %) (Additional file 2: Table S2, values indicated with hashtag) and false negatives (first instar = 21.3 %; fourth instar = 34.42 %) (Additional file 2: Table S2, values indicated with asterisk) of OR expressions in our larval transcriptomes.

**Fig. 1:**
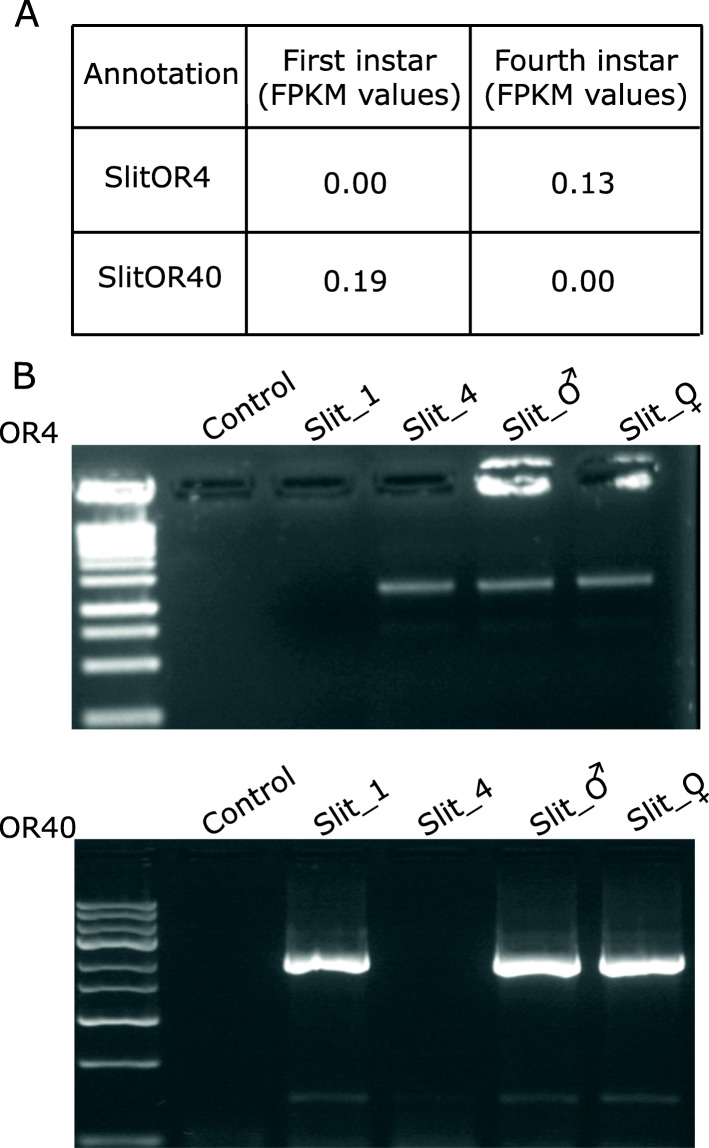
Expression of *Slit*OR4 and *Slit*OR40 in *Spodoptera littoralis* larvae: **A** Differential expression levels of two ORs in first and fourth instars measured using fragment reads per kb per million criteria. **B** Expression profile of *Slit*OR4 and *Slit*OR40 in RT-PCR assays performed using specific primer pairs, and comparison included negative control (control) and cDNAs from different tissues: first and fourth instar larval heads, and male (*Slit*_♂) and female antennae (*Slit*_♀) with 1kb gene ruler ladder (Thermo Fischer Scientific)

RT-PCR revealed that both stages expressed a large overlapping set of ORs, but evidenced that two receptors were stage-specific: *SlitOR40* was expressed only in the first instar, while *SlitOR4* was expressed only in the fourth instar, which was in agreement with the transcriptome data (Fig. 1 and Additional file 2: Table S2).

### RT-PCR and RT-qPCR assays on *SlitOR40* expression

We focused our investigation on *SlitOR40* since *SlitOR4* function was already elucidated [36]. *SlitOR40* expression, as well as that of *SlitOrco* as a positive control, was followed in detail from first to fourth larval instars by RT-PCR (Additional file 5: Fig. S2A). Fifth and sixth larval stages were not assayed in our RT-PCR analysis, and the *SlitOR*40 expression in these stages remains unknown. Whereas *SlitOrco* was detected as expected in all samples, *SlitOR40* expression was detected in first, second, and third instars, as well as in adult antennae, but it was not detected in fourth instar larval tissue (Additional file 5: Fig. S2A).

To further quantify the expression levels of *SlitOR40*, we performed RT-qPCR. First, *Orco* expression was quantified in first and fourth larval instar heads and adult male antennae using reference genes *β*-actin, L13a, and Ef1a [37]. Since *Orco* forms a heteromeric complex with ORs in nearly all OSNs [22, 38], *Orco* expression in the chemosensory tissue reflects the overall expression of ORs. We found that the relative *Orco* expression in the male antennae was significantly higher compared to first instar (187.09-fold) and fourth instar (128.38-fold) larval head tissues (*Df* = 28, *t* = −22.622; *P* < 0.001; *Df* = 28, *t* = 50.441, *P* < 0.001, respectively) (Additional file 5: Fig. S2B), probably because OSNs are enriched in adult antennae tissues compared to whole heads of larvae. Moreover, in the fourth instar larvae, *Orco* expression was 2.11-fold higher compared to first instar (*t* = 9.33; *Df* = 28; *P* < 0.001). As *Orco* level should correlate with the number of OSNs, we further used *SlitOrco* as a reference gene for *SlitOR40* quantification to ensure tissue normalization according to OSN numbers. Doing this, we revealed that *SlitOR40* relative expression in first instar larvae was significantly higher (19.97-fold) compared to adult male antennae (*t* = 26.532; *Df* = 28; *P* < 0.001), while there was no expression detected (*t* = 27.693, *Df* = 28; *P* < 0.001) in the fourth larval instar (Additional file 5: Fig. S2C), confirming RNA-seq and RT-PCR observations.

### *SlitOR*40 functional study

**Fig. 2:**
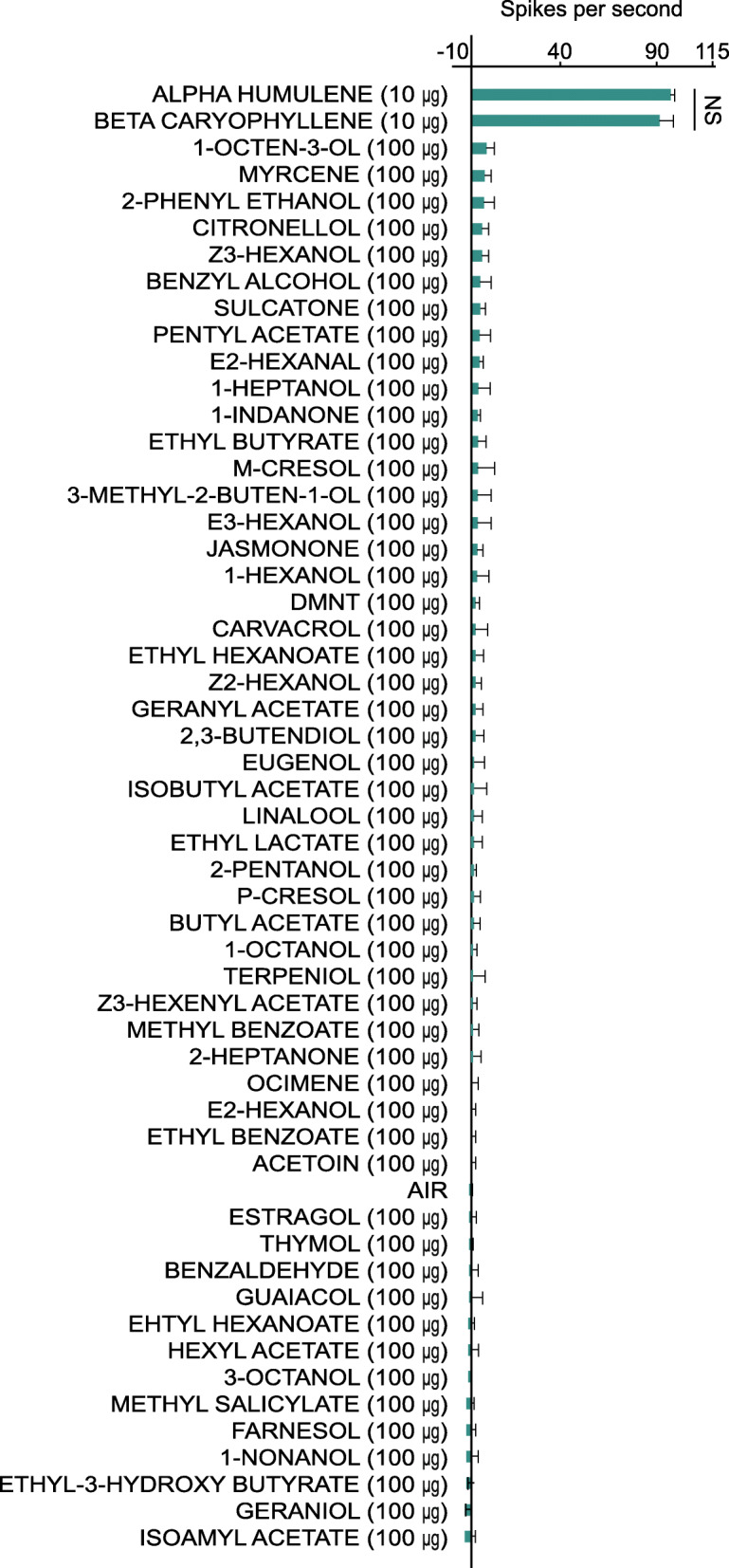
Single sensillum recordings from neurons expressing *Spodoptera* littoralis SlitOR40 in *Drosophila melanogaster* antennae. Values correspond to spikes per second produced during odor stimulation. The odor panel consisted of 54 compounds, individually loaded in disposable Pasteur glass pipettes at a dose of 100 μg. Differences in the electrophysiological responses from the empty neuron flies to *β*-caryophyllene (10 μg) and α-humulene (10 μg) were tested using the Student *t*-test. None of the remaining compounds in the panel responded significantly. The compounds are listed in decreasing order of signal strength. Error bars indicate standard deviations. The analyses were based on recordings from six flies; *P* = 0.76. NS not significant

As *SlitOR40* presented a biased expression to first instars, we assayed for its ligands as they may represent important cues for this stage. To identify these ligands, *SlitOR40* was heterologously expressed in the *Drosophila* empty neuron system [39]. The transformed *Drosophila* neurons were stimulated with a panel of 54 odorants and responses were measured via single sensillum recordings. At a 10-μg dose of stimuli, *SlitOR40* showed significant responses to *β*-caryophyllene and its isomer α-humulene (Fig. 2), two plant volatiles commonly found in *S. littoralis* host plants [11, 40, 41]. *SlitOR40* did not show a significant response to any other chemical in the panel tested even at higher doses of 100 μg.

### Generation and characterization of *SlitOR*40-knockout lines

In order to test the behavioral importance of *SlitOR40*, we used CRISPR-Cas9 to knock out this gene in the *S. littoralis* genome. We tested two single guide RNAs (sgRNA) designed to target the predicted first and the second exons of the *SlitOR40* gene (Additional file 6: Fig. S3A). Injection of the Cas9-sgRNA complex in eggs resulted in the generation of three different mutations in the gene, all leading to a premature termination codon (Additional file 6: Fig. S3B and S3C). One of the mutations resulted in a deletion (−) of 445 base pairs (bp) (line-10), while two other mutations resulted in an insertion (+) and a deletion (−) (line-38 = − 440 bp and + 20 bp; line-2 = − 446 bp and + 5 bp). The line-10 was chosen for crossing to homozygotes and subsequent phenotyping using behavioral and electrophysiological assays.

### Larval behavioral responses to *β*-caryophyllene and α-humulene

To investigate if larval behavioral response to the SlitOR40 ligands would differ between larval stages and according to expression (or not) of SlitOR40, we conducted behavioral assays in a two arm olfactometer (Y-tube). We first ran a positive control looking at the behavior of first and fourth instar larvae of wild type (WT) and *SlitOR40*-knockout (KO) genotypes to one of the preferred host plants, cotton [42], which was provided as leaf discs (Additional file 7: Fig. S4). In four independent experiments, first and fourth instar larvae from both genotypes showed equal preference when treatments included cotton leaf discs in each arm (first instar (WT): *X*^2^ = 0.27; *P* = 0.87; fourth instar (WT): *X*^2^ = 0.043; *P* = 0.84; first instar (KO): *X*^2^ = 1.04; *P* = 0.3; fourth instar (KO): *X*^2^ = 0.024; *P* = 0.88). In another control experiment, both instars across genotypes (WT or KO) preferred cotton to a blank control (empty arm) (first instar (WT): *X*^2^ = 19.56; *P* < 0.001; fourth instar (WT): *X*^2^ = 16.95; *P* < 0.001; first instar (KO): *X*^2^ = 14.22; *P* < 0.001; fourth instar (KO): *X*^2^ = 23.52; *P* < 0.001). These results showed that both instars were similarly attracted to cotton leaf discs and confirmed that there was no behavioral difference in attraction to the discs between WT and KO larvae.

**Fig. 3:**
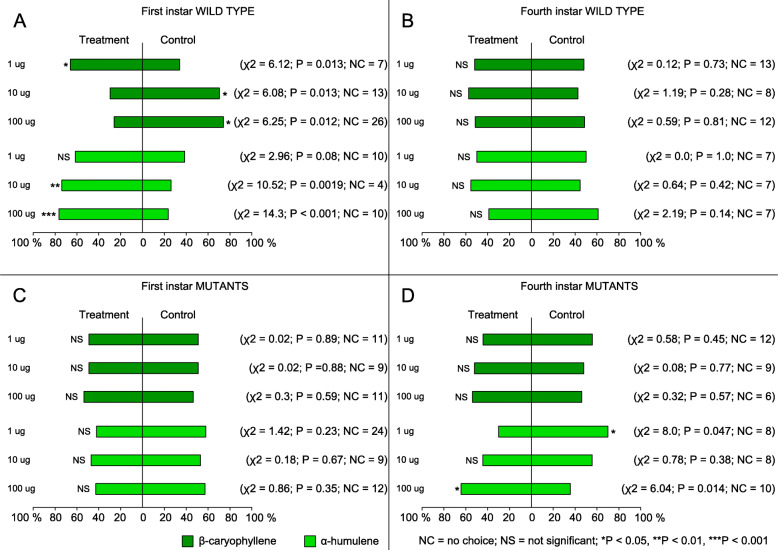
Preference of first and fourth larval instar of *Spodoptera littoralis* wild type and SlitOR40-knockout: The data show the percentage of larvae choosing the control or one of the two odors tested at three doses in a Y-tube assay: **A** Behavioral responses of first instar (WT) to *β*-caryophyllene (1/10/100 μg doses) + cotton leaf discs vs cotton leaf discs (*n* = 66, 50, and 53, respectively) and α-humulene (1/10/100 μg doses) + cotton leaf discs vs cotton leaf discs (*n* = 67, 50, and 61, respectively). **B** Behavioral responses of fourth instar (WT) to *β*-caryophyllene (1/10/100 μg doses) + cotton leaf discs vs cotton leaf discs (*n* = 88, 62, and 80, respectively) and α-humulene (1/10/100 μg doses) + cotton leaf discs vs cotton leaf discs (*n* = 63, 63, and 43, respectively). **C** Behavioral responses of knockout first instars to *β*-caryophyllene (1/10/100 μg doses) + cotton leaf discs vs cotton leaf discs (*n* = 60, 58, and 65, respectively) and α-humulene (1/10/100 μg doses) + cotton leaf discs vs cotton leaf discs (*n* = 81, 58, and 54, respectively). **D** Behavioral responses of knockout fourth instars to *β*-caryophyllene (1/10/100 μg doses) + cotton leaf discs vs cotton leaf discs (*n* = 55, 57, and 56, respectively) and α-humulene (1/10/100 μg doses) + cotton leaf discs *vs* cotton leaf discs (*n* = 58, 71, and 83, respectively). Asterisks indicate statistically significant differences following a chi-square test. (***P < 0.001; **P < 0.01; *P < 0.05). NS not significant, NC number of insects that exhibited no choice

Subsequently, we compared the behavior of both instars of WT and KO genotypes to leaf discs of cotton plants + filter paper treated with solvent (paraffin oil) versus cotton leaf discs + filter paper treated with test odorants: *β*-caryophyllene or α-humulene (treatments). In the experiments with first instar WT larvae, cotton leaf discs with 1-μg dose of *β*-caryophyllene induced preference compared to control (*X*^2^ = 6.12; *P* = 0.013) (Fig. 3A). However, with increasing doses of *β*-caryophyllene (10 and 100 μg), larvae preferred the control arm (*X*^2^ = 6.08; *P* = 0.013; *X*^2^ = 6.25; *P* = 0.012, respectively). In the experiments with α-humulene, first instar larvae preferred the two higher doses of α-humulene (10 and 100 μg) (*X*^2^ = 10.52; *P* = 0.0019; *X*^2^ = 14.13; *P* < 0.001, respectively). At the 1-μg dose, first instar larvae tended towards humulene but without statistical significance (*X*^2^ = 2.96; *P* = 0.08). Collectively, first instar larvae responded to both *β*-caryophyllene and α-humulene but dose-response patterns towards the two isomers differed. In KO behavioral tests, first instar larvae showed no preference for *β*-caryophyllene (1 μg: *X*^2^ = 0.02; *P* = 0.89; 10 μg: *X*^2^ = 0.02; *P* = 0.88; 100 μg: *X*^2^ = 0.3; *P* = 0.59) nor for α-humulene over solvent (1 μg: *X*^2^ = 1.42; *P* = 0.23; 10 μg: *X*^2^ = 0.18; *P* = 0.67; 100 μg: *X*^2^ = 0.86; *P* = 0.35) at any of the doses tested (Fig. 3C).

Fourth instar WT larvae chose equally control leaf discs and leaf discs treated with *β*-caryophyllene (1 μg: *X*^2^ = 0.12; *P* = 0.73; 10 μg: *X*^2^ = 1.19; *P* = 0.28; 100 μg: *X*^2^ = 0.59; *P* = 0.81) or α-humulene (1 μg: *X*^2^ = 0.0; *P* = 1.0; 10 μg: *X*^2^ = 0.64; *P* = 0.42; 100 μg: *X*^2^ = 2.19; *P* = 0.14) (Fig. 3B), contrary to first instar larvae.

Thus, behavioral responses in the two larval stages corroborate with *SlitOR40* expression. Knockout of SlitOR40 had no effect on fourth instar larvae behavior to *β*-caryophyllene (Fig. 2D). Interestingly, KO fourth instar larvae showed preference to α-humulene at the highest dose tested (100 μg: *X*^2^ = 6.04; *P* = 0.014) (Fig. 3D). However, larval preferences to α-humulene changed with decreasing doses; a 10-μg dose induced no preference (*X*^2^ = 0.78; *P* = 0.38), while larvae preferred the control arm compared to the one with 1 μg of α-humulene (*X*^2^ = 8.0; *P* = 0.047) (Fig. 3D).

### Electroantennography (EAG) responses

**Fig. 4:**
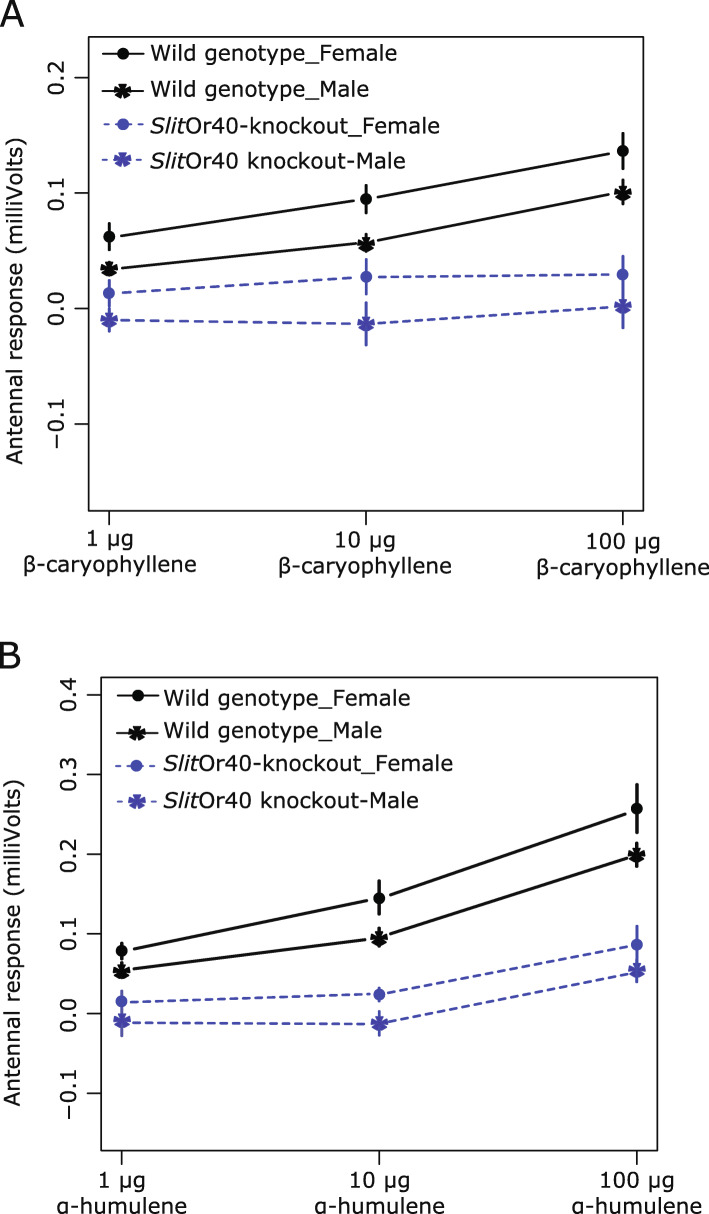
EAG responses of *Spodoptera littoralis* wild type and *Slit*Or40-knockout moth antennae to *β*-caryophyllene and α-humulene: Three doses for both compounds were tested: 1 μg, 10 μg, and 100 μg (*n* = 10 per sex). The slope of dose-response to **A** *β*-caryophyllene and **B** α-humulene are sex-independent. In KO moths, the slope of dose-response is significantly different from WT (dose*genotype interaction). The response to *β*-caryophyllene was completely abolished in KO moths, while the dose-response to α-humulene was reduced but still significantly different from WT

To confirm the effectiveness of knocking out *SlitOR40*, we recorded EAG responses from KO adult antennae in comparison to WT adult antennal responses. The EAG recordings showed no statistical difference in responses to positive control stimuli: guaiacol and (±)-linalool in both genotypes (Additional file 8: Fig. S5). However, at stimulation with SlitOR40 ligands, sex, genotype, and compound dose all significantly affected response intensity to *β*-caryophyllene and α-humulene (Fig. 4A, B; ANCOVA analysis, Table 1). There was a global tendency that female moth antennae responded stronger than male antennae, irrespective of doses applied and genotype. However, the slope of the dose-response to either compound did not depend on sex (dose*sex interaction non-significant). On the contrary, KO of *Slit*OR40 significantly modified the slope of the dose-response to *β*-caryophyllene and to α-humulene (dose*genotype interaction significant). In order to refine these results, we performed linear regression on response intensity as a function of compound dose, separately for each genotype and without taking into account the sex parameter. The WT moths showed a significant dose-response to both compounds (Fig. 4A, B; ANCOVA analysis, Table 2). In KO moth, the dose-response to *β*-caryophyllene was completely abolished (slope of the regression not significantly different from zero), while the dose-response to α-humulene was reduced but still significant (slope of the regression significantly different from zero). Therefore, as expected, *Slit*OR40 knockout affected the moth’s ability to perceive its cognate ligands: KO moths completely lost the ability to detect *β*-caryophyllene and are less sensitive than WT to α-humulene although they could still detect it.

**Table 1:** Statistical analysis of EAG response intensity to *β*-caryophyllene and α-humulene. Results of the ANCOVA analyses testing the effect of compound dose, moth sex, and moth genotype on response intensity. Text in bold indicates statistical significance

Factor	*Df*	*β*-caryophyllene			α-humulene		
		Sum of squares	*F* value	*P*-value	Sum of squares	*F* value	*P*-value
**Dose**	1	0.0531	29.2	**2.62E−7**	0.364	109	**<2E−16**
**Sex**	1	0.0259	14.2	**0.000235**	0.0313	9.41	**0.00257**
**Genotype**	1	0.197	108	**<2E−16**	0.464	139	**<2E−16**
Dose*sex	1	2.00E**−**5	1.00E**−**2	0.922	0.00130	0.404	0.526
Dose*genotype	1	0.0196	10.8	**0.00129**	0.0535	16.1	**9.73E−5**
Sex*genotype	1	8.00E**−**5	0.0460	0.831	0.00140	0.521	0.521
Triple interaction	1	1.00E**−**5	7.00E**−**3	0.935	0.00130	0.315	0.576
Residual	145	0.264			0.483

**Table 2:** Statistical analysis of EAG response intensity to *β*-caryophyllene and α-humulene. Estimation of dose-response slopes separately for each moth genotype, irrespective of sex (linear regression). Text in bold indicates statistical significance

		*β*-caryophyllene		α-humulene	
		OR40 knockout	Wild type	OR40 knockout	Wild type
Regression statistics	*F*-statistic	0.866	41.5	17.4	87.0
	*DF*	1;64	1;85	1;64	1;85
	*P*-value	0.356	**6.83E−9**	**9.41E−5**	**1.17E−14**
	*R*^2^	0.0134	0.328	0.214	0.506
Regression slope	Estimate	0.00700 ± 0.00752	0.0348 ± 0.00541	0.0339 ± 0.00814	0.0794 ± 0.00851
	*T*-value	0.930	0.00541	4.17	9.33

### Phylogenetic analysis

**Fig. 5:**
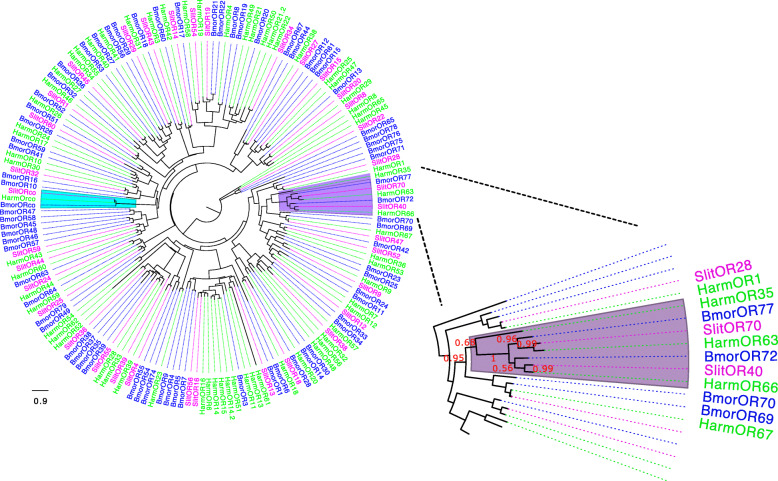
Maximum likelihood phylogenetic tree of ORs expressed in larvae of *Spodoptera littoralis*: The analyses included ORs from *S. littoralis* (purple), *B. mori* (blue), and *H. armigera* (green). Highlighted clades: *Orco* (cadet blue); SlitOR40 and SlitOR70 along with ORs from *B. mori* and *H. armigera* (lilac). Zoomed-in image: clade grouping SlitOR40, the newly identified OR SlitOR70, and four other ORs, belonging to *B. mori*: *Bmor*Or72 and *Bmor*Or77, and *H. armigera*: *Harm*Or63 and *Harm*Or66. The node support value (SH-aLRT) > 0.9 is considered well supported, 0.8–0.9 node values are considered weakly supported, and values < 0.8 indicate the node values are unsupported. The unrooted phylogenetic tree was built using the online tool PhyML 3.0

We constructed a phylogenetic tree based on the maximum likelihood method with the Bayesian information criterion (BIC) [43, 44] built using the online tool PhyML 3.0 (Fig. 5 and Additional file 9: Fig. S6). Smart Model Selection (SMS) method was used to choose the substitution model. Based on this, the “JTT+G+F” model was selected. We used amino acid sequences of ORs from larvae of *S. littoralis* along with ORs from larva and adult of *B. mori* and *H. armigera* [45–47]*.* The phylogenetic clustering of *S. littoralis* larval ORs (except newly identified SlitOR70) corroborates with previous reports [19, 31]. The OR of interest, SlitOR40, shared the same clade with SlitOR70. This well-supported clade (SH-aLRT = 1) contained four other ORs, two from *B. mori* (BmorOR72 and BmorOR77) and two from *H. armigera* (HarmOR63 and HarmOR66). These two last ORs from *H. armigera* have recently been functionally characterized, and HarmOR63 is tuned to *β*-caryophyllene and caryophyllene oxide [48]. Clustering of the SlitOR40, SlitOR70, BmorOR72, BmorOR77, HarmOR63, and HarmOR66 ORs’ topology did not change when all known ORs from *S. littoralis* [19, 31] were included in the phylogenetic analysis (Additional file 9: Fig. S6). Whether *SlitOR70* is electrophysiologically “tuned” to *β*-caryophyllene and/or α-humulene remains to be investigated.

## Discussion

In our study, we demonstrated for the first time that some ORs are differentially expressed according to larval stages in *S. littoralis*. The transcriptomic analysis conducted in this study revealed 36 ORs expressed in the first and fourth instar of *S. littoralis*. Interestingly, we did not identify any larval-specific ORs in *S. littoralis*, consistent with the data from the previous report [19], whereas larval-specific ORs have been identified in other Lepidoptera species such as *B. mori* [18].

We focused on *SlitOR*40, which we found expressed in the first, second, and third larval stages and not in the fourth larval stage. Through directed genome mutation, we knocked out the *SlitOR40* gene and revealed that larval behavior to SlitOR40 ligands is differentially disrupted dependent on the larval stages. Precisely, CRISPR-Cas9-guided *SlitOR*40-KO disrupted specific odor detection abilities in the first instar larvae and affected foraging behavior. Our study confirms that modulation in expression of *SlitOR*40 during two developmental stages influences instar-specific attraction behavior.

SlitOR40 was deorphanized and shown to respond to the terpene *β*-caryophyllene and its isomer α-humulene, two volatile molecules that are commonly produced by plants, including host plants of *S. littoralis*, as reaction to herbivory [40, 41]. The response spectrum of SlitOR40 perfectly matched with the response spectrum of one OSN profile we previously identified via single sensillum screening in *S. littoralis* female moth antenna [49–51]. This OSN type was the only one found to respond to both *β*-caryophyllene and α-humulene. Taken together, these data suggest that *SlitOR*40 is the OR expressed in this OSN type.

Behavior studies revealed that the cognate ligands, *β*-caryophyllene and α-humulene, had a significant effect on the WT first instar larval choice, while these compounds had no significant effect on the fourth instar choice. These results clearly correlate with one major finding of our study, the absence of *SlitOR*40 in the fourth instar. This provides experimental evidence for differential OR expression as a molecular basis for behavioral plasticity such as changing food preference in larvae.

Interestingly, the cognate ligands elicited opposite behavioral responses (avoidance versus attraction) in the first instar WT larvae. Our results suggest that *S. littoralis* first instar larvae could distinguish between the isomers. As one would expect that the different ligands of one OR would trigger the same behavioral response, this finding also suggests a possible dual-channel for the detection of *β*-caryophyllene and/or α-humulene. Accordingly, previous studies on adults have shown that the *S. littoralis* female olfactory system contains two separate olfactory channels: one pathway tuned to *β*-caryophyllene and α-humulene, and the other pathway to α-humulene alone, and the signals innervate two different glomeruli in the antennal lobe [11, 49, 52]. This suggests that SlitOR40 is not the only receptor involved in α-humulene detection in *S. littoralis* female and supports the existence of a similar dual olfactory channel in larvae. Interestingly, the phylogenetic analysis of larval ORs clustered *SlitOR*40 in the same clade with the newly identified *SlitOR*70 and with HarmOR63, a receptor tuned to *β*-caryophyllene and caryophyllene oxide [48]. Such functional conservation of two ORs in this clade (SlitOR40 and HarmOR63) suggests that SlitOR70 constitutes a good candidate for the second channel of α-humulene detection.

Our EAG analyses of WT and SlitOR40-KO adults also support the existence of a second OR tuned to α-humulene in *S. littoralis* since the responses to α-humulene were not totally abolished in KO mutants, as they were for *β*-caryophyllene. In larvae, the scheme was more complex: if *SlitOR*40-KO abolished larval behavior to *β*-caryophyllene in the first instar, it also abolished larval behavior to α-humulene. Thus, the contribution of the other OR to the α-humulene response appeared as negligible at this developmental stage. However, the fourth instar *SlitOR*40-KO larvae showed a significant odor-guided behavior to α-humulene that was not evidenced in WT. A plausible explanation is that knocking out *SlitOR40* may have a side effect on other OR(s), regulating or deregulating their expression, leading to a gained response to α-humulene at specific stages.

## Conclusions

Our data, grouped altogether, provide the first empirical evidence that different larval stages use different ORs, bringing molecular explanation of dynamic changes in larval olfactory behavior. Specifically, our results on *SlitOR40* clearly linked its expression to *β*-caryophyllene- and α-humulene-associated behavior in first instar larvae, but not in fourth instars. The discovery that larvae are able to respond differently to the isomers, *β*-caryophyllene and α-humulene (avoidance vs attraction), and that the associated behavior is instar-specific, adds a new dimension to our understanding of foraging behavior during larval development.

The volatiles *β*-caryophyllene and α-humulene are constitutive terpenes emitted by plants, including cotton, one of the preferred hosts of *S. littoralis* [53, 54]. While α-humulene is emitted at a low level compared to *β*-caryophyllene in undamaged plants, its emission increases during herbivory. It has been hypothesized that increasing rates of α-humulene most certainly signals herbivore-induced plant damage and therefore competition for food [55]. Both compounds have been associated with plant volatile blends that affect host plant selection for oviposition in *S. littoralis* and other moth species [56–60]. However, in some lepidopteran pests, *β*-caryophyllene and α-humulene induce different behavioral responses in female moths. For example, α-humulene stimulated oviposition in the European corn borer *Ostrinia nubilalis* (Pyralidae) when tested individually, unlike *β*-caryophyllene, which neither stimulated nor deterred it [56]. Similarly, in *B. mori* and *Cydia strobilella* (Lepidoptera; Tortricidae), α-humulene stimulated oviposition behavior, when tested individually and as a blend component [61, 62]. The behavioral responses of caterpillars to *β*-caryophyllene and α-humulene have been less investigated. One study reports that the neonate larvae of the congeneric species *S. frugiperda* are attracted to volatile blends including α-humulene induced by conspecifics’ foraging [63]. In our study, *S. littoralis* neonates avoided *β*-caryophyllene and preferred α-humulene. The ecological relevance of such differential behavior according to species, and according to instar stages as shown here, remains to be elucidated.

The previously deorphanized SlitOR4 that is tuned to (±)-linalool [36] was found in fourth but not in first instar larvae. Expression in other larval instars remains unknown and future studies are needed to clarify a possible instar-specificity and the role of (±)-linalool in larval development and behavior. Nonetheless, our results provide evidence that the molecular changes in the peripheral chemosensory system would serve changing developmental needs, reflected by larval behavioral changes. In the future, this may open new pest management strategies based on interference at adequate developmental stages for larval behavioral control.

## Methods

### Insects

*S. littoralis* lines used in this study were reared in the laboratory on a potato-based artificial diet [33, 64]. Adults and larvae were maintained at 24 ± 2 °C and 65 ± 5% relative humidity (RH) under 16:8 h light:dark (L:D) photoperiod. For the larval transcriptome, behavioral experiments, RT-PCR and RT-qPCR assays, neonate larvae (1–12 h old) and fourth instar larvae (10–11 days old) were used. For the *SitOR40 and SlitOrco* detailed RT-PCR analysis, RNA extractions were performed on second (3–4 days old) and third (7–8 days old) instar larvae. The whole larval period lasts 16–18 days, six instars in total.

### Plant material

The cotton plants (*Gossypium hirsutum* L., Malavacae) (DPL90 variety) were grown under controlled conditions at 23 ± 2 °C and 70 ± 5% RH. Sodium lamps were used as supplement to natural light with 16:8 h L:D photoperiod. Leaves from 5–6-week-old plants were used for the olfactometer experiments (leaf disc of approximately 1-cm diameter).

### Dissection/RNA extraction

Fourth instar larvae were starved for 6 h prior to dissection for consistency with behavioral experiments. For first and fourth instars, approximately 600 larval heads were collected. For adults, 50 pairs of male and female antennae were collected 3–4 days after eclosion from the pupae. For transcriptomics, first and fourth instar RNA extracts were used (one biological replicate). For RT-PCR and RT-qPCR, first to fourth instar and adult RNA extracts were used and five biological replicates were generated and used for cDNA synthesis for each of the four larval instars and adults. The TRIZOL (Invitrogen) extraction method was used to isolate the RNA from the target tissues, as previously described [31].

### Illumina sequencing, processing, assembly, and annotation

Approximately 2 μg of total RNAs from first and fourth instar larvae were used for Illumina sequencing at BGI-Tech (China). Paired-end Illumina TruSeq libraries were generated from purified RNA using Illumina’s sample preparation kit (Illumina Inc., San Diego, CA) according to the manufacturer’s instructions and sequenced at a HiSeq 2000 platform. All raw data from this study have been deposited at NCBI (SRA accession: PRJNA507834). Reads were de novo assembled [65] in one transcriptome that contained first and fourth instar larval reads along with published reads data from adult antennae [31] (NCBI Accession: PRJNA312160, Sample Accessions: SRX2750986-SRX2750991) to provide a more robust reference for OR expression. Following the procedure described in Walker et al. [31], we removed low-quality reads and adapter contamination from the raw reads using trimmomatic tool (version 0.32) with the following parameters: ILLUMINACLIP (sequencing adapters file: TruSeq3-PE. fa:2:30:10) and TRAILING:20. Following this, Trinity pipeline (version 2.0.6) was used on the filtered data with default parameters [66]. The resulting Trinity.fasta files were then processed using the cd-hit-est tool (version 4.6.1) [67] to reduce transcript redundancy; in transcripts sharing sequence identity ≥ 95%, the longest sequence was retained and used for further analysis. Individual reads from first and fourth instar larvae were used for read mapping on the assembled full transcriptome using bowtie (version 0.12.6) [68]. The completeness of the assembled transcriptomes was assessed using the BUSCO tool (Benchmarking Universal Single-Copy Orthologs, version 3.0.2) (https://busco.ezlab.org/), against the insecta_obd9 dataset that included 1658 reference genes [35]. The transcripts’ expression level abundances were estimated using RSEM [69], and the mapping statistics were calculated using Samtool [70] for downstream analyses.

To identify the expressed ORs, we conducted a tBlastn search on the assembled transcriptome using an input file containing protein sequences of ORs previously identified in *S. littoralis* [19, 31], with an *e*-value cut-off of 1e−05. The putative OR transcripts were translated into protein sequences with the ExPASy portal [71] and were then aligned to reference transcripts to identify similarity or divergence using Clustal [72]. Newly identified *SlitOR*s were added to the query dataset to conduct an additional tBlastn query.

### RT-PCR and quantitative real-time PCR (RT-qPCR)

To prepare cDNAs from larval heads and adult antennae, total RNAs (1 μg) were reverse transcribed using the Invitrogen; ThermoScript^TM^ Reverse Transcriptase kit as described in the manufacturer’s protocol.

RT-PCR was performed using gene-specific primers (Additional file 10: Table S4). For all RT-PCR expression assays, 1 μL of cDNA was used with 1 μL of 10 mM of forward and reverse primers along with Dreamtaq buffer, dNTPs, and Dreamtaq enzyme making up the final volume of 25 μL. The RT-PCR amplification was as follows: 95 °C for 2’; 36 cycles X (95 °C for 30”, 53.5–65 °C (depending on primers) for 30”, 72°C for 1’ 30”, 72°C for 10’). The PCR-amplified products were run on a 1.5% agarose gel for verification.

RT-qPCR was also performed using gene-specific primers (Additional file 10: Table S4) and the amplicon length was between 100 and 200 bp. The amplification peak and the subsequent melting curve were checked to ensure primer specificity. Each RT-qPCR reaction was run in triplicates on five independent biological replicates and contained 10 μL of SYBR Green reagent (Invitrogen), 1 μL of 10 mM forward and reverse primers, 7 μL of RNase-free water, and 2μL of cDNA (5 ng/μL concentration cDNA), making the final volume of 20 μL. The reactions were performed in a 96-well plate (iQ5 qPCR System; plate type-white, Bio-Rad), and the amplification was as follows: initial denaturing step at 95 °C for 5’; 40 cycles X (95 °C 15”, annealing step 59–61 °C 25” (based on primer pairs)) 95 °C 10”, 50 to 95 °C (increment 0.5 °C) for 5”. We considered *Orco* as a relative reference to compare *SlitOR40* expression in the larvae (heads) and male antennae, and *β*-actin, L13A, and Ef1A to compare *Orco* expression in first and fourth larval instars. For all the runs, relative expression was calculated using the 2^−ΔΔCt^ (cycle threshold) method [73], and the geometric mean of expression values of the three reference genes were considered.

### TOPO/gateway cloning of *SlitOR*40 and heterologous expression

The open reading frame (ORF) was amplified from larval cDNA using forward (F = GGAAGAGCTGCCTGAAATTTCAAAAGA) and reverse (R = CGACCAAGTTGTGCTCAGTAC) primer sequences and cloned into PCR8/GW/TOPO plasmids (Thermo Fisher Scientific), according to the manufacturer’s protocol. *SlitOR*40 was further cloned into the pUAST.attB plasmid using Clonase II enzyme mix kit (Thermo Fisher, Scientific, USA). The OR insert was sequenced using UAS1 and UAS2 sequencing primers [74] to confirm desired sequence and insertion orientation. The UAS-*SlitOR*40 *Drosophila melanogaster* lines were generated by BestGene, Inc (Chino Hills, CA, USA), using the PhiC31-mediated integration approach. Briefly, upon sequence confirmation, the recombinant pUAST-HA.attB-*SlitOR*40 plasmid containing a *attB* site was injected into *attP* docking site with PhiC31 activity within the third chromosome (genotype y1 M{vas-int.Dm}ZH- 2A w∗; M{3xP3-RFP.attP}ZH-86Fb) of the embryos of a fly. The UAS fly lines were then crossed with Or22a-GAL4 promoter flies that lack the endogenous OR genes *OR*22a and *OR*22b, following the crossing scheme in Gonzalez et al. [74]. The flies generated with genotype ∆*halo*; Or22a-GAL4/UAS-*SlitOR*40 were used for downstream electrophysiological recordings.

### CRISPR/Cas9 egg injection

Two RNA guides were designed against exon 1 (gRNA1 sequence: TAGAGGAGAATTACAACTTG AGG) and exon 2 (gRNA2 sequence: TGCAGCTGGGAGGTATGTGG AGG) using the CRISPOR gRNA design tool (cripsor.tefor.net) [75] and the *SlitOR40* genomic DNA sequence as target. Guide syntheses and Cas9 protein production were performed as previously described in Koutroumpa et al. [76]. The two gRNAs were injected together along with the Cas9 protein, in order to create more aggressive mutations with a higher probability to knockout (KO) the gene due to large deletions. Another advantage of these large deletions is that they can be visible on agarose gel with no need of further genotyping. Concentrations of the gRNAs were 45 μM and the Cas9 was 30 μM (Sp-Cas9-NLS-GFP-NLS; fusion of *Streptococcus pyogenes*-derived Cas9 protein and green fluorescent protein between nuclear localization signal on both N and C terminal of the protein). The gRNA:Cas9 ratio in the complex was 1.5:1. Aliquots of gRNA were denatured at 80°C for 2 min and then left on ice for 2 min before mixing them with the necessary amount of Cas9. Each complex was formed at room temperature during 10 min. Mix of the two gRNA:Cas9 complexes was done afterwards in order to avoid eventual Cas9 binding preferentially to one gRNA. The mix was then placed on ice until use. *S. littoralis* eggs were prepared for injection as described in Koutroumpa et al. [76]. Injections took place within one half to 1 h after oviposition in order to target the first steps in embryogenesis. We used an Eppendorf FemtoJet 4i injector.

Crude genomic DNA was extracted from one larval pseudopod (Wizard Genomic DNA Purification Kit, Promega, Madison, WI). Amplification of the gDNA was performed using the *Pfu* thermostable polymerase (Promega, Madison, WI) and specific primers for *SlitOR40.* The forward primer (TCGAACCGATATTACCATGTCTG) sitting on exon 1 and the reverse primer (ACTTTAGTCTCCTCAGTAACGT) on exon 2 amplifies a PCR product of 684 bp and includes the target sequence. First, the gDNA was denatured at 95°C for 2 min. The amplification program was 45 s at 95°C, 30 s at 62°C, and 60 s at 72°C, and this was repeated 40 times. Agarose gel analysis gave size polymorphism differences among individual amplifications, and sequencing (Biofidal, Vaulx-en-Velin, France) confirmed the gel results. Sequences were aligned and manually curated using SEQUENCHER™ 4.7 (Gene Codes Corporation, Inc.).

Injected eggs were kept at the same conditions as the regular rearing. G0 adults carrying mutagenic events were backcrossed with adults from the regular rearing (called here wild type, WT). G1 heterozygote males and females carrying the same mutation were crossed to obtain homozygote G2 *SlitOR4*0 knockouts (KO). One G2 homozygote CRISPR line was chosen for further analysis. Adults were tested for their response in EAG and larval behavior was tested in olfactometer assays.

### Olfactometer assays

Y-tube olfactometer tests were performed to examine the behavior of WT and *SlitOR*40-KO larvae. Olfactometers of different dimensions were selected according to different sizes of tested larvae. For fourth instar larvae, the size of the olfactometer that we designed was arm length = 14 cm, stem = 12.5 cm, and inner diameter = 2.2 cm, while for the neonate larvae, the olfactometer size was arm length = 4 cm, stem = 5 cm, and inner diameter = 0.8 cm (built at Humiglas, Södra Sandby, Sweden). For both systems, control experiments confirmed adequate settings with larvae showing equal upwind walk towards cotton leaf discs on both sides as well as discrimination between the arms providing leaf discs alone and leaf discs with control stimulus (Additional file 7: Fig. S4). Experiments were performed under diffused light (53 lux) from the top of the olfactometer. A charcoal-filtered airstream was pumped (0.1 L/min for first instar and 0.2 L/min for fourth instar) through a wash bottle containing 50 mL distilled water for humidification and then split into each arm of the olfactometer.

To achieve context-based attraction, each arm of the olfactometer had two cotton leaf discs (each 1-cm diameter) on a wet filter paper (Grade 1002, Munktell Filter AB, Munktell) as a background. Then, filter paper in one arm of the olfactometer was treated with treatment solution (*β*-caryophyllene or α-humulene) diluted in paraffin oil on a 1-cm^2^ filter paper (10 μL), versus control (10 μL paraffin oil). The behavior of WT and *SlitOR*40-KO larvae was tested with three different doses (1, 10, and 100 μg) of *β*-caryophyllene or α-humulene.

The fourth instar larvae were starved for 6–8 h prior to the experiment. Larvae were tested individually for 10 min. Larval responses were recorded as “treatment” or “control” based on their choices, and the larvae that did not enter the olfactometer arms within 10 min were treated as “no-choice.” To avoid any positional effect, treatment position was swapped between the arms of the olfactometer every fifth larva, and 10–15 larvae were tested per day. An adjusted protocol was followed for the first instar larvae. After hatching, larvae feed on the egg residues, and after approximately 45 min to 1 h, start moving towards food sources. Those larvae were selected for the behavioral assays. First instar larvae required a longer “activation time” to start moving in the olfactometer compared to fourth instar larvae. Therefore, first instar larvae were tested for 20 min.

The percentage attraction was calculated as behavioral response = (number of larvae in the arm with treatment/total number of larvae that made a choice) multiplied by 100.

### Electrophysiological recordings

#### Single sensillum recording (SSR) on *D. melanogaster* neurons expressing SlitOR40

We functionally characterized the *SlitOR40* receptor using the *Drosophila* empty neuron system [39, 77]. Four- to 5-day-old *D. melanogaster* female flies carrying *SlitOR40* receptors expressed in ab3 sensillum “A” neurons were used for electrophysiological recordings. Recordings were repeated with six different flies. Prior to recording, a fly was immobilized in a 100-μL pipette tip with its antennae protruding outside the narrow tip. This tip was mounted on a glass slide positioning the fly ventrally, and the right antenna was gently pulled flat over the glass coverslip. The antenna was held by a micro-capillary at the flagellum to avoid antennal movement. The glass slide was gently placed under the microscope (Olympus BX51W1) across a glass tube (positioned approximately 20 mm from the fly antenna) connected to charcoal-filtered humidified main airflow (1 L/min). At the lateral side of the glass tube, a hole allowed the introduction of a disposable glass Pasteur pipette (150 mm, Assistent, # 567/1, Germany) containing the odorant on the filter paper. A reference electrode was gently inserted into the eye using a motor-controlled piezo micromanipulator (Märzhauser DC-3K, Wetzlar, Germany), while a recording microelectrode was inserted into the base of the ab3 sensilla. The extracellular action potentials from the sensory neuron were recorded using tungsten microelectrodes, sharpened using KNO_2_-solution. The signal was then amplified (INR-02A and AC/DC UN-06, respectively) and transferred to a computer through IDAC4 (Intelligent Data Acquisition Controller-4) for visualization. The analysis was performed using Autospike (version 3.4) software (Syntech, Kirchzarten, Germany).

Odorant compounds were diluted in paraffin oil (Merck) except 1-indanone, carvacrol, and 4,8-dimethyl-1,3,7-nonatriene (DMNT), which were diluted in hexane (LabScan) to a concentration of 10 μg/μL (Additional file 11: Table S5). A disposable glass Pasteur pipette containing 1.5 × 1 cm filter paper (size Ø 90mm; grade 1002; Munktell) was then loaded with 100 μg (diluted in 10 μL of paraffin oil or hexane) of the odorant compound to deliver the stimulus (since there was a very low electrophysiological response to most of the odorants, lower doses were not tested). *β*-Caryophyllene (chemical purity = 98.5%) and α-humulene (chemical purity = 96%) were tested at the dose of 10 μg (diluted in 10 μl of paraffin oil). Disposable Pasteur pipettes loaded with individual odorants were used in order to deliver odors using stimulus controller (Syntech SFC-1/b; 2.5 ml air flux) onto the fly antenna, from which responses were recorded for 0.5 s post-stimulation.

### Electroantennography (EAG)

We performed EAG measurements on adult antennae to determine olfactory responses from WT and KO moths to *SlitOR40* cognate ligands. Two other compounds, guaiacol and (±)-linalool, were included as positive control stimuli, as they are known to be ligands of SlitORs expressed in adult antennae [36, 78]. All odorants were diluted in hexane. Three doses of *β*-caryophyllene and α-humulene (100 μg, 10 μg, and 1 μg) and one dose (10 μg) of each positive control stimulus were tested.

The excised moth antennal base was placed at the tip of a glass electrode (filled with Beadle-Ephrussi Ringer solution) [79] connected to a 10x preamplifier probe that was linked to an IDAC-2 box (Syntech, Kirchzarten, Germany), while the distal end of the antenna was connected to another glass electrode for grounding. The electrodes were placed 1 cm away from an odor-delivery glass tube connected to a humidified charcoal-filtered airstream (1 L/min). A disposable Pasteur pipette (VWR International, Stockholm, Sweden) containing filter paper strip (1.5 × 0.5 cm; Whatman®) was loaded with 10 μL of odorant, which was delivered (0.5 s) on the moth antenna. The signal from the antenna was amplified and transferred to a computer for visualization using the Autospike program (version 1.2.5, Syntech).

### Phylogenetic analysis

Two phylogenetic trees were built from *S. littoralis* ORs: one with larval expressed ORs, and the other with all described ORs [19, 31], along with OR sequences from *B. mori* (71 ORs from larva and adults) [45, 47] and *H. armigera* (66 ORs from larva and adults) [46]. All amino acid sequences were aligned using MAFFT (version 7) (https://mafft.cbrc.jp/alignment/server/) [80, 81], and an unrooted phylogenetic tree was built using PhyML 3.0 (http://www.atgc-montpellier.fr/phyml/) [43, 82] using BioNJ algorithm and Maximum Likelihood Tree with *Smart Model Selection* (SMS) method [44]. This software tool integrated into the PhyML web server automatically selects the best substitution model. This method chose the “JTT+G+F” substitution model. PhyML uses both NNI (Nearest Neighbor Interchanges) and SPR (Subtree pruning and regrafting) method to rearrange and optimize the tree structure. Clade support for maximum likelihood analysis was assessed using Shimodiara-Hasegawa-approximate likelihood ratio test (SH-aLRT) [43, 83]. The nodes with support value SH-aLRT > 0.9 were considered well supported, nodes with value ranging from 0.8 to 0.9 were considered weakly supported, and node values < 0.8 were considered unsupported [43]. Consensus Newick format tree was visualized and processed in FigTree software (version 1.4.4) (http://tree.bio.ed.ac.uk/software/figtree/).

### Statistical analysis

All statistical analyses were performed in R [84]. Behavioral responses of first and fourth instar larvae to different doses of *β*-caryophyllene and α-humulene were analyzed with binomial GLM to test the factors (instar, genotype, concentration of doses) influencing larval behavior. To analyze RT-qPCR data from larvae and adults, we checked for normal distribution and performed paired *t*-test on ΔCt values. EAG responses of WT and *SlitOR*40-KO adult moths to (±)-linalool and to guaiacol followed a normal distribution and were analyzed with ANOVA. To analyze EAG responses of moth antennae to *β*-caryophyllene and α-humulene (three doses of each compound), we performed ANCOVA to test the influence of doses, sex, and genotype.

## Supplementary Information

**Additional file 1: Table S1**. BUSCO scores for transcriptomes assembly completeness: Assessment of *Spodoptera littoralis* larval transcriptomes assembly completeness using the Benchmarking BUSCO tool performed against insecta_obd9 database consisting of 1,658 BUSCOs.

**Additional file 2: Table S2**. Expression profile of *Spodoptera littoralis* ORs (*Slit*ORs) in the first and fourth instar larval heads. Expression levels of ORs are displayed as FPKM values.

**Additional file 3: Table S3**. Newly identified *Slit*OR70 amino acid sequence.

**Additional file 4: Figure S1**. RT-PCR gel images of PCR-amplified ORs at different developmental stages of *Spodoptera littoralis*. Comparison included negative-control (control), first instar heads (*Slit*_1), fourth instar heads (*Slit*_4), male antennae (*Slit*_♂) and female antennae (*Slit*_♀) with 1kb gene ruler ladder (Thermo Fischer Scientific). Red rectangles around SlitOR4 and SlitOR40 represent differential expression in the first and fourth instar, respectively, supported by RT-PCR and transcriptomic analysis.

**Additional file 5: Figure S2**. Expression pattern (RT-PCR) and relative expression levels (RT-qPCR) of *Orco* and *SlitOR40* across different instars. (A) RT-PCR products obtained from *SlitOrco* and *SlitOR40* amplification from the *c*DNA of first, second, third and fourth instar larval heads and adult antennae. (B) Quantitative RT-qPCR expression levels (relative fold-change) of *Orco* in first instar larval heads, fourth instar larval heads and adult male antennae using *β*-actin, L13A and Ef1A as reference genes. Male antennae samples expressed significantly higher levels of *Orco* compared to samples from first and fourth instar larvae (Df = 28, t= -22.622, P<0.001; Df = 28, t= 50.441, *P* < 0.001, respectively). Similarly, in the fourth instar larvae, *Orco* expression was significantly higher compared to first instar (Df = 28, t = 9.33, *P* < 0.001). (C) *SlitOR40* expression (using *Orco* as reference gene) in the first instar larval heads, fourth instar larval heads and adult male antennae. Relative expression of *SlitOR40* in first instar larvae was significantly higher compared to adult male antennae (Df = 28, t = 26.532, *P* < 0.001), while in the fourth larval instar, *SlitOR40* expression was not detected (Df = 28, t = 27.693, *P* < 0.001). The analyses are based on five biological samples. The bars indicate standard errors. Statistical differences between treatments were calculated using a non-paired Student’s *t*-test. *: *P* < 0.05.

**Additional file 6: Figure S3**. *SlitOR40* CRISPR/Cas9 induced indels (insertion and deletion). (A) Wild Type (WT) *c*DNA sequence including exon1 and exon2. An asterisk separates the two exons’ sequences. Blue letters indicate the primer sequences used for genotyping. Orange and purple boxes surround sequence regions where the Cas9 cleaved *SlitOR40* and correspond to the same color boxes in B. (B) The three most prominent double mutational events obtained are shown around each of the two CRISPR guides. Indels’ produced between the two guides are described (NA means not applied) and insertion sequences are indicated in red letters. The number of nucleotide differences (Δ) between the WT line and the three mutant obtained lines are given between brackets. In both A and B, green letters show the CRISPR guide sequences with their PAM highlighted in yellow. (C) Electrophoregram traces for each of the three main double mutations obtained (BioEdit). Line 10 mutants were chosen for further crossing and phenotypic analysis.

**Additional file 7: Figure S4**. Attraction of *Spodoptera littoralis* wild type and SlitOR40-knockout larvae to positive control odors: Attraction of first and fourth instar WT and KO genotypes to positive control odors (CLD, cotton leaf discs) in both arms of the olfactometer (first instar WT: n = 44; fourth instar WT: n = 24; first instar KO: n = 59; fourth instar KO: n = 43) (top bar in Fig. S4A, S4B, S4C and S4D, respectively). Similarly, behavioral responses of WT and KO genotypes of *S. littoralis* first and fourth instar to the odor of cotton leaf discs *versus* clean air (control blank) (first instar WT: n = 50; fourth instar WT: n = 48; first instar KO: n = 21; fourth instar KO: n = 32) (bottom bar in Fig. S4A, S4B, S4C and S4D, respectively).

**Additional file 8: Figure S5**. EAG responses of *Spodoptera littoralis* wild type and SlitOr40-knockout moth antennae to positive control odors: The EAG responses of WT and KO line adult moth antennae to positive control stimuli (guaiacol and (±)-linalool) were tested at 10 μg dose (minimum n = 9 per sex). There was no statistical difference in responses to positive control stimuli, guaiacol (ANOVA: Sex: Df = 1; F = 1.63; P = 0.2; Genotype: Df = 1; F = 3.55; P = 0.066; Interaction: Df = 1; F = 0.13; P = 0.72; Residual Df = 47) and (±)-linalool (ANOVA: Sex: Df = 1; F = 0.66; P = 0.42; Genotype: Df = 1; F = 0.812; P = 0.372; Interaction: Df = 1; F = 1.68; P = 0.202; Residual Df = 47) in the antennae from both genotypes. The boxplots represent mean values along with the minimum and maximum values.

**Additional file 9: Figure S6**. Maximum-likelihood phylogenetic tree of ORs expressed in larvae and adults of *Spodoptera littoralis*: The analyses included all described ORs from *S. littoralis* (purple), *B. mori* (blue) and *H. armigera* (green). Highlighted clades: *Orco* (cadet blue); SlitOR40 and SlitOR70 along with ORs from *B. mori* and *H. armigera* (lilac). The unrooted phylogenetic tree was built using online tool PhyML 3.0.

**Additional file 10: Table S4**. Primers used in the study: List of all primers used to confirm the expression of *SlitORs*, qRT-PCR assays in first and fourth larval instars, and primers used for genotyping.

**Additional file 11: Table S5**. List of chemical compounds used in the electrophysiological recordings.

**Additional file 12:** Supplementary data.

## Data Availability

Transcriptomics data are deposited in the public biomedical database, NCBI under the following accession number: PRJNA507834. All of the data on which conclusions rely in this study are included in this published article and its supplementary information files.

## Abbreviations

ORs: Odorant receptors
IRs: Ionotropic receptors
CRISPR: Clustered regularly interspaced short palindromic repeats
Cas9: CRISPR-associated protein 9
*S. littoralis*: *Spodoptera littoralis*
*S. frugiperda*: *Spodoptera frugiperda*
*B. mori*: *Bombyx mori*
*H. armigera*: *Helicoverpa armigera*
*D. melanogaster*: *Drosophila melanogaster*
OSNs: Olfactory sensory neurons
ORCO: Odorant receptor co-receptor
RNA: Ribonucleic acid
DNA: Deoxyribonucleic acid
RNA-Seq: RNA sequencing
RT-PCR: Real-time polymerase chain reaction
qRT-PCR: Quantitative real-time polymerase chain reaction
BUSCO: Benchmarking Universal Single-Copy Orthologs
sgRNA: Single guide RNA
Bp: Base pairs
WT: Wild type
KO: Knockout
CLD: Cotton leaf disc
μg: Microgram
ANOVA: Analysis of variance
ANCOVA: Analysis of co-variance
EAG: Electroantennogram
SSR: Single sensillum recording
BIC: Bayesian information criterion
L:D: Light:dark
RH: Relative humidity
Sp-Cas9-NLS-GFP-NLS: *Streptococcus pyogenes*-derived Cas9 protein and green fluorescent protein between nuclear localization signal on both N and C terminal of the protein
DMNT: 4,8-Dimethyl-1,3,7-nonatriene
IDAC: Intelligent Data Acquisition Controller
KNO2: Potassium nitrite
