# Supplementary material for: Stage-specific expression of an odorant receptor underlies olfactory behavioral plasticity in Spodoptera littoralis larvae
Source: BMC Biol. 2021 Oct 28;19:231. doi: 10.1186/s12915-021-01159-1 (PMC8555055; doi:10.1186/s12915-021-01159-1)
Supplement: Supplementary file 1 — Additional file 1: Table S1. BUSCO scores for transcriptomes assembly completeness: Assessment of Spodoptera littoralis larval transcriptomes assembly completeness using the Benchmarking BUSCO tool performed against insecta_obd9 database consisting of 1,658 BUSCOs. [file 12915_2021_1159_MOESM1_ESM.pdf]

| | Assembled transcripts from *S. littoralis* larvae (First and fourth instar) and adults |
|---|---|
| Complete BUSCOs (C) | 1598 (96.4 %) |
| Complete and Single-Copy BUSCOs (S) | 1245 (75.1 %) |
| Complete and duplicate BUSCOs (D) | 353 (21.3 %) |
| Fragmented BUSCOs (F) | 47 (2.8 %) |
| Missing BUSCOs (M) | 13 (0.8 %) |
| Total BUSCO groups searched | 1658 |
