# Supplementary material for: Stage-specific expression of an odorant receptor underlies olfactory behavioral plasticity in Spodoptera littoralis larvae
Source: BMC Biol. 2021 Oct 28;19:231. doi: 10.1186/s12915-021-01159-1 (PMC8555055; doi:10.1186/s12915-021-01159-1)
Supplement: Supplementary file 2 — Additional file 2: Table S2. Expression profile of Spodoptera littoralis ORs (SlitORs) in the first and fourth instar larval heads. Expression levels of ORs are displayed as FPKM values. [file 12915_2021_1159_MOESM2_ESM.pdf]

Annotation	1st Instar (FPKM)	4th Instar (FPKM)	Annotation	1st Instar (FPKM)	4th Instar (FPKM)	Annotation	1st Instar (FPKM)	4th Instar (FPKM)
SlitOR1	**0.19**	**0.00** *	SlitOR21	0.00	0.00	SlitOR41	0.00	0.00
SlitORco	**4.99**	**0.34**	SlitOR22	**0.00** *	**0.00** *	SlitOR42	0.00	0.00
SlitOR3	0.00	0.00	SlitOR23	0.00	0.00	SlitOR43	**0.00** *	**0.00** *
SlitOR4	0.00	**0.13**	SlitOR24	**0.00** *	**0.00** *	SlitOR44	**0.24**	**0.00** *
SlitOR5	0.09 #	0.00	SlitOR25	**0.97**	**0.23**	SlitOR45	**0.79**	**0.50**
SlitOR6	0.00	0.00	SlitOR26	0.11 #	0.00	SlitOR46	0.67 #	2.68 #
SlitOR7	**0.00** *	**0.00** *	SlitOR27	**0.22**	**0.06**	SlitOR47	**0.33**	**0.31**
SlitOR8	**0.00** *	**0.00** *	SlitOR28	**0.55**	**0.05**	SlitOR48	0.00	0.00
SlitOR9	**0.70**	**0.17**	SlitOR29	**0.05**	**0.11**	SlitOR49	0.00	0.00
SlitOR10	2.85 #	2.98 #	SlitOR30	0.04 #	0.00	SlitOR50	0.00	0.00
SlitOR11	0.00	0.00	SlitOR31	6.79 #	8.26 #	SlitOR51	0.11 #	0.00
SlitOR12	**0.00** *	**0.00** *	SlitOR32	**0.10**	**0.00** *	SlitOR52	**0.64**	**0.00** *
SlitOR13	0.00	0.00	SlitOR33	0.00	0.00	SlitOR53	0.00	0.00
SlitOR14	**10.59**	**11.78**	SlitOR34	**0.04**	**0.00** *	SlitOR54	**0.29**	**0.00** *
SlitOR15	**0.23**	**0.00** *	SlitOR35	0.00	0.00	SlitOR55	**0.14**	**0.07**
SlitOR16	**0.69**	**0.48**	SlitOR36	**0.05**	**0.00** *	SlitOR56	**0.00** *	**0.00** *
SlitOR17	0.11 #	0.00	SlitOR37	1.19 #	0.11 #	SlitOR57	0.00	0.00
SlitOR18	**0.00** *	**0.00** *	SlitOR38	**0.00** *	**0.00** *	SlitOR58	0.77 #	0.09 #
SlitOR19	**1.12**	**0.00** *	SlitOR39	**0.15**	**0.00** *	SlitOR59	**0.05**	**0.00** *
SlitOR20	**0.00** *	**0.05**	SlitOR40	**0.19**	0.00	SlitOR60	**1.44**	**0.00** *
						SlitOR70	**0.00** *	**0.00** *

# Indicates false positive; * Indicates false negative; ORs in bold were confirmed by RT-PCR to be expressed in larvae.
