# Supplementary material for: Stage-specific expression of an odorant receptor underlies olfactory behavioral plasticity in Spodoptera littoralis larvae
Source: BMC Biol. 2021 Oct 28;19:231. doi: 10.1186/s12915-021-01159-1 (PMC8555055; doi:10.1186/s12915-021-01159-1)
Supplement: Supplementary file 3 — Additional file 3: Table S3. Newly identified SlitOR70 amino acid sequence. [file 12915_2021_1159_MOESM3_ESM.pdf]

Newly identified *Slit*OR70 amino acid sequence

>SlitOR70
MEVLKDFPEDFAKALKTSFEMLKNFNVRYLNEEQPFLKKYWRYSYIFATIFIHGFSMSIHMPELLT
GDEMTQFAYLIPSILVTIHAIFKSIVLIPMTRQISTFISELGSLWRVKFTEKQFEDKDAVLWRLDFI
NRASYWVTLSGSAQYLLSPLFETLFRRFILKQDCKLLLPFASVFPLDHTKNWLFYLLVYIFQLYSMF
LLVSMYTGAALIMISSCALLGAEFLMLKDDLSRVIPRNNNEIHNSDNTNEGDNNDNELTIEEFVK
RHQMLLGLSRQLDNVFNGMVFIDLLFVGIT
