# Supplementary figures and images for: Stage-specific expression of an odorant receptor underlies olfactory behavioral plasticity in Spodoptera littoralis larvae
Source: BMC Biol. 2021 Oct 28;19:231. doi: 10.1186/s12915-021-01159-1 (PMC8555055; doi:10.1186/s12915-021-01159-1)

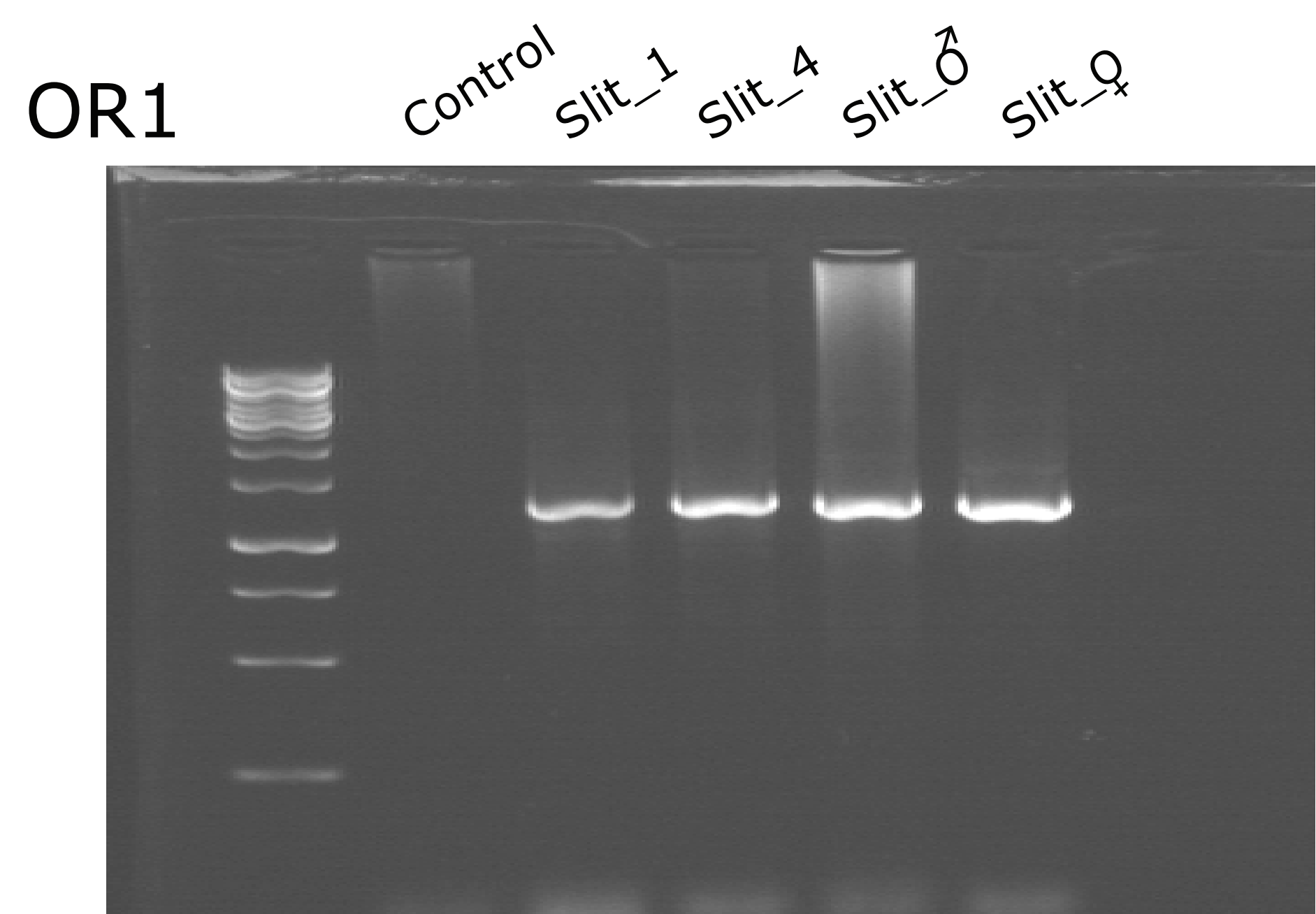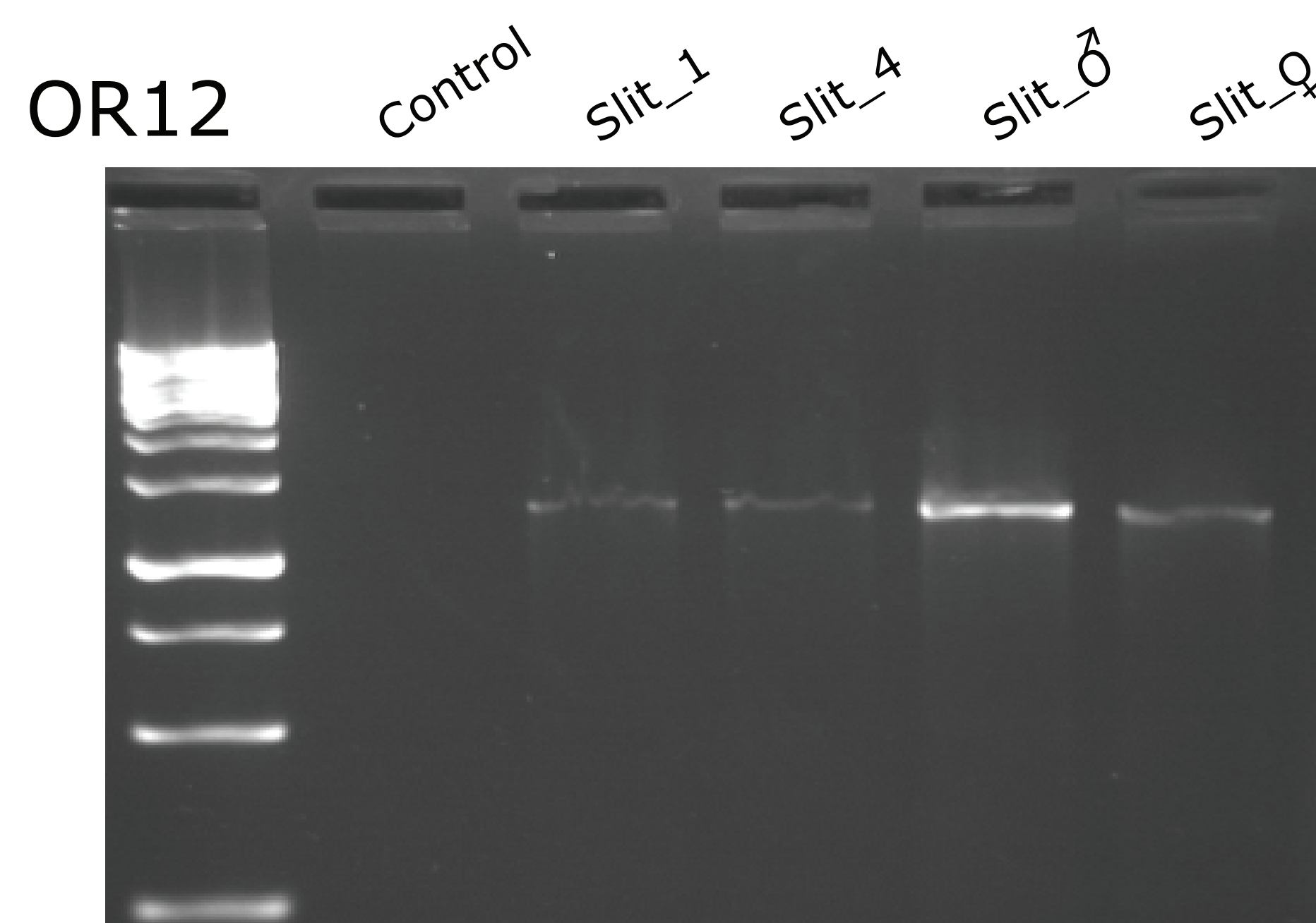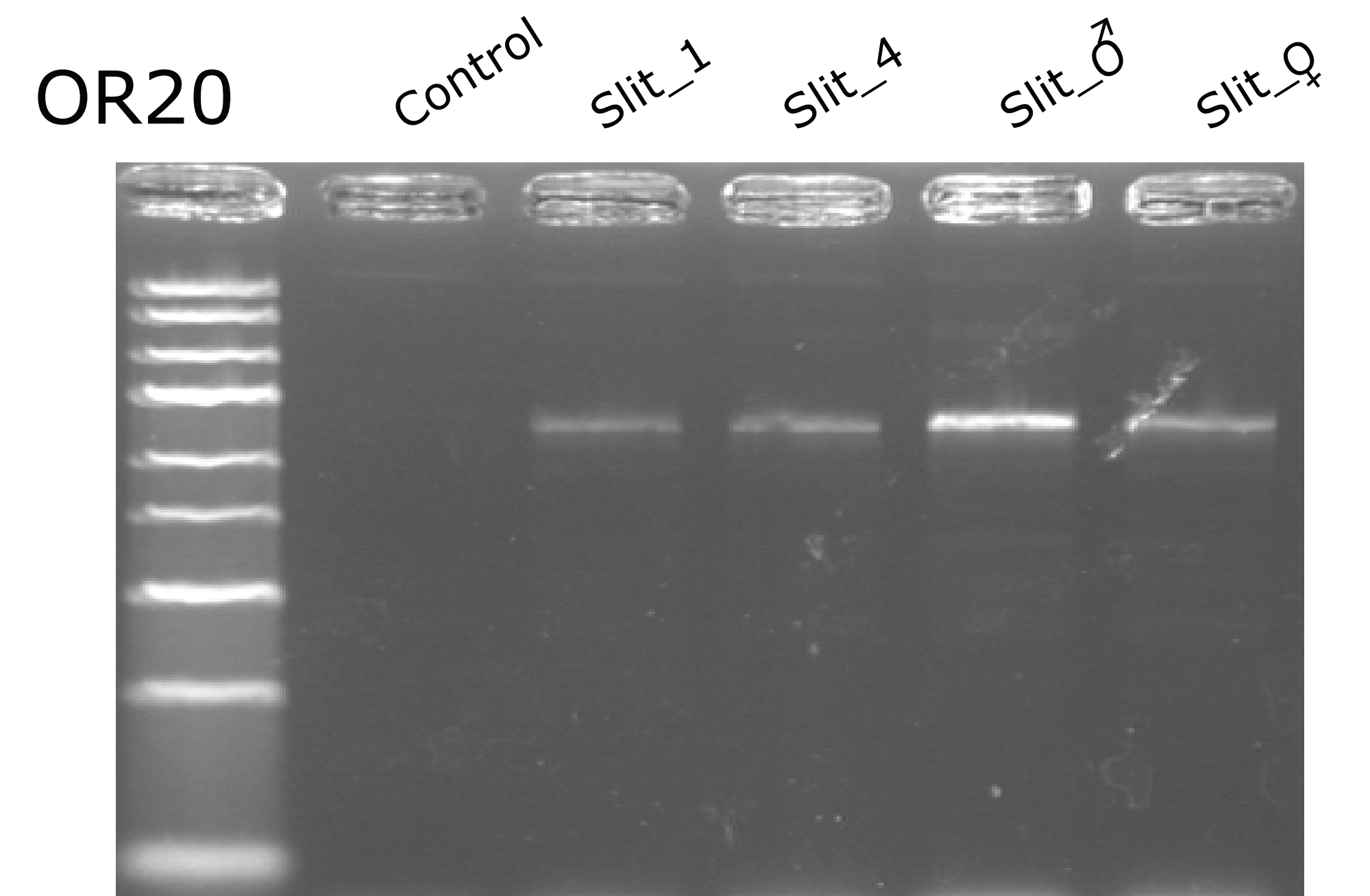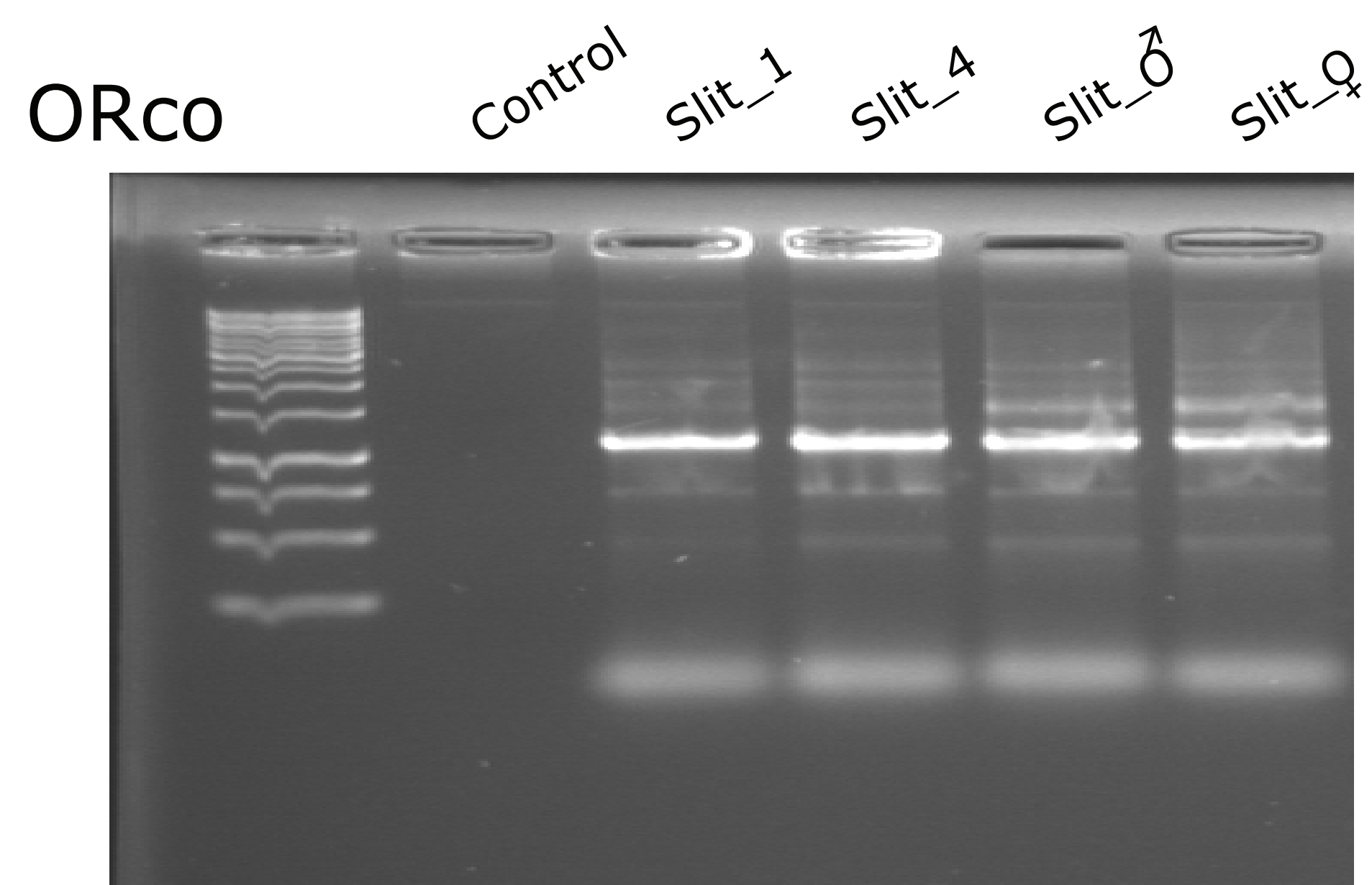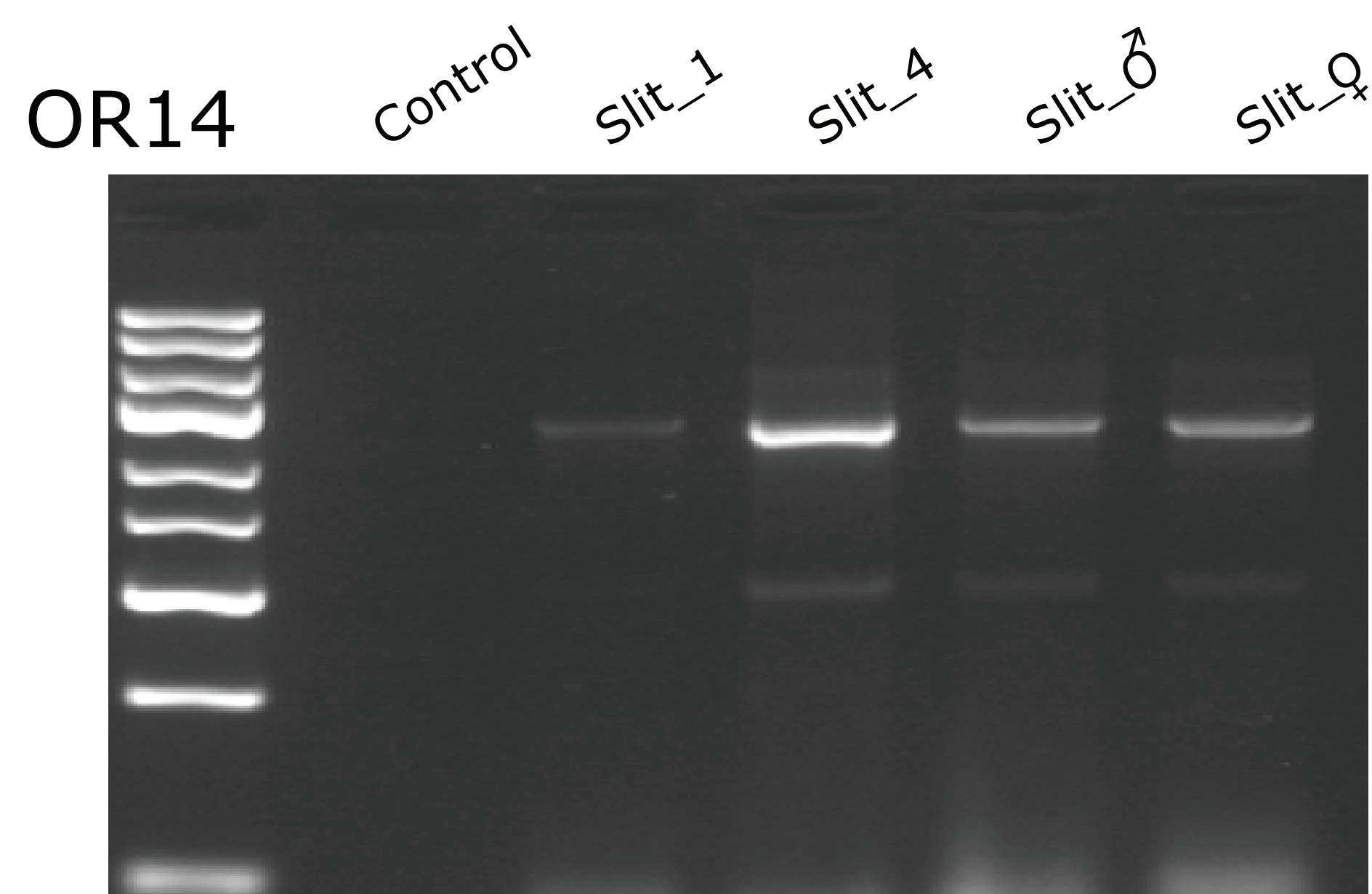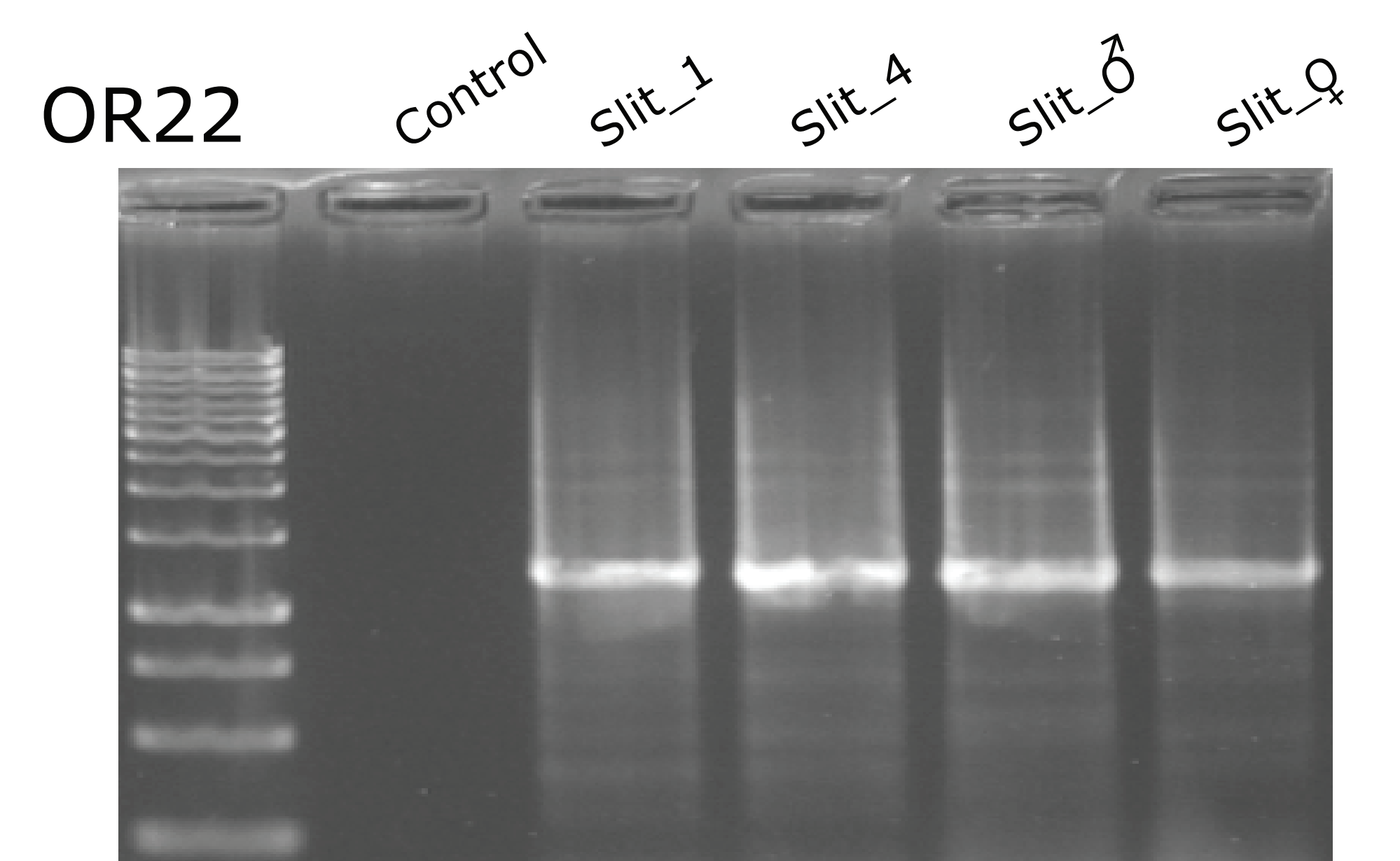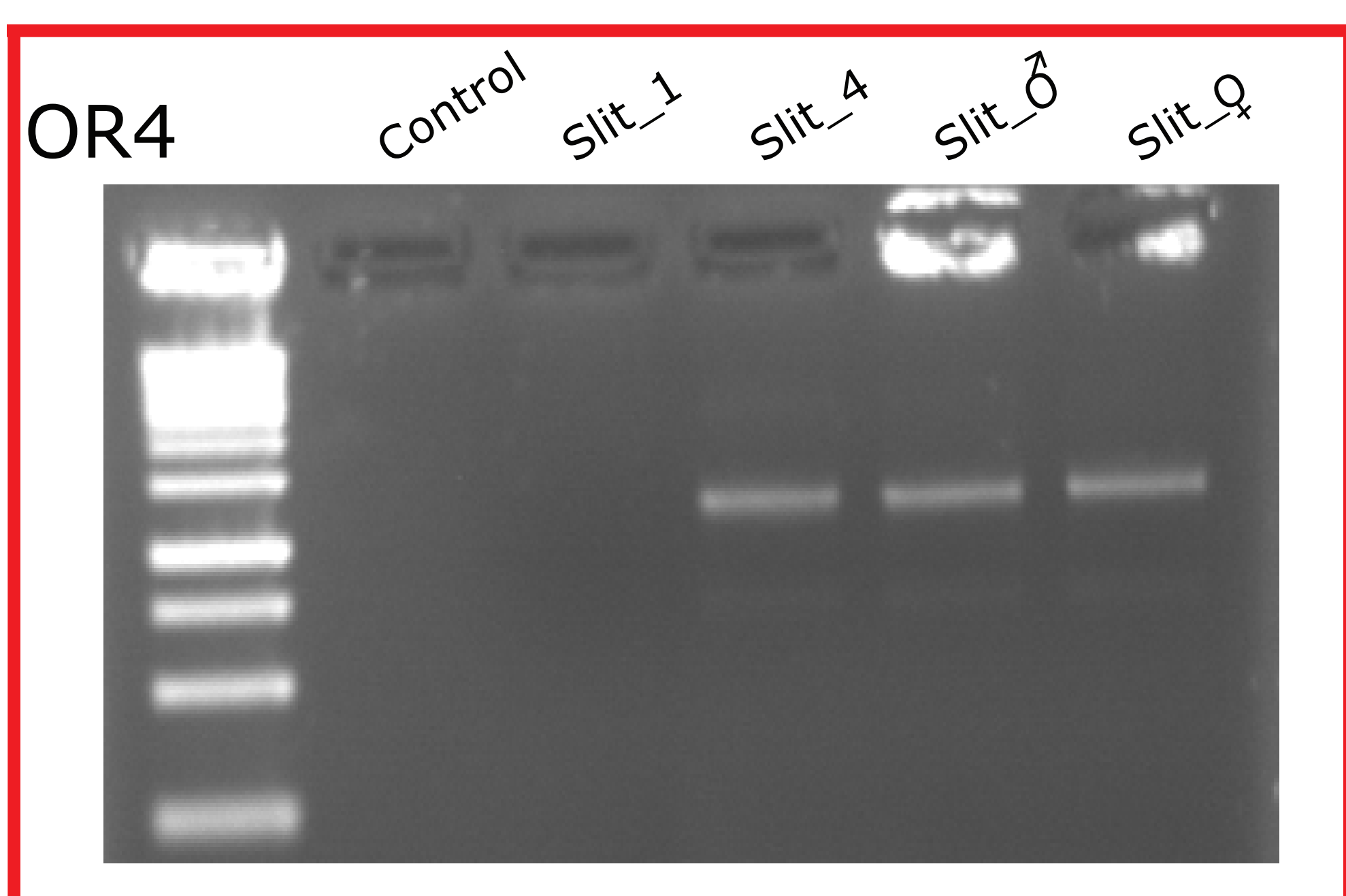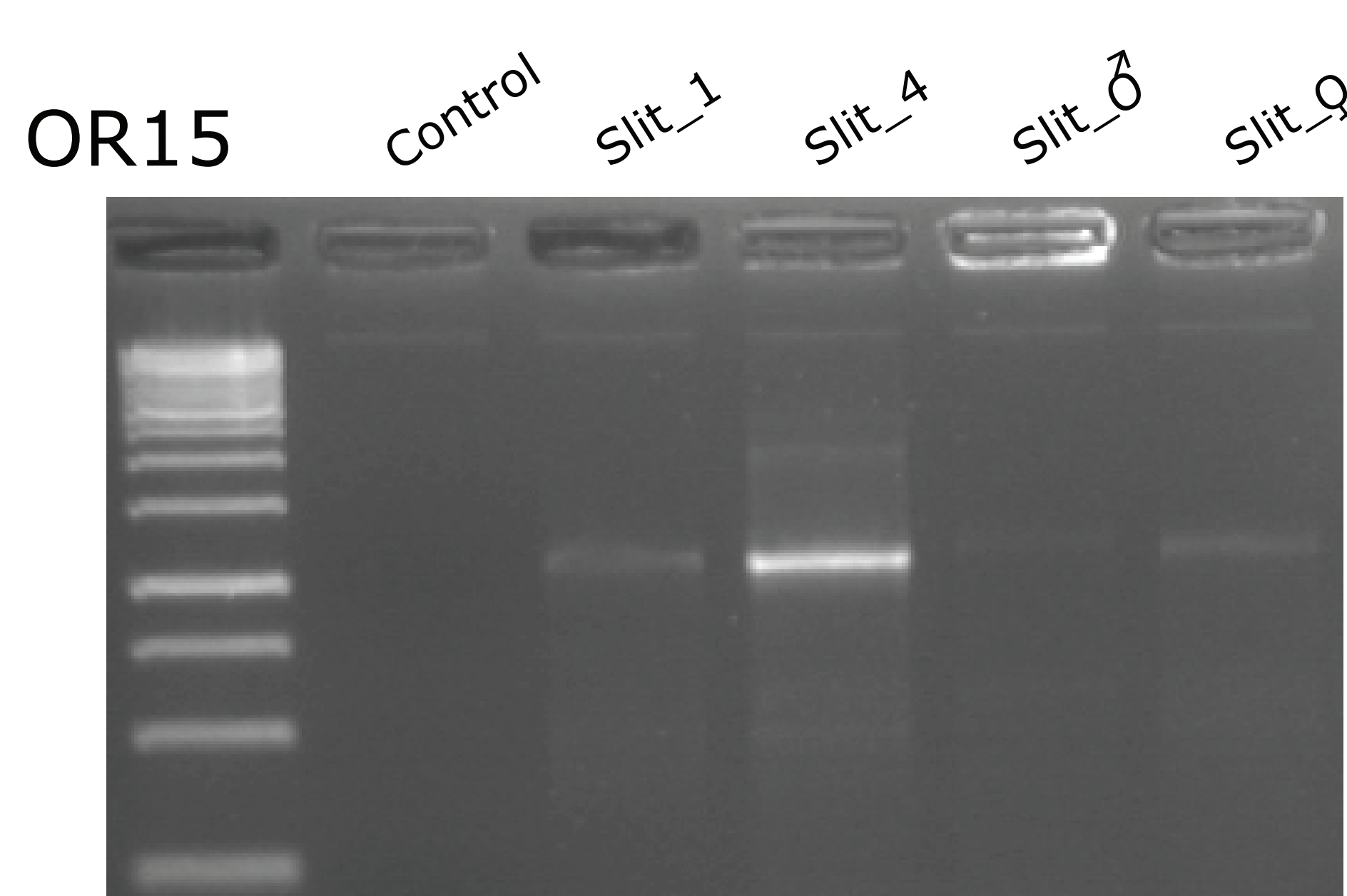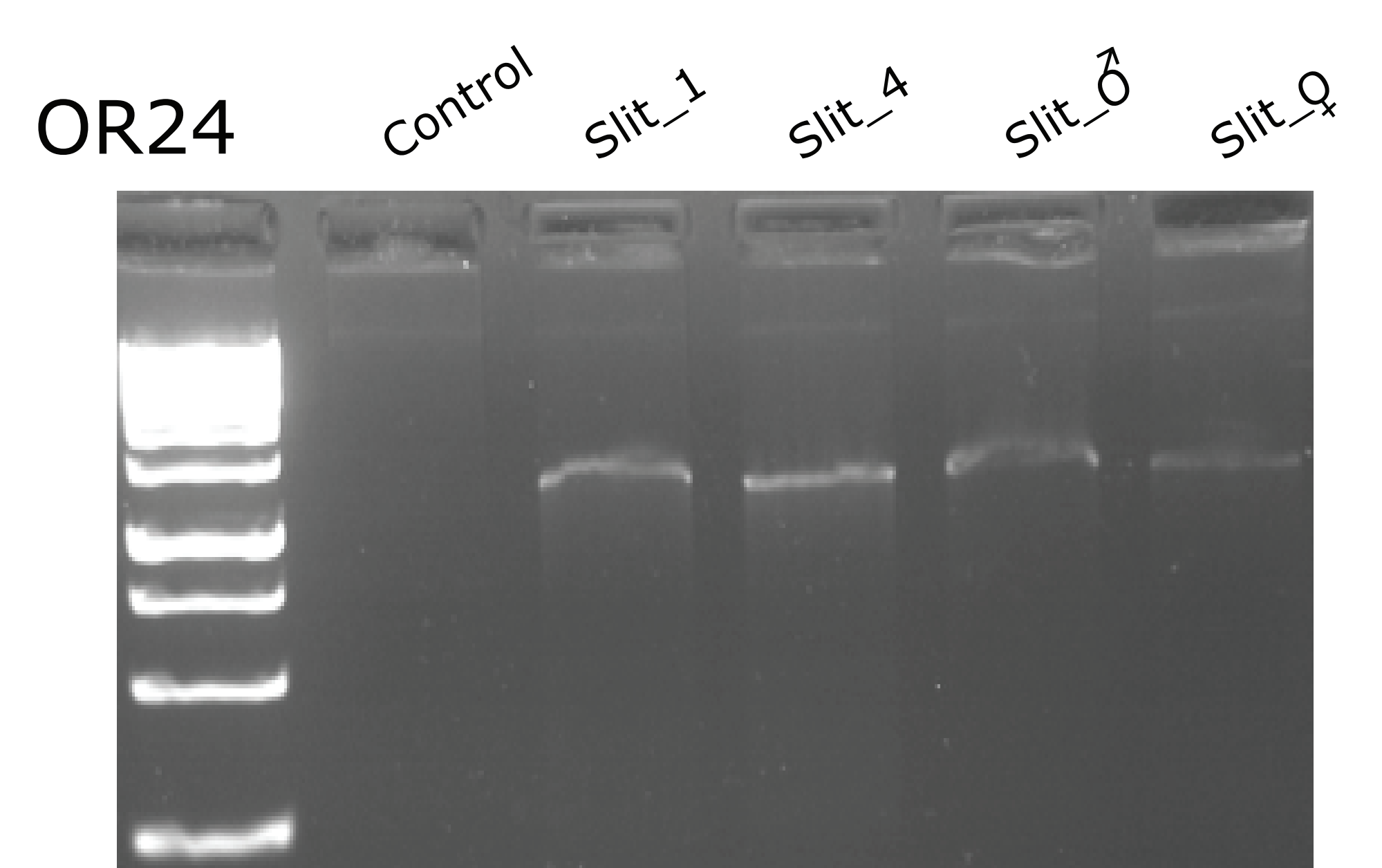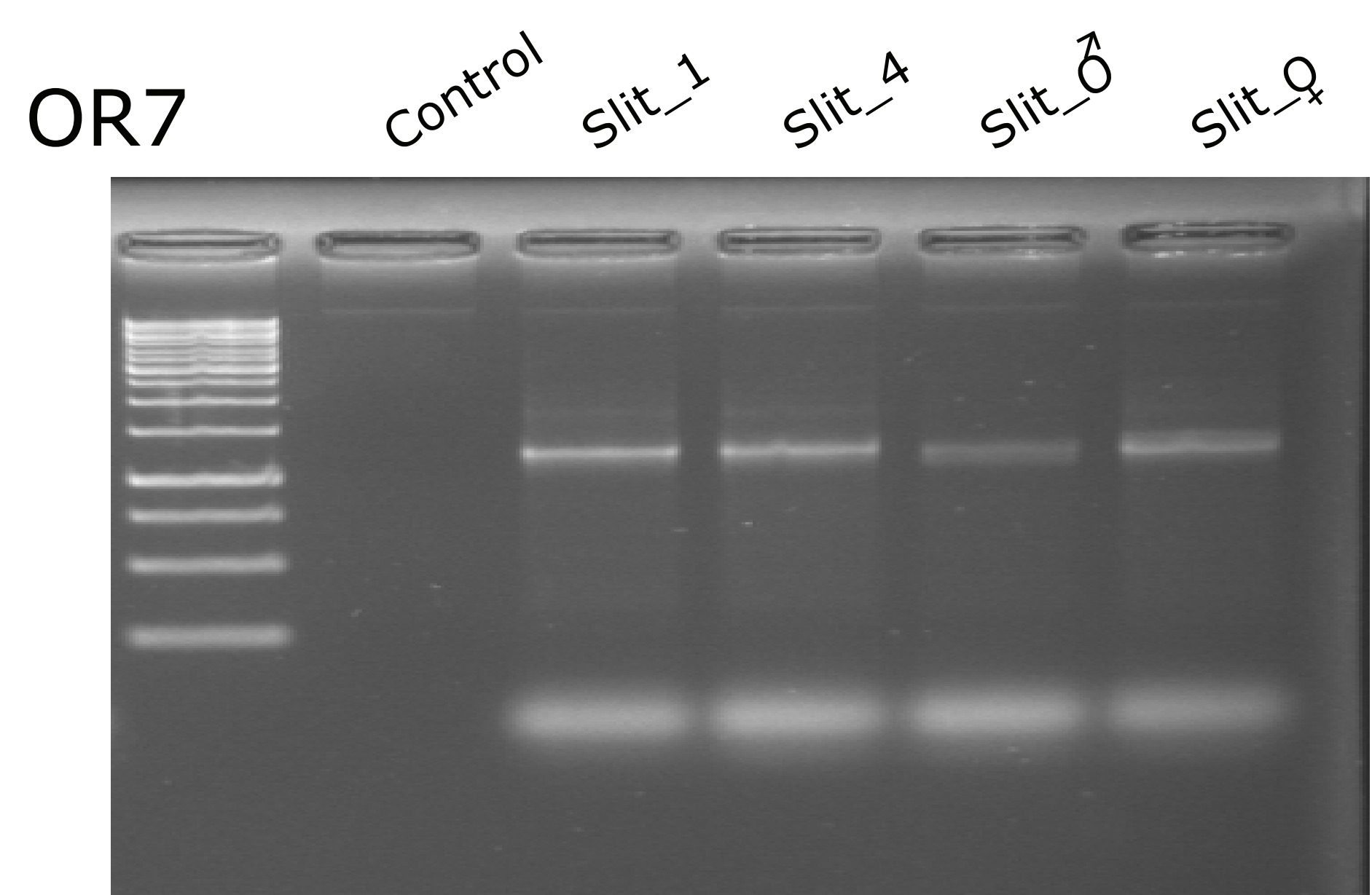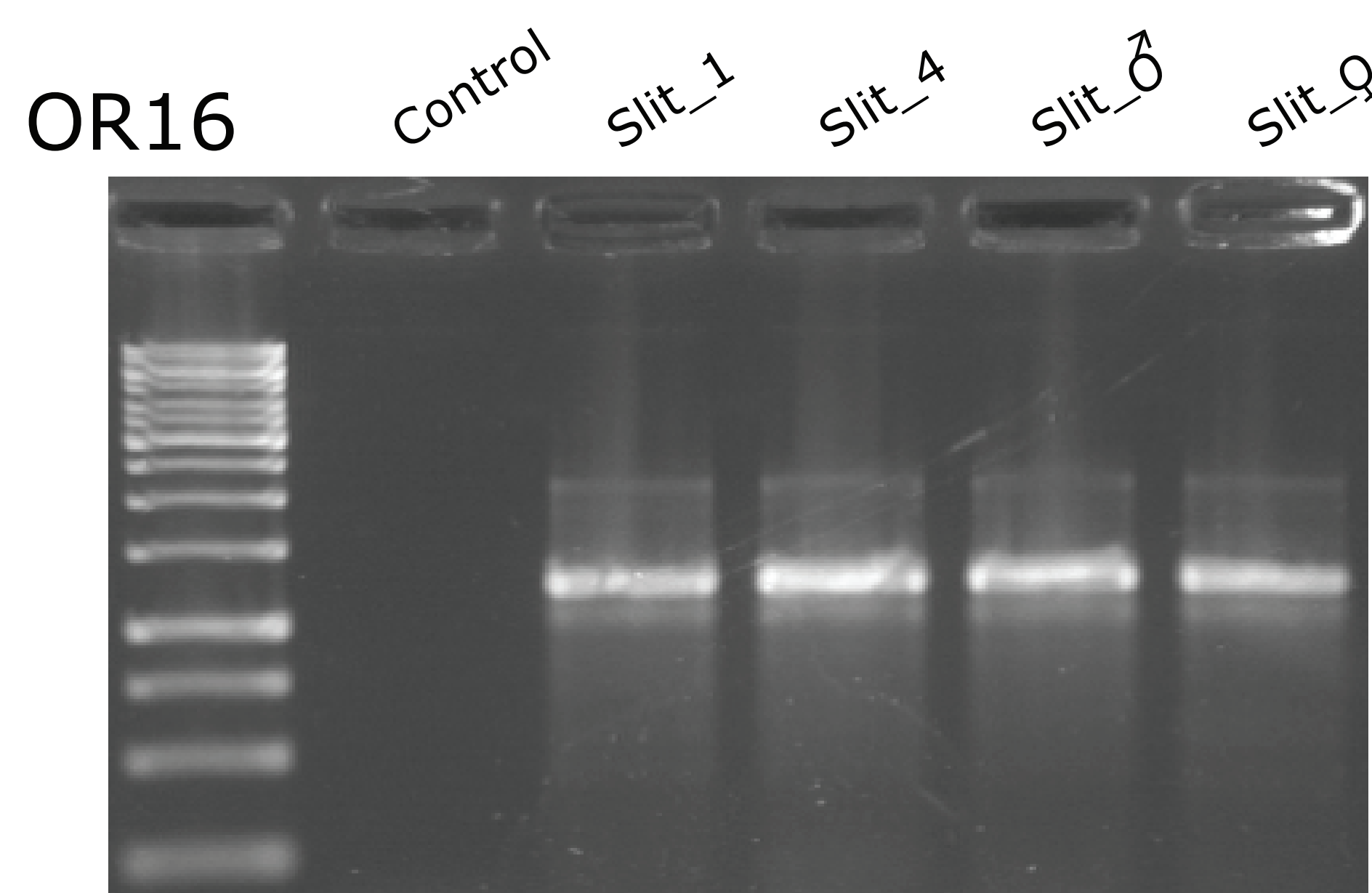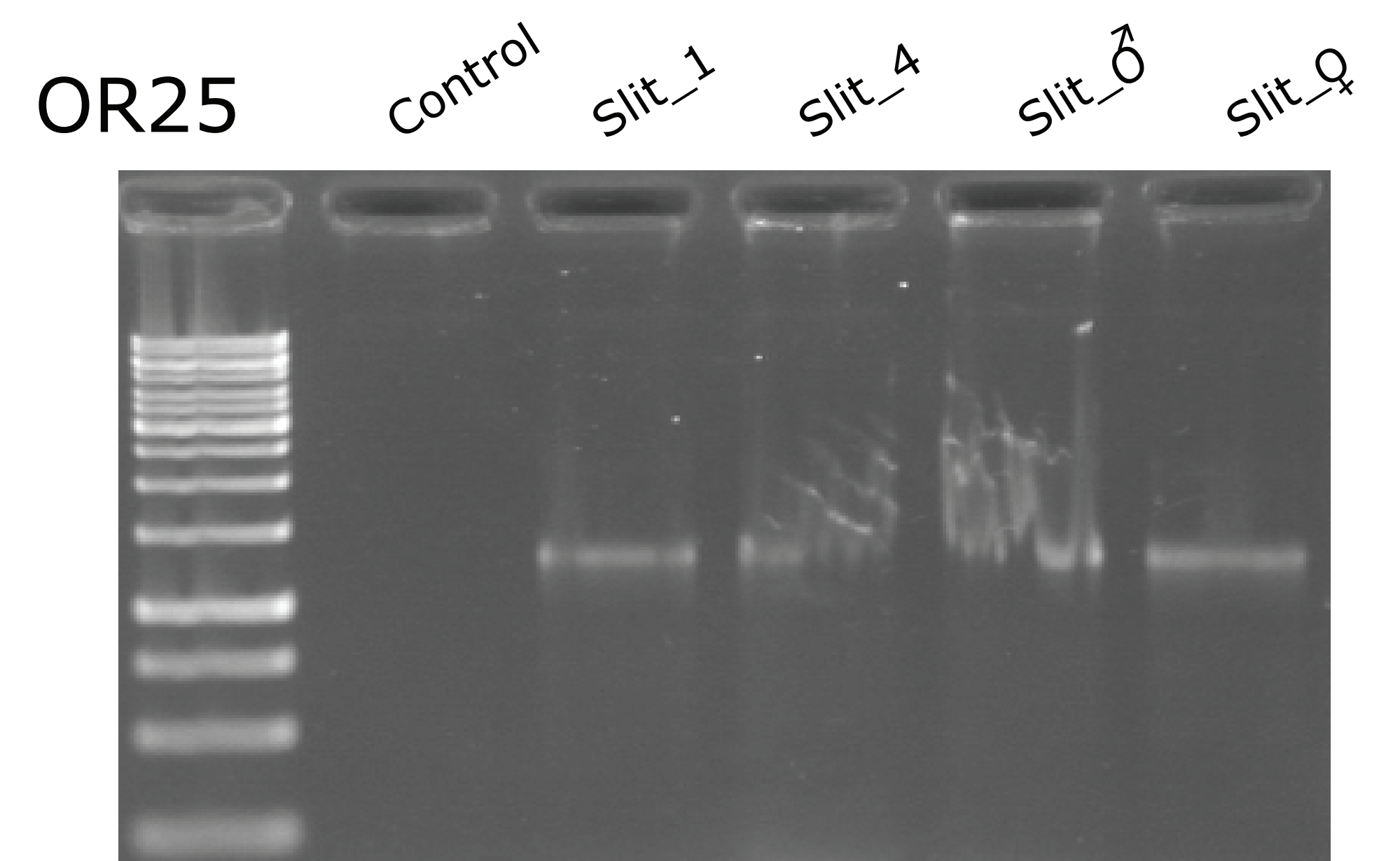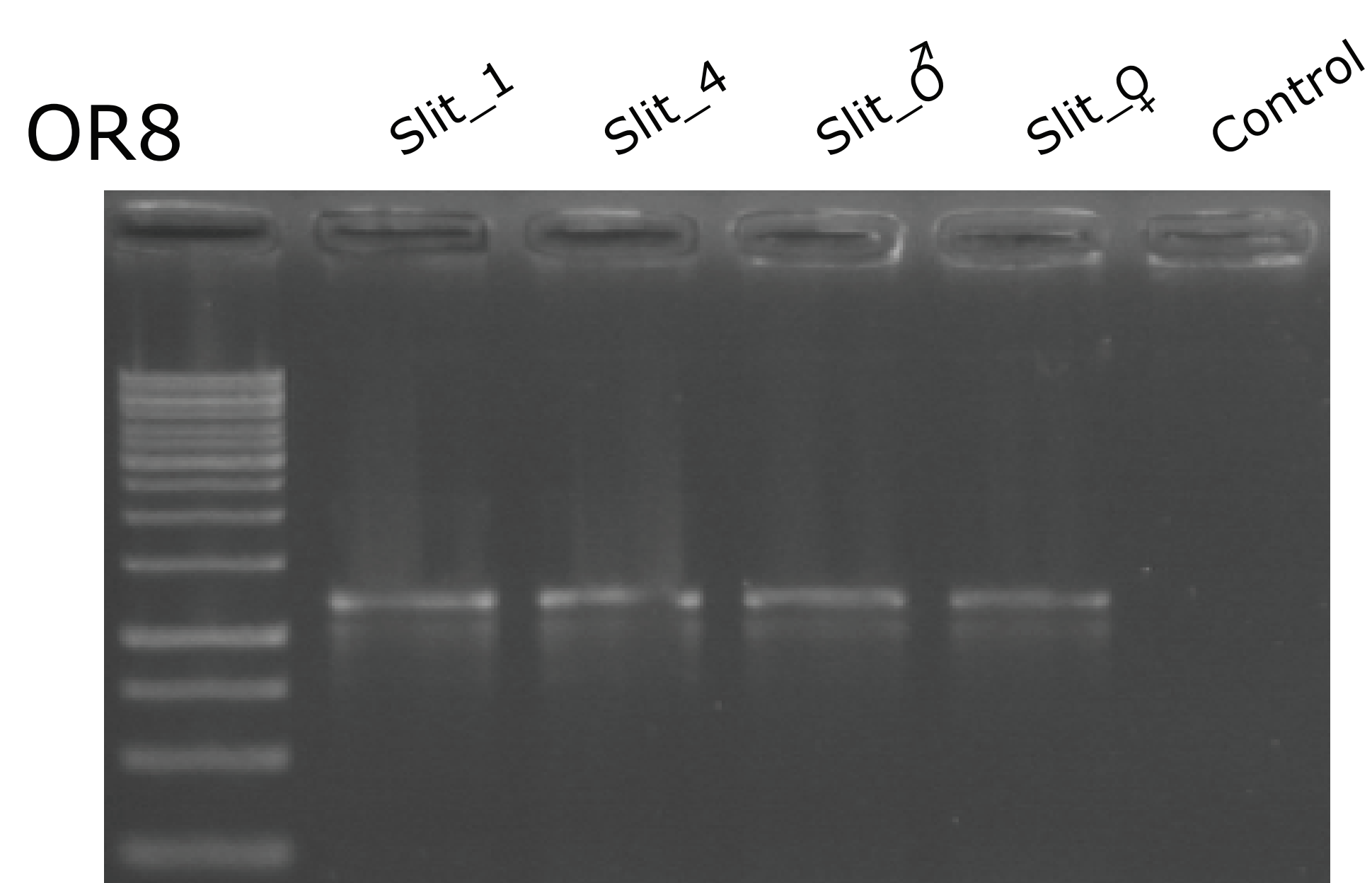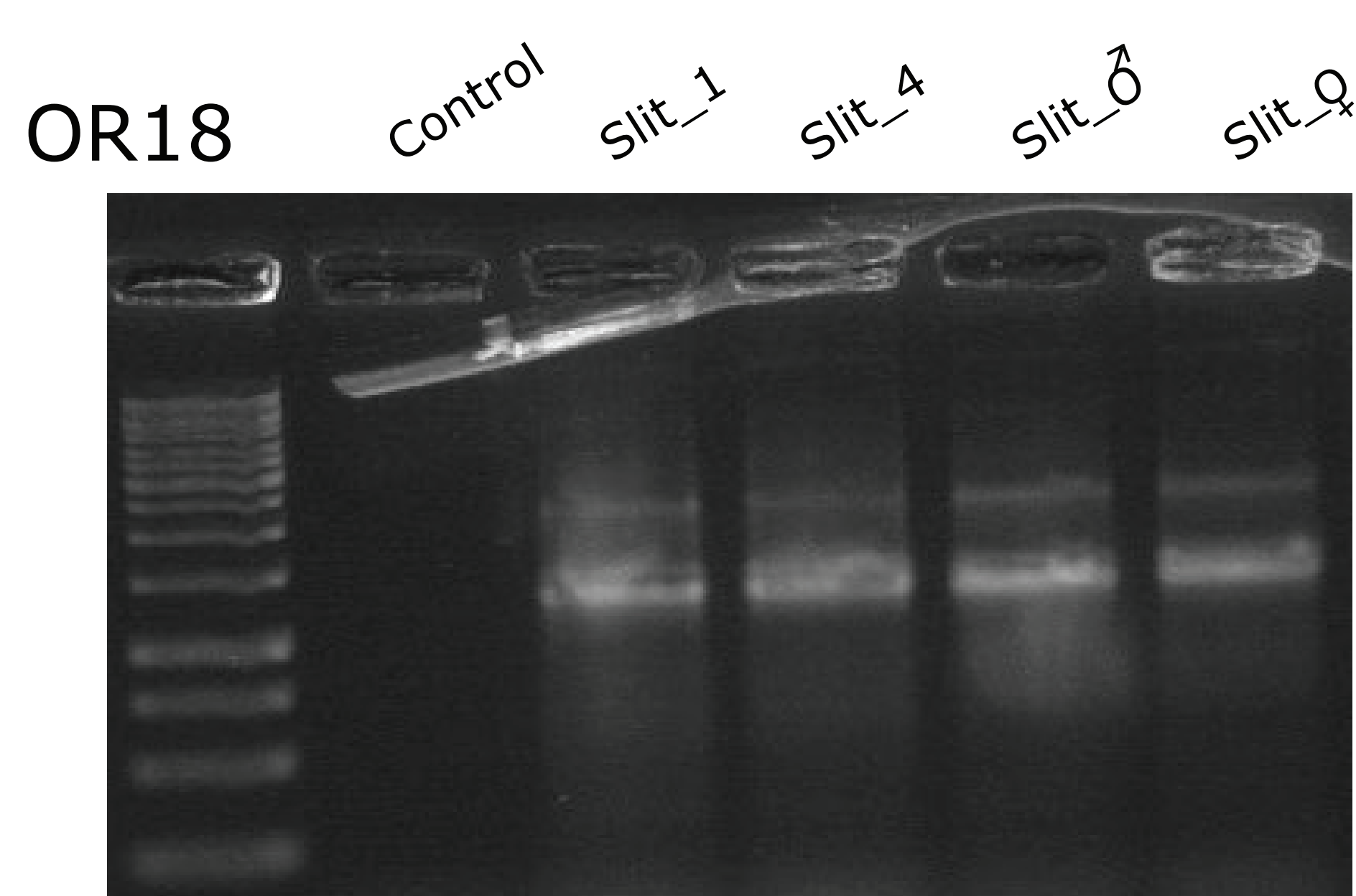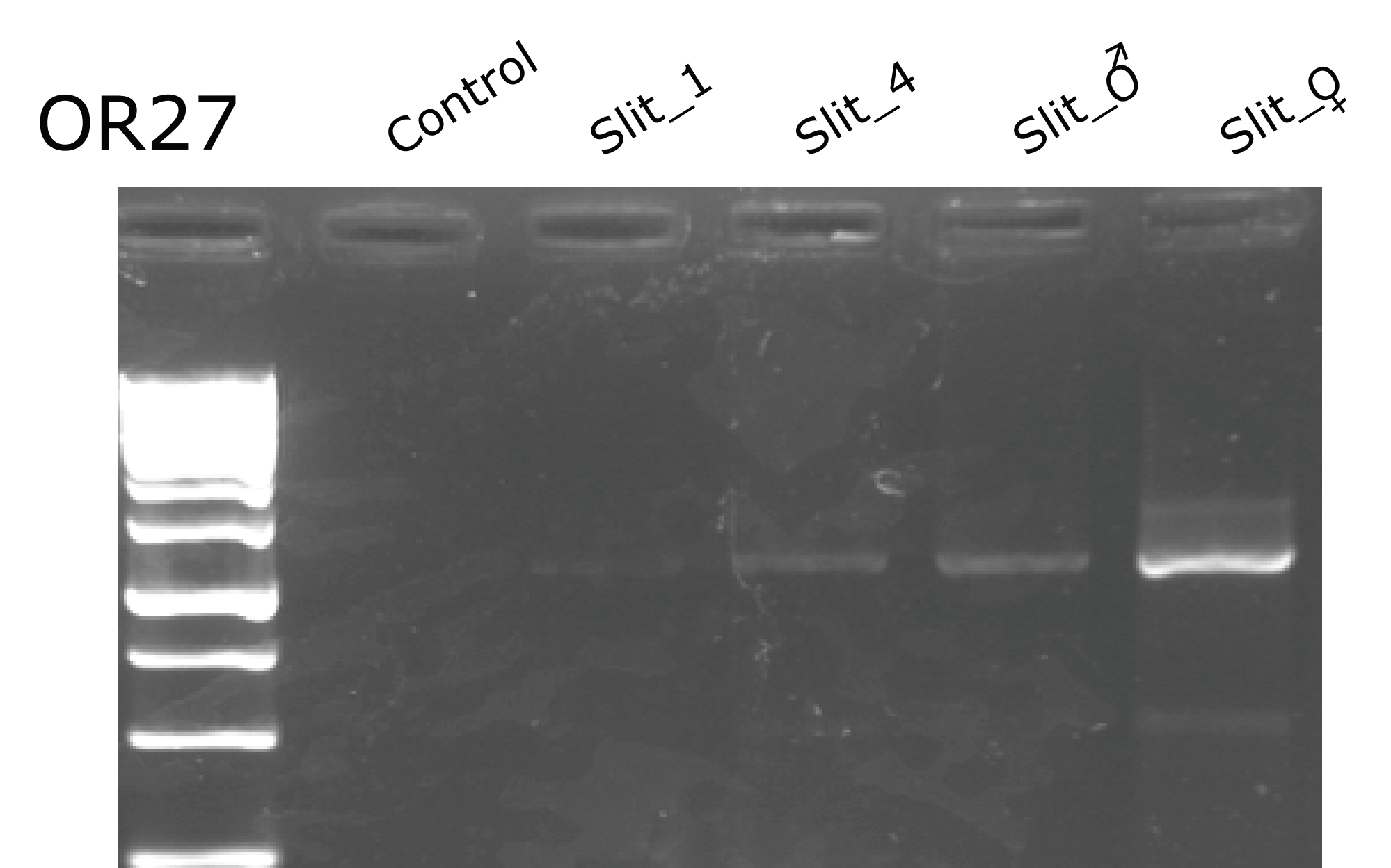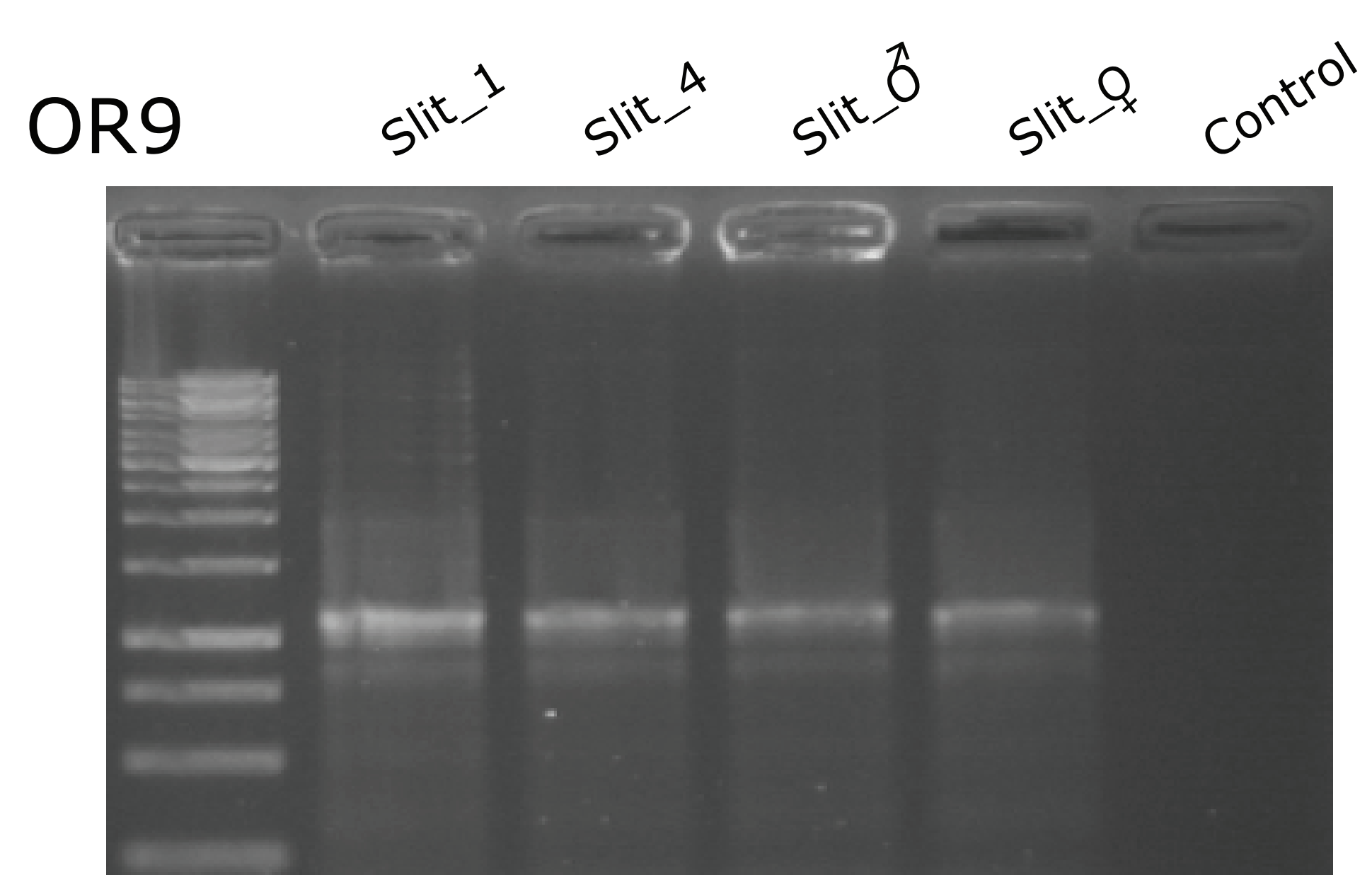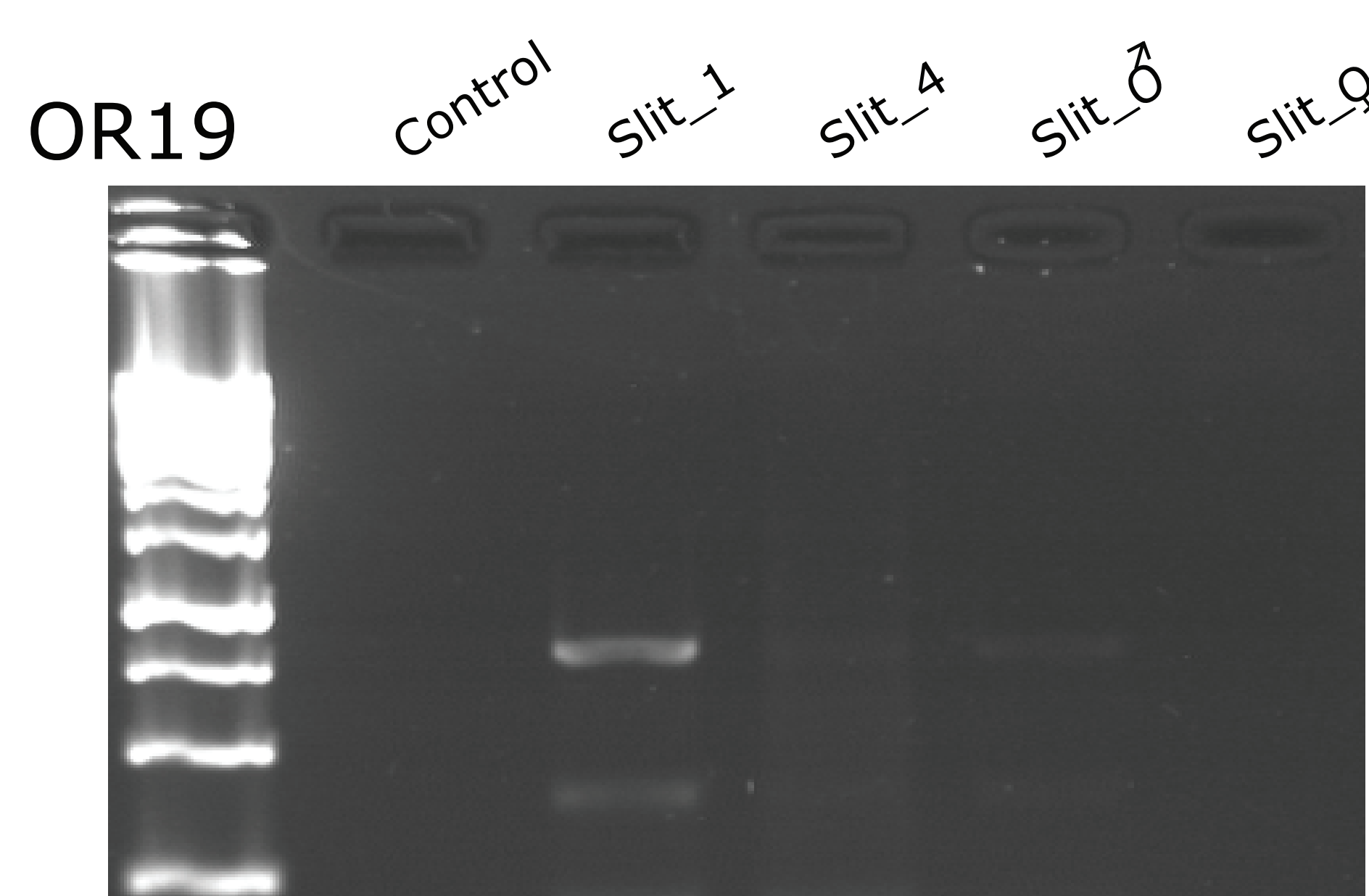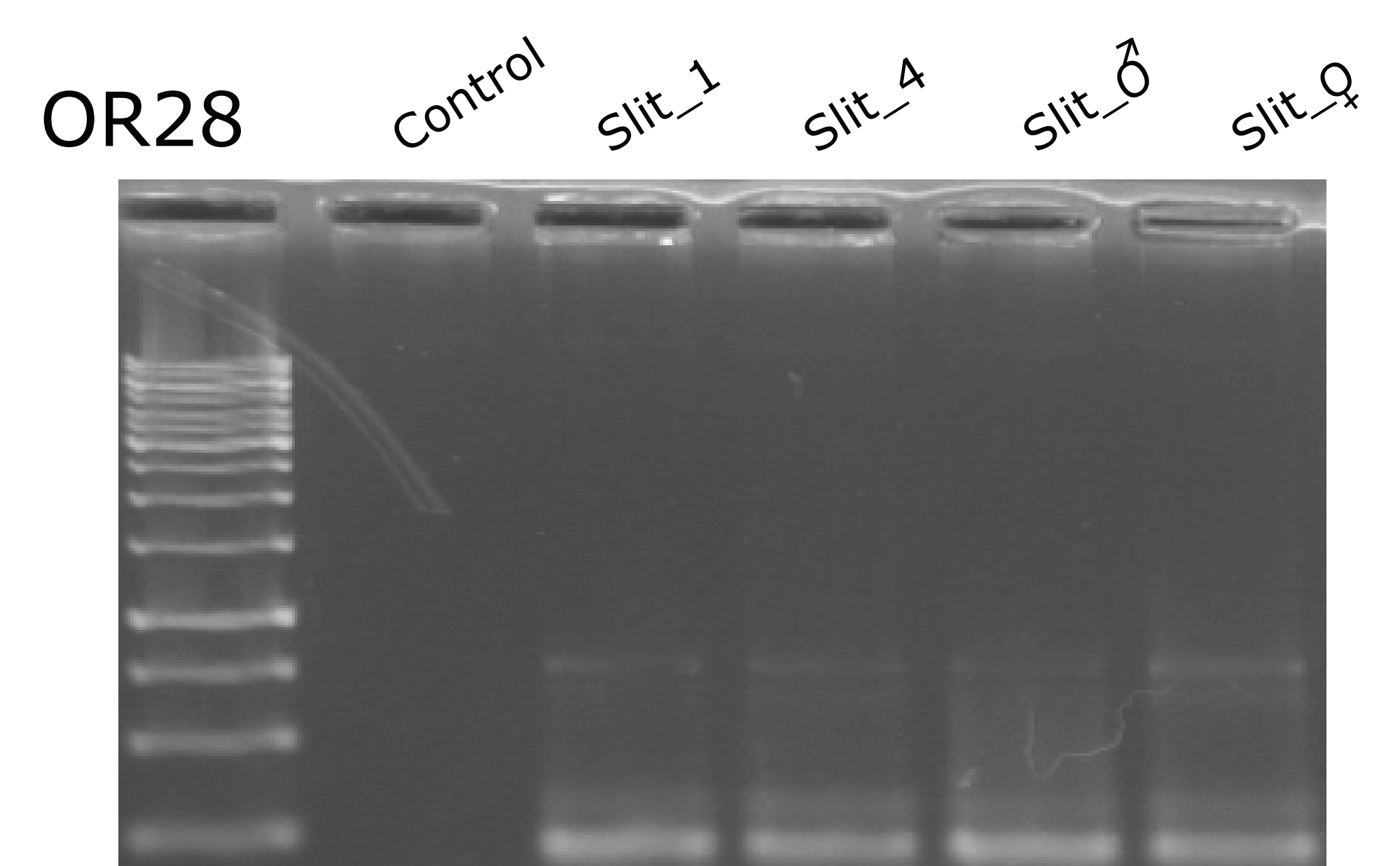

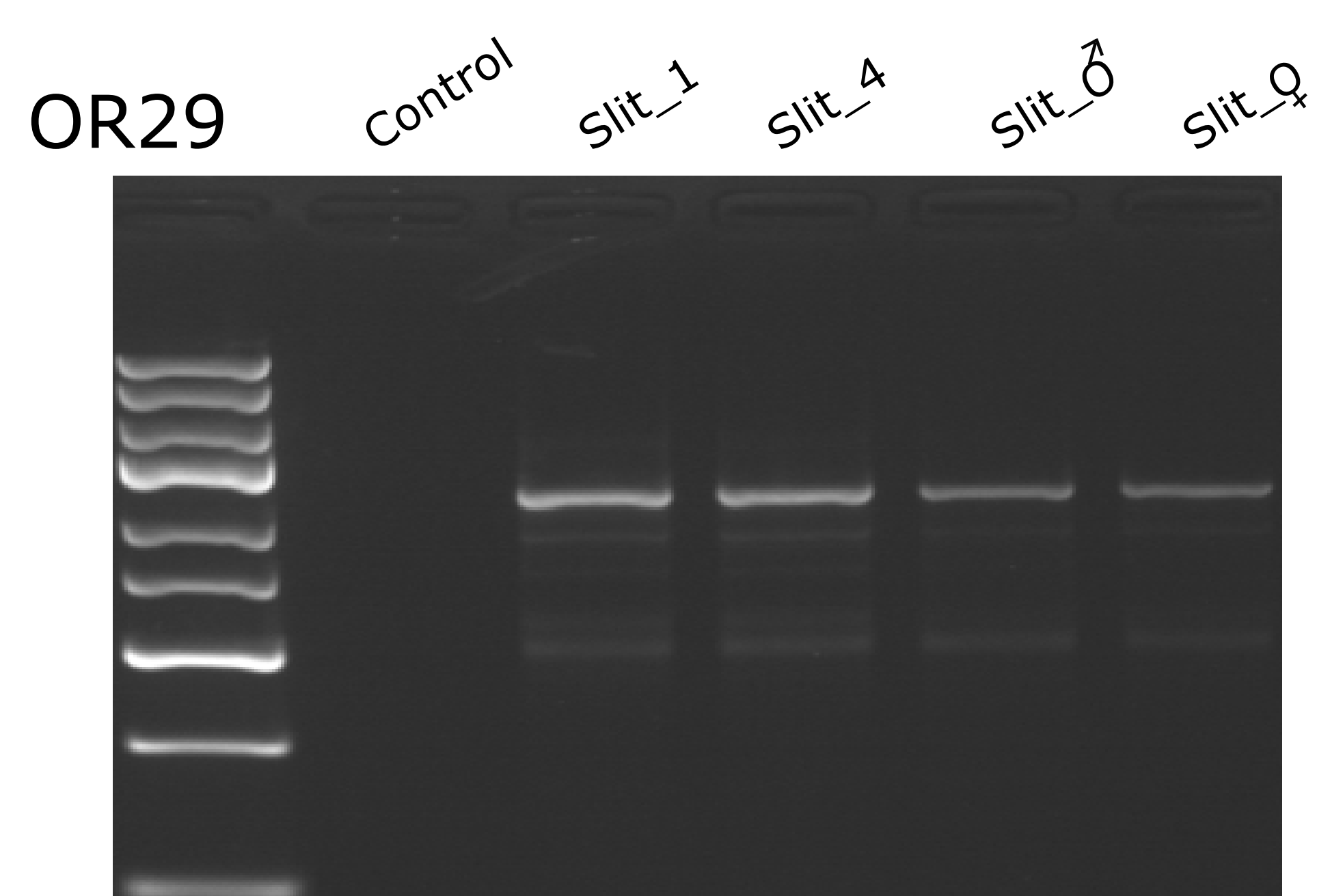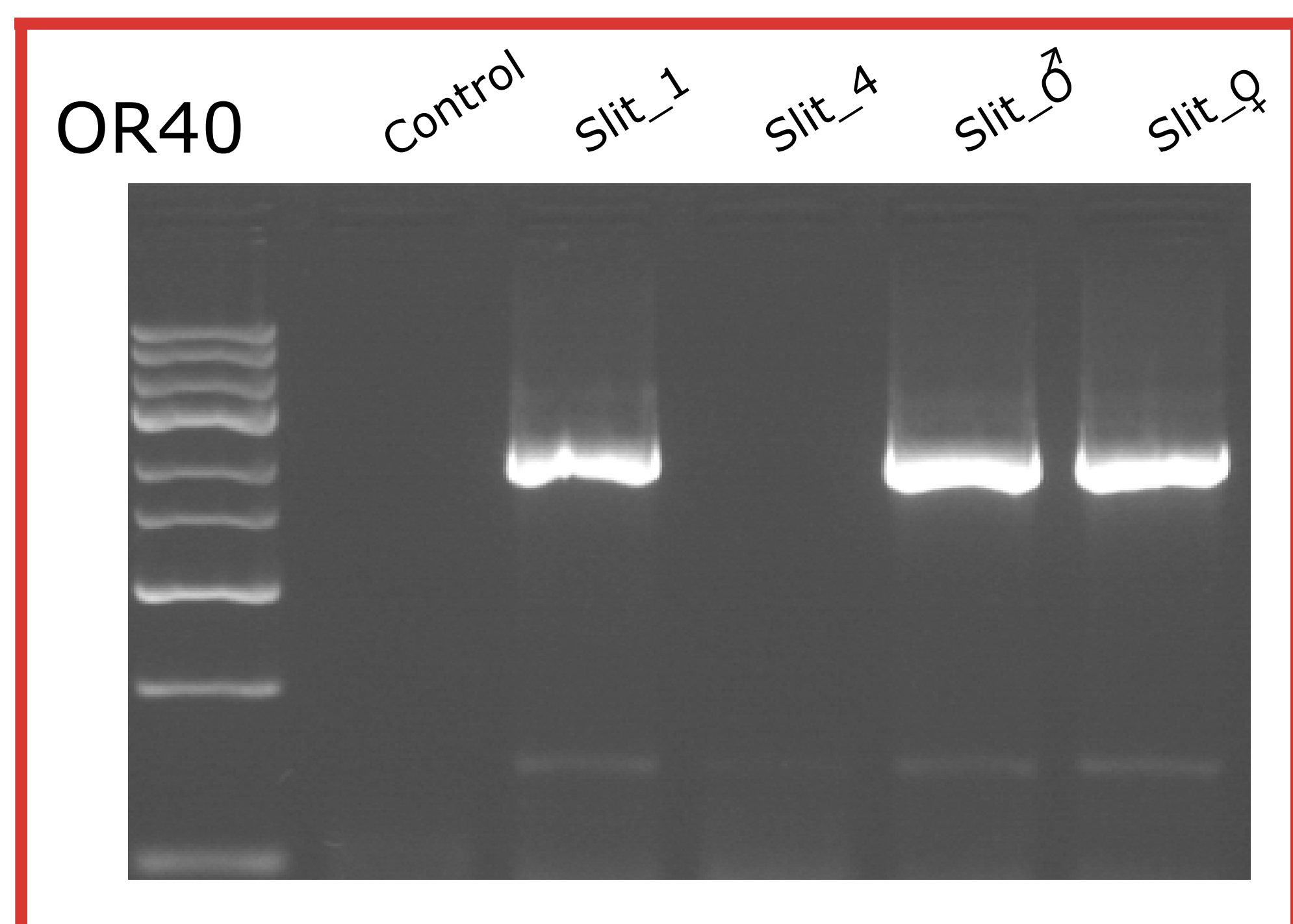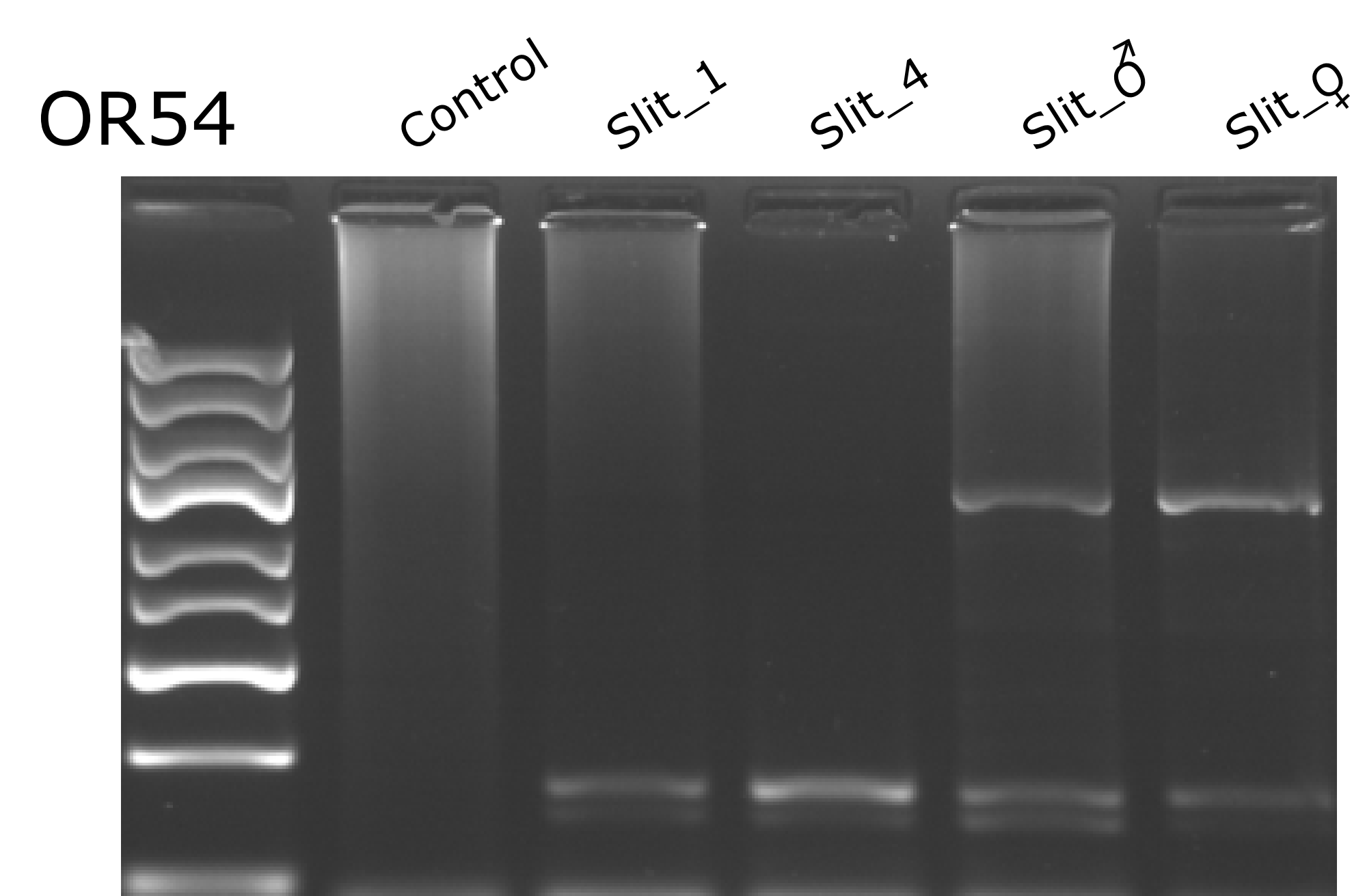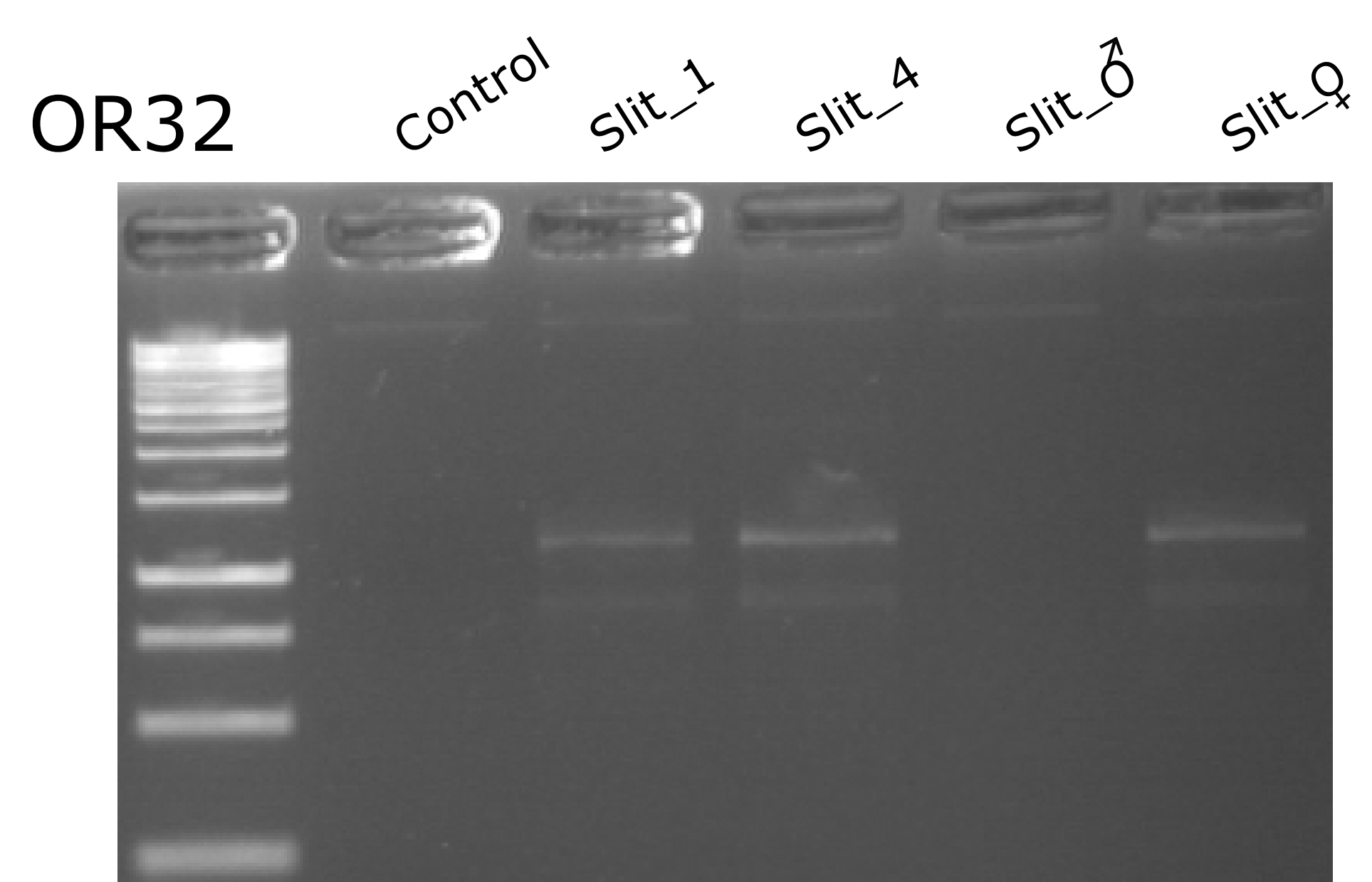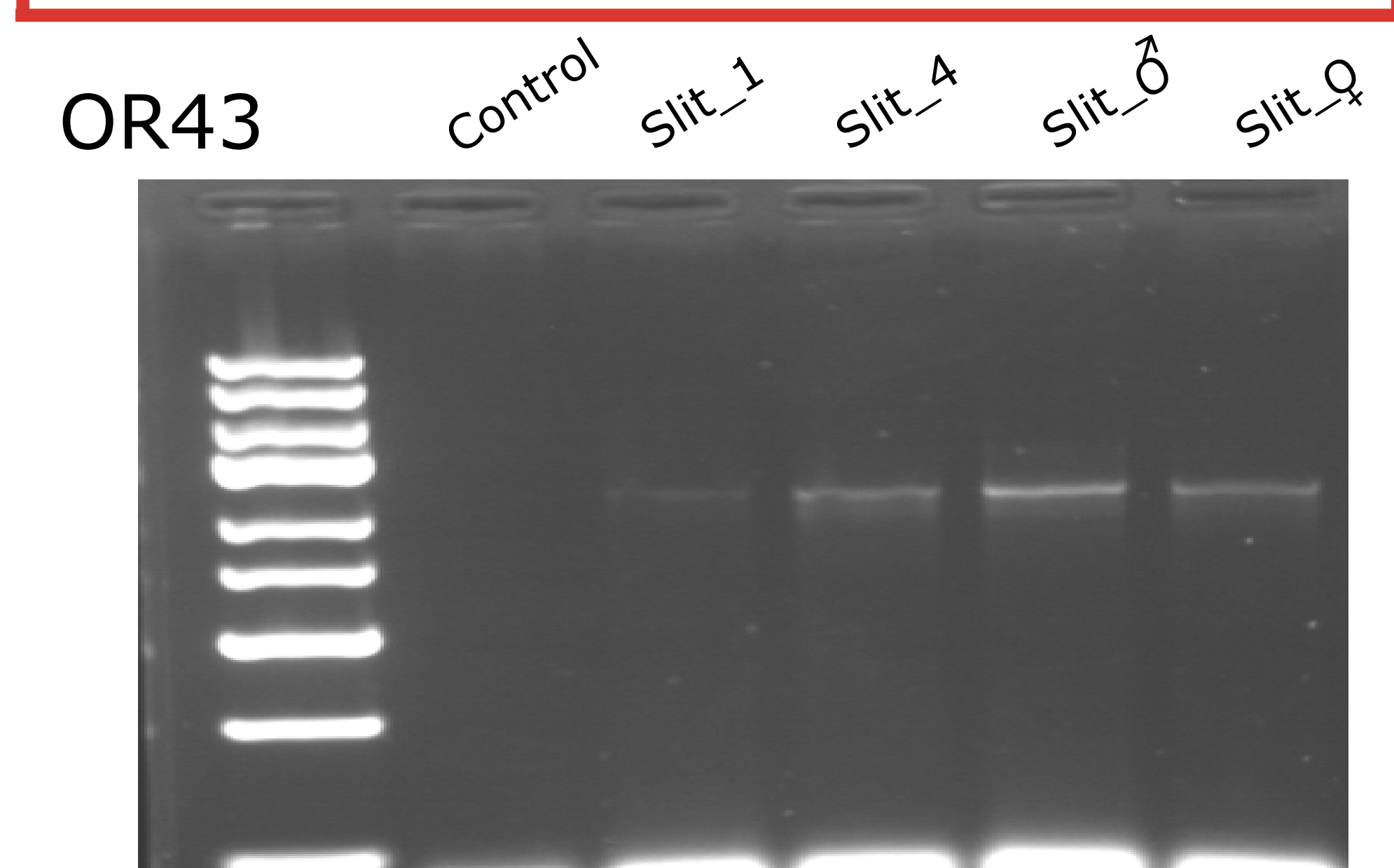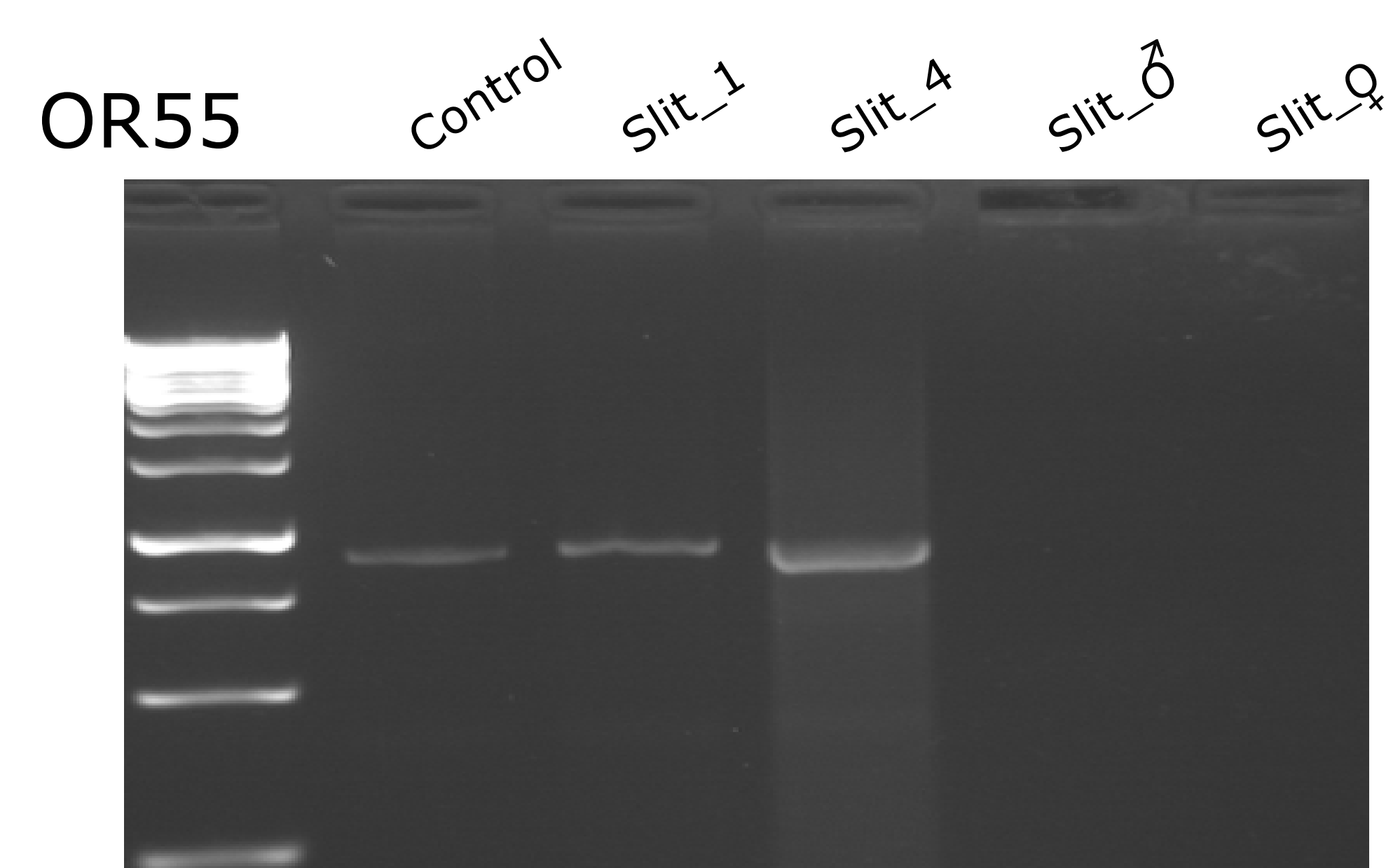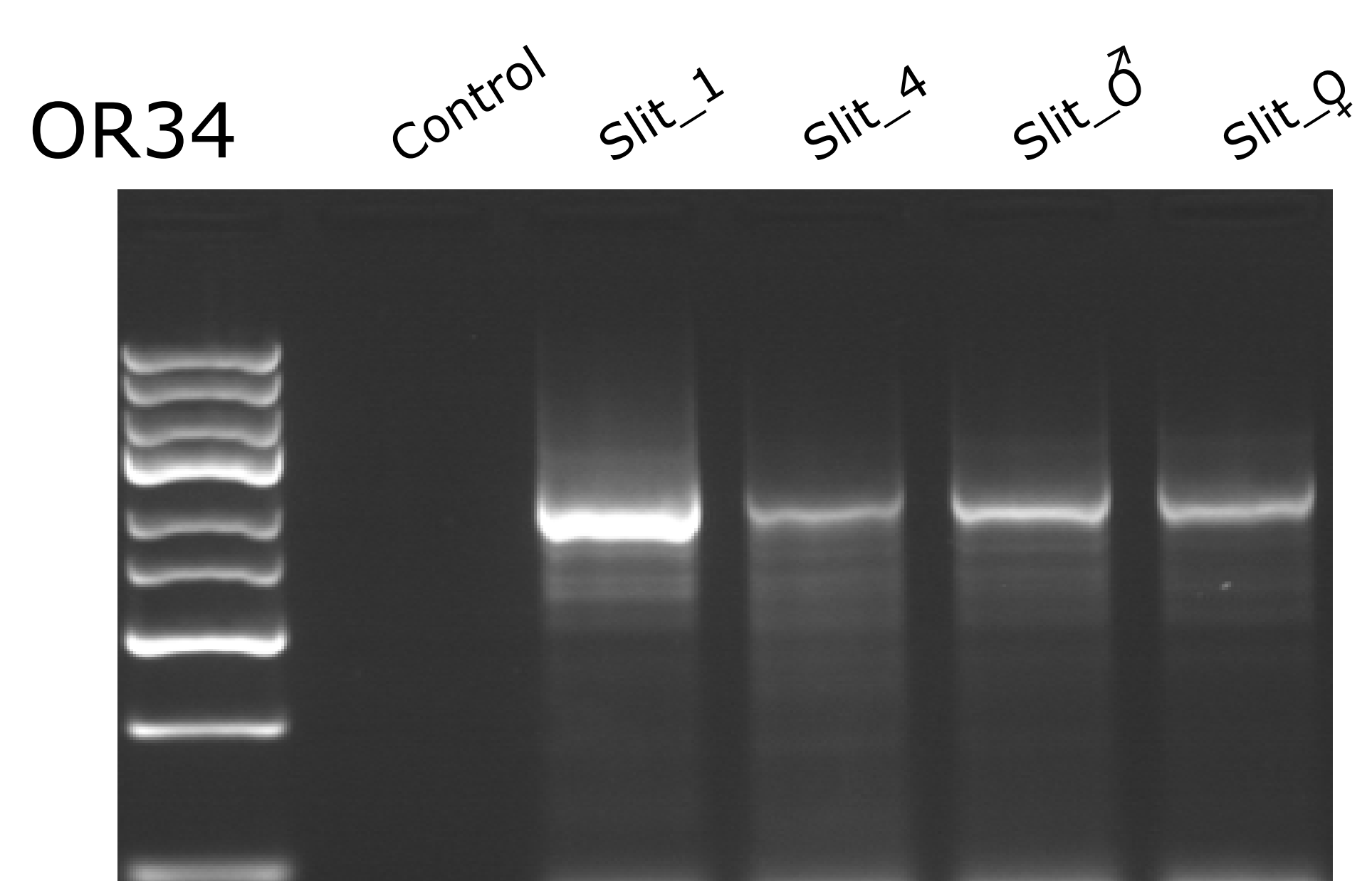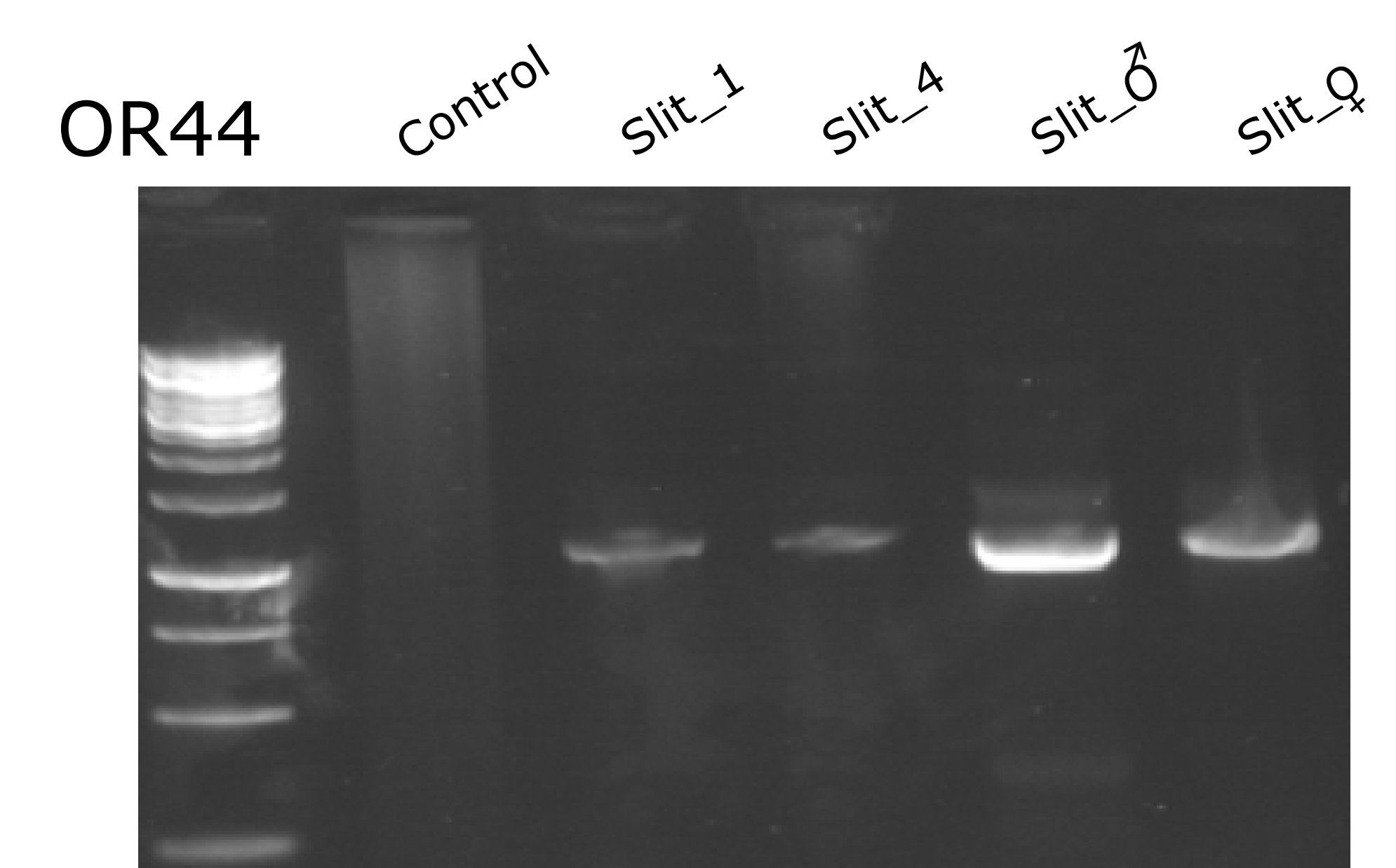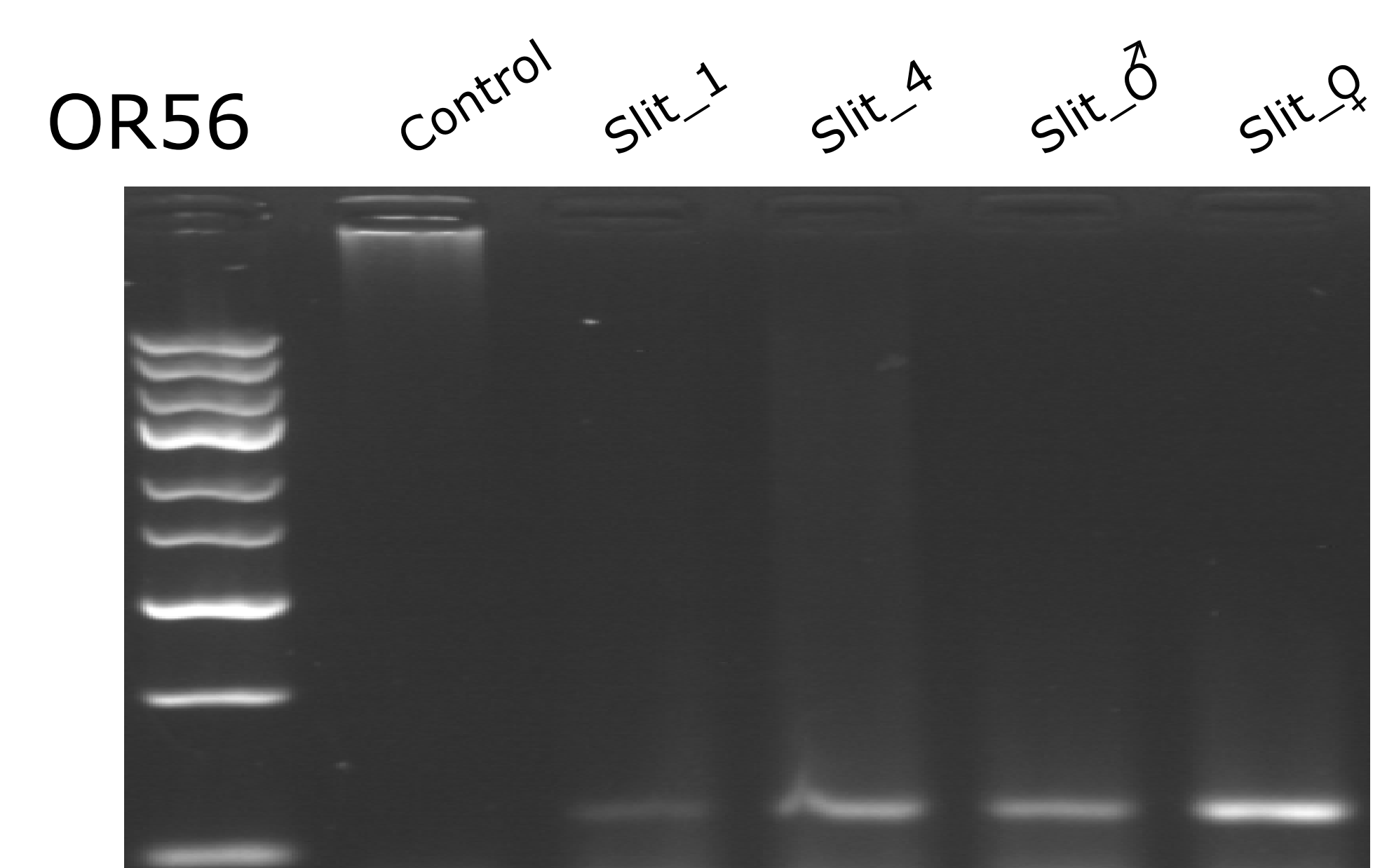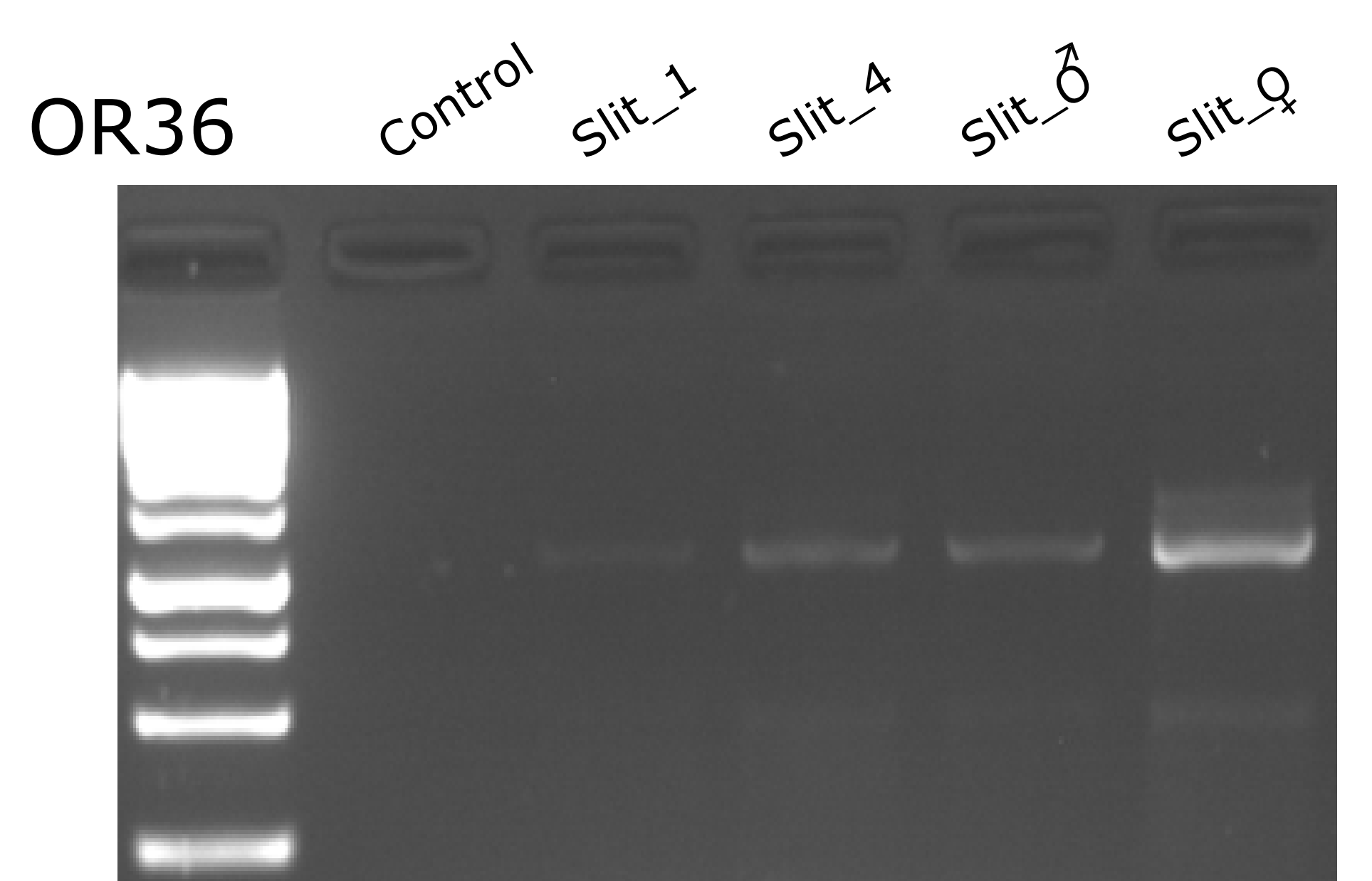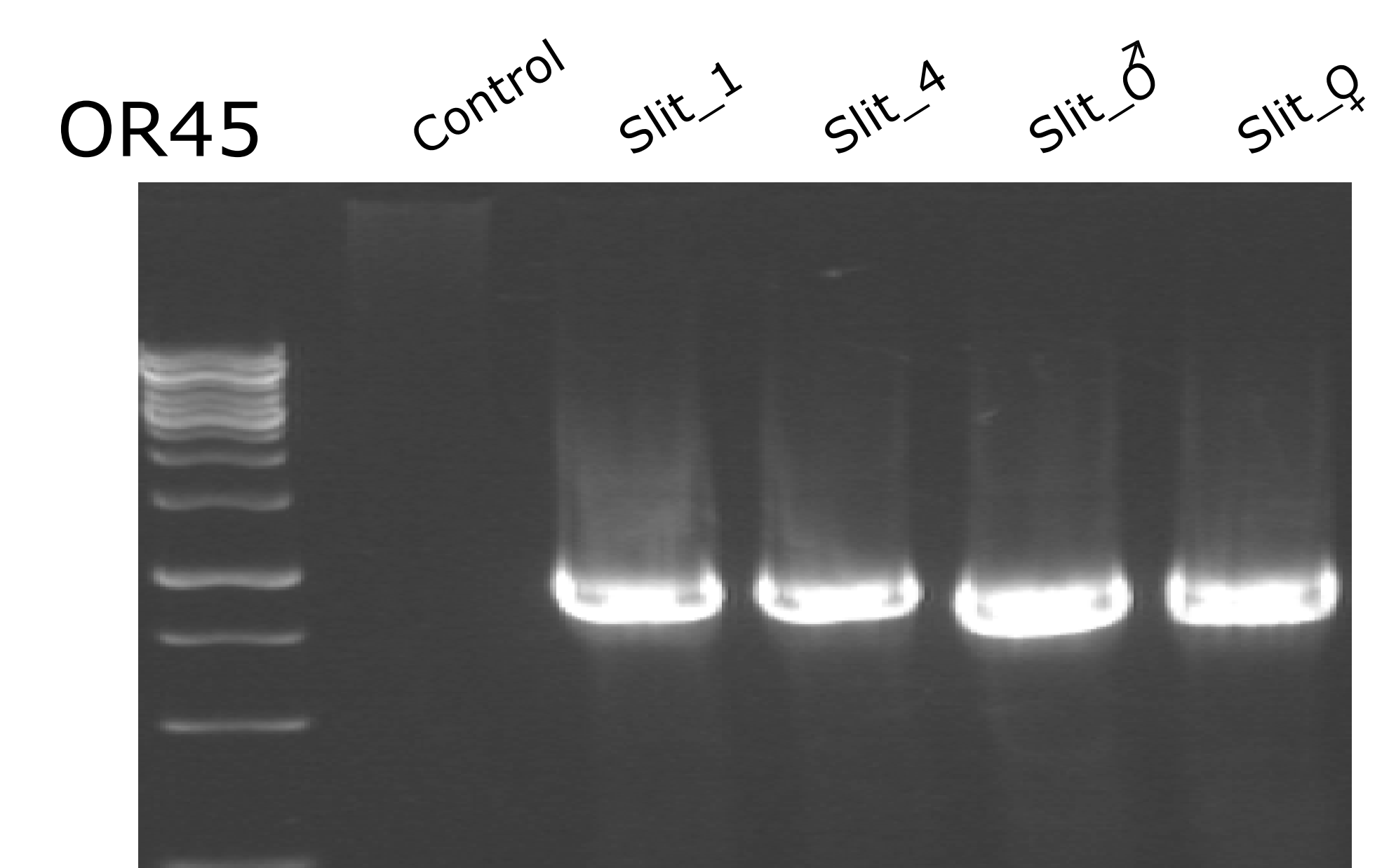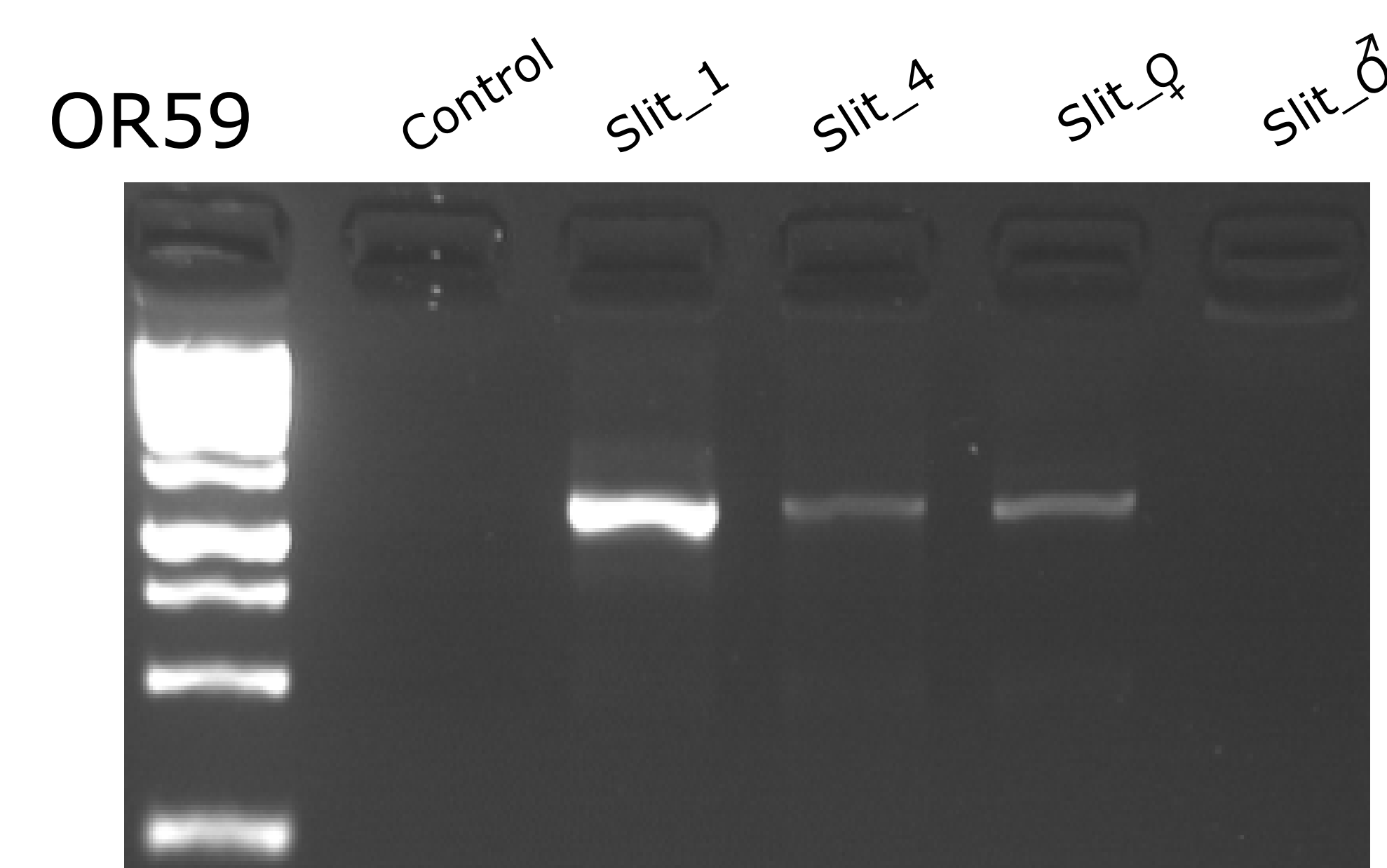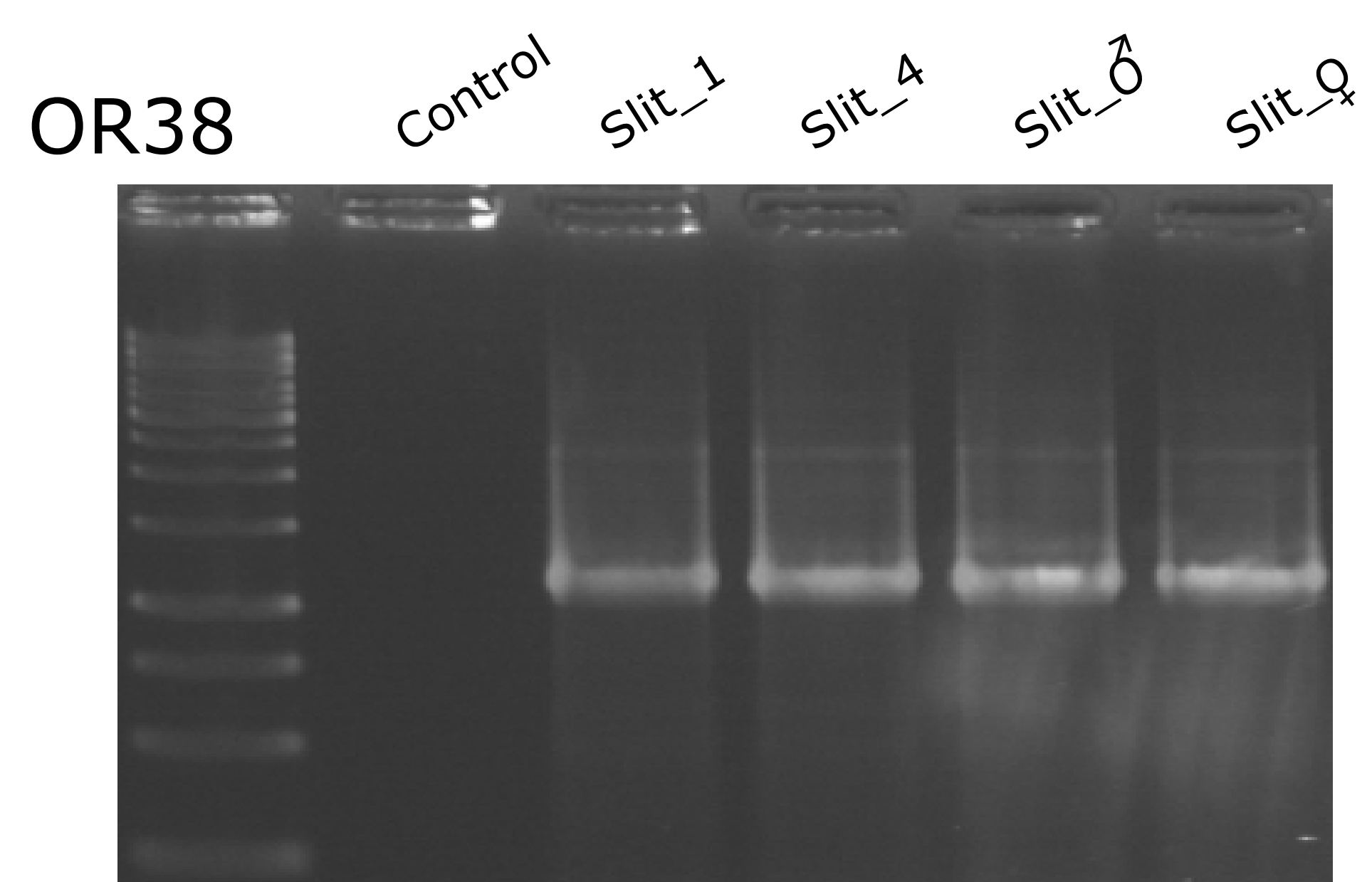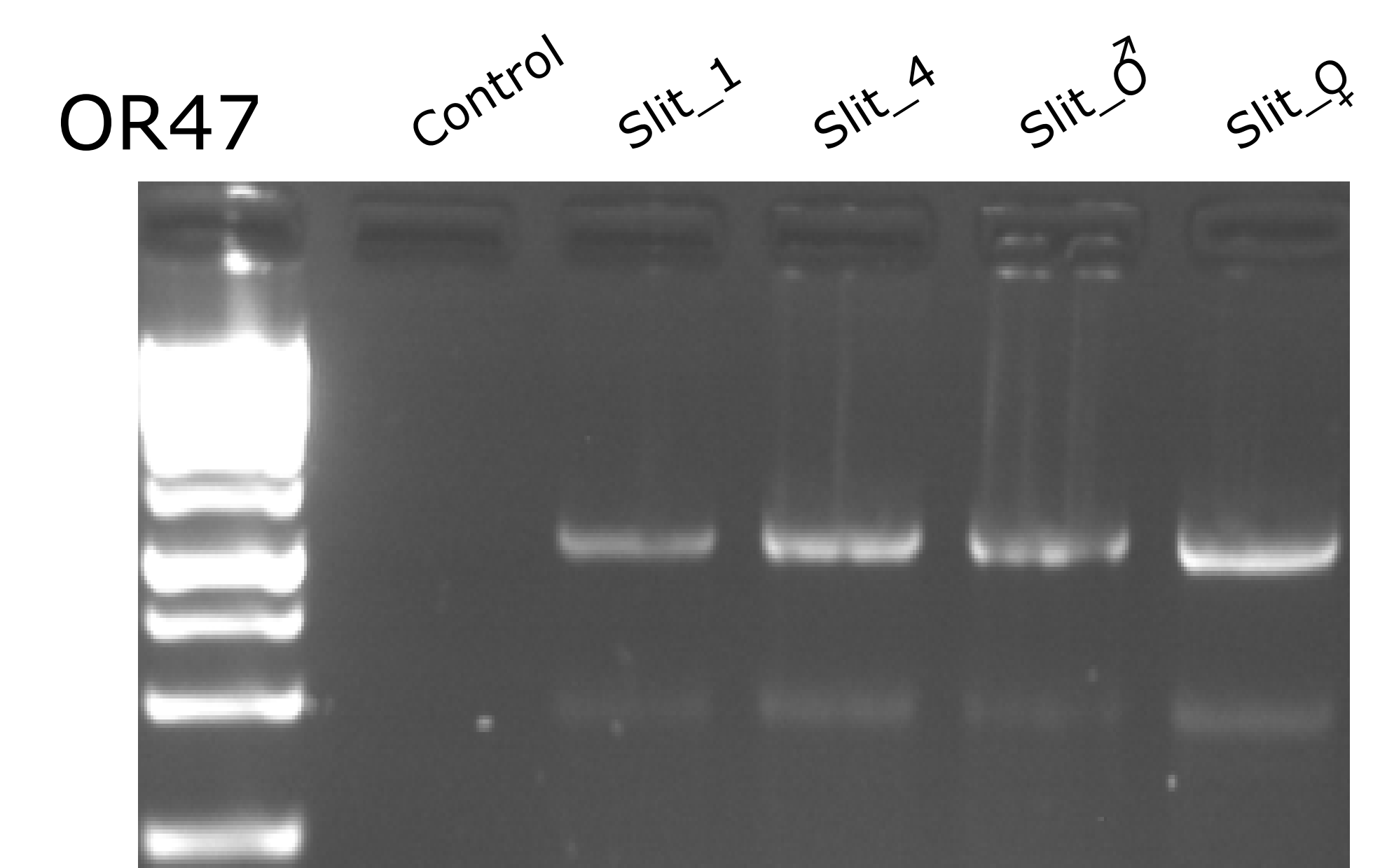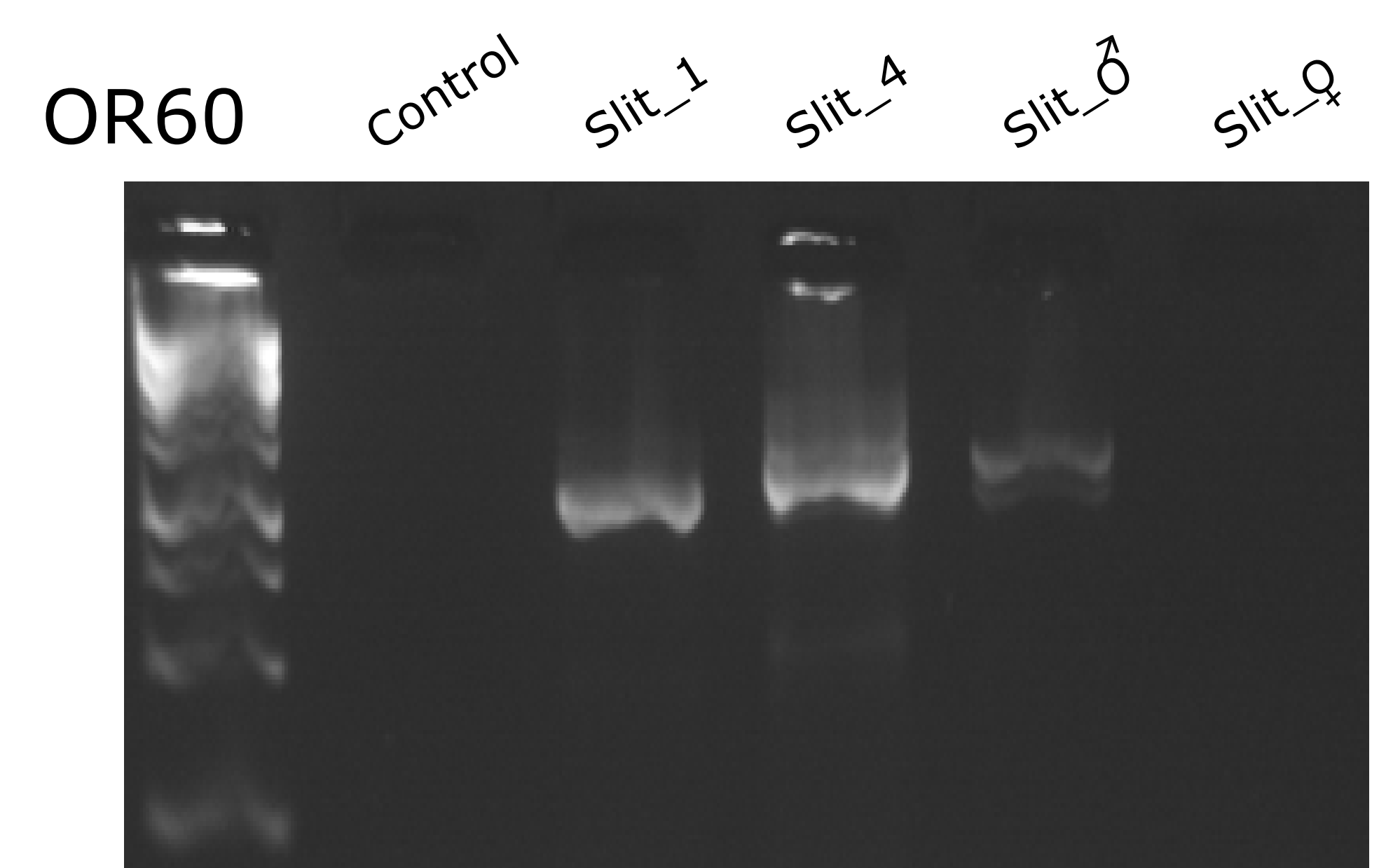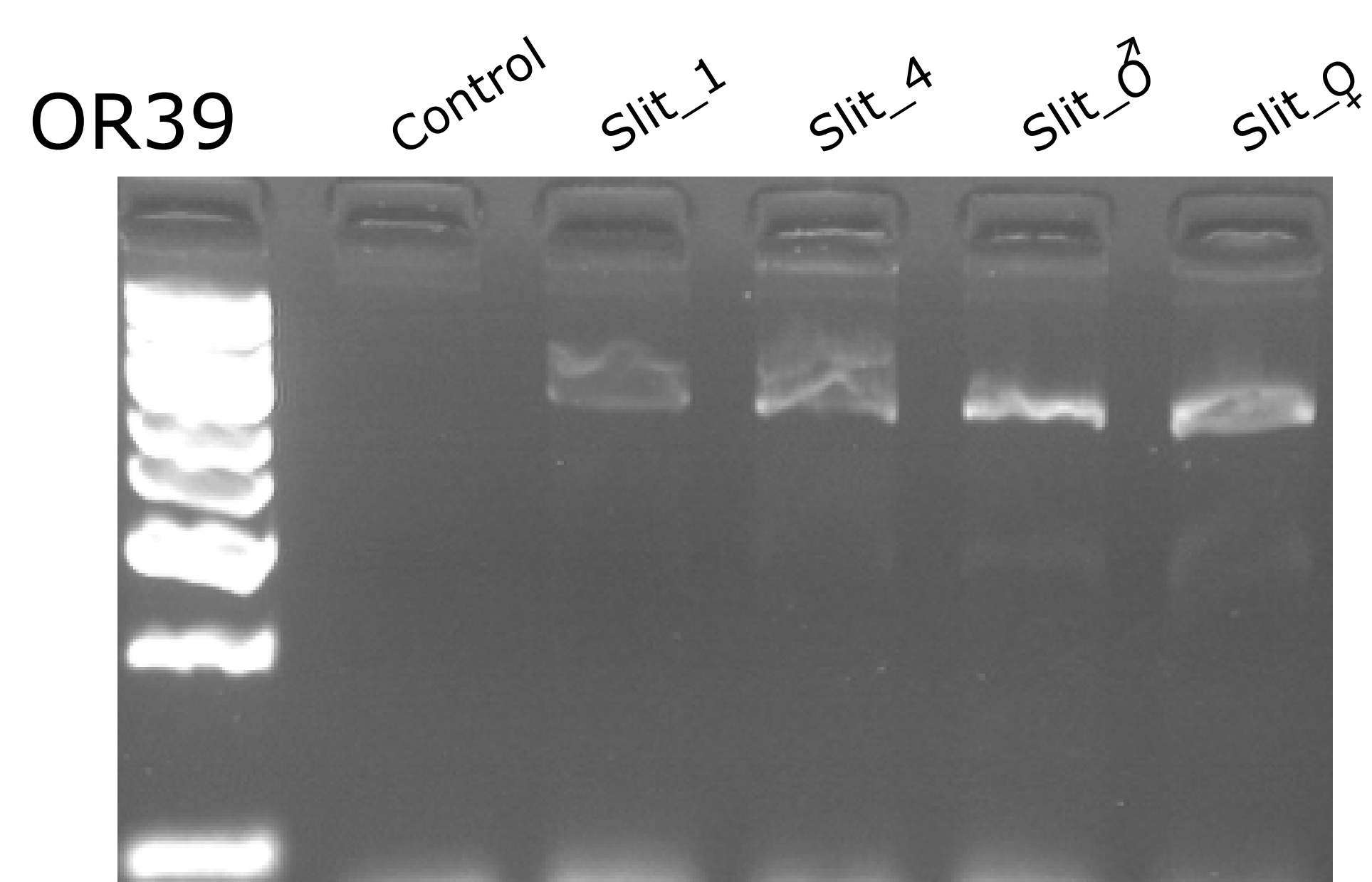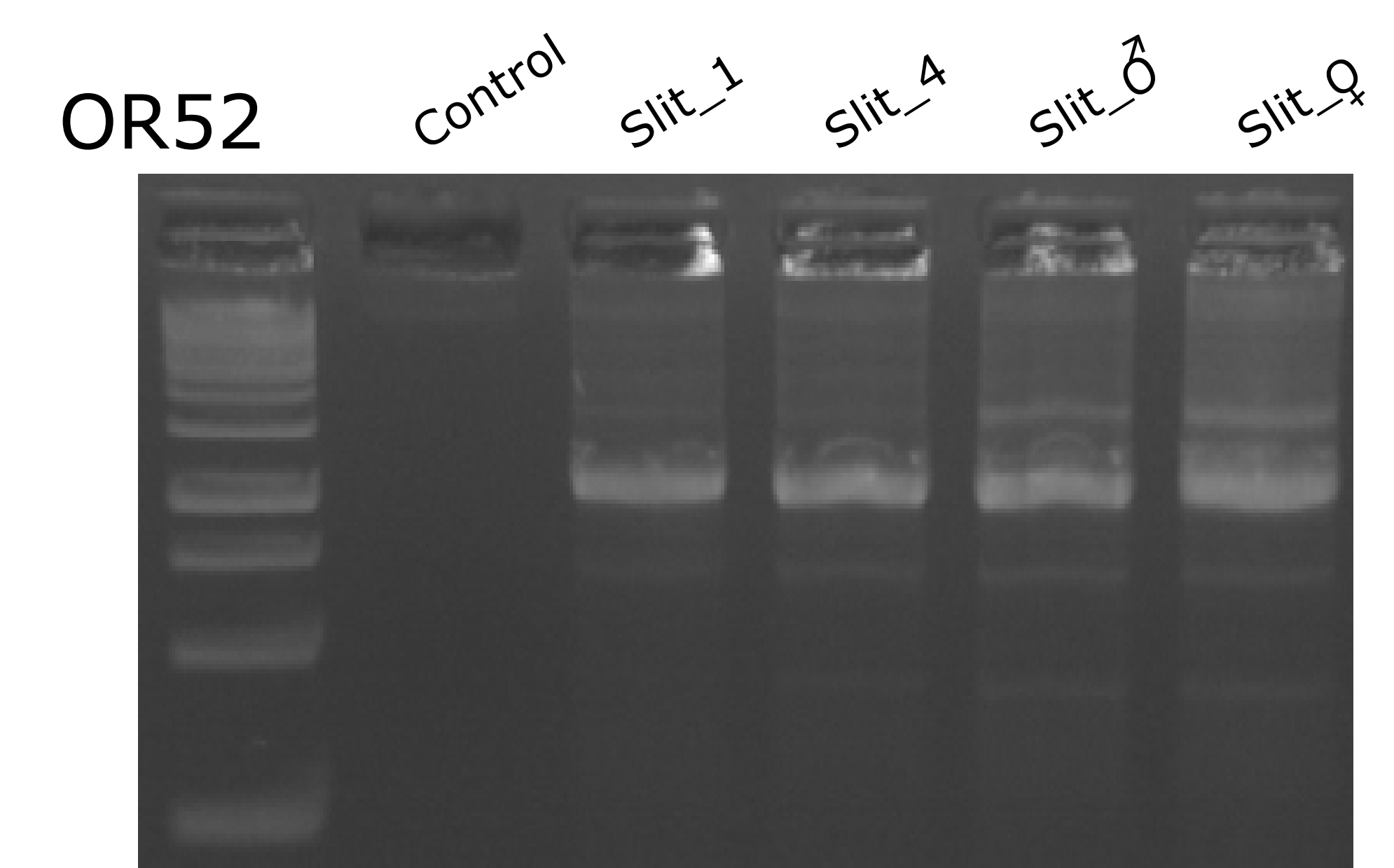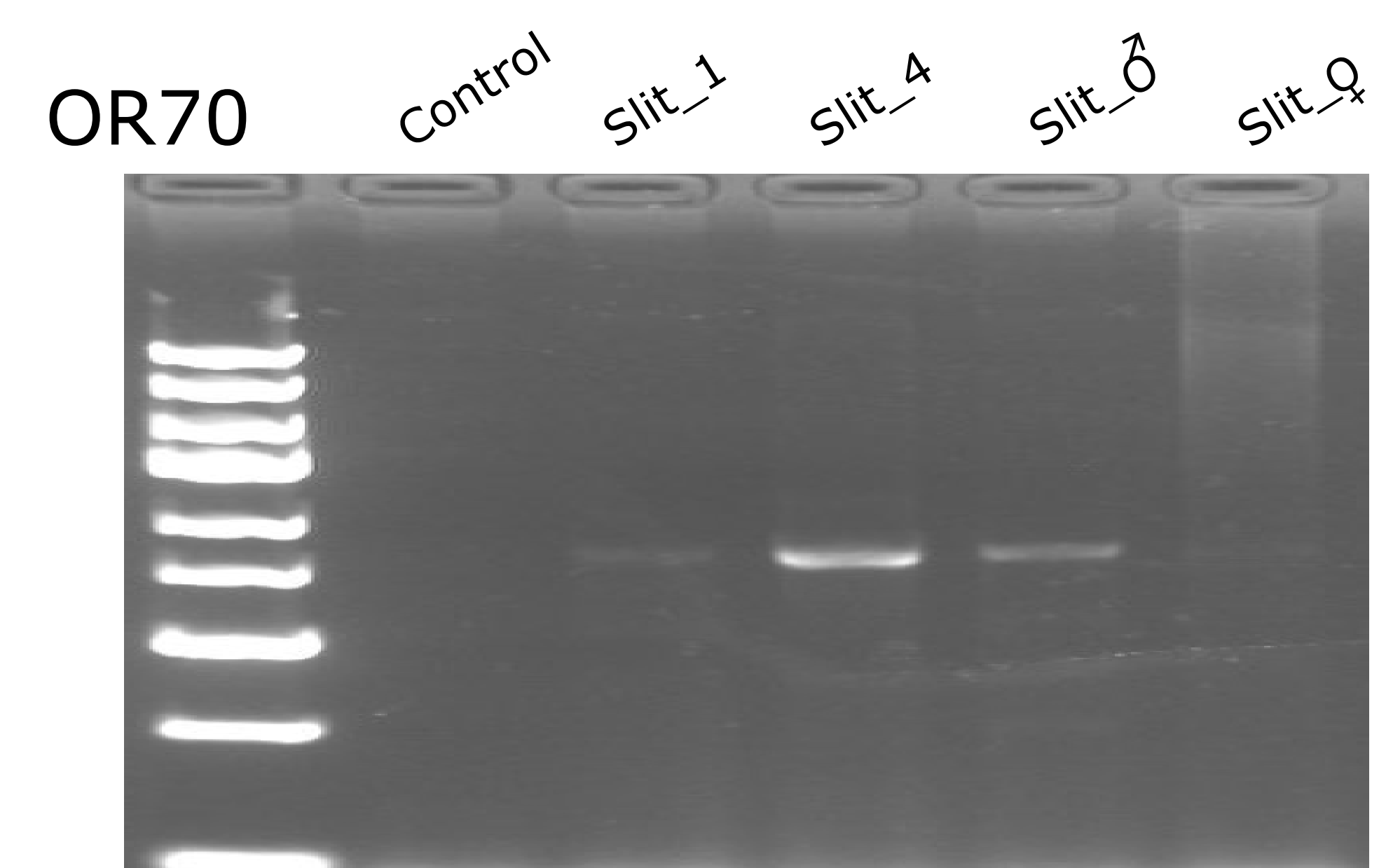

Supplement: Supplementary file 4 — Additional file 4: Figure S1. RT-PCR gel images of PCR-amplified ORs at different developmental stages of Spodoptera littoralis. Comparison included negative-control (control), first instar heads (Slit_1), fourth instar heads (Slit_4), male antennae (Slit_♂) and female antennae (Slit_♀) with 1kb gene ruler ladder (Thermo Fischer Scientific). Red rectangles around SlitOR4 and SlitOR40 represent differential expression in the first and fourth instar, respectively, supported by RT-PCR and transcriptomic analysis. [file 12915_2021_1159_MOESM4_ESM.pdf]

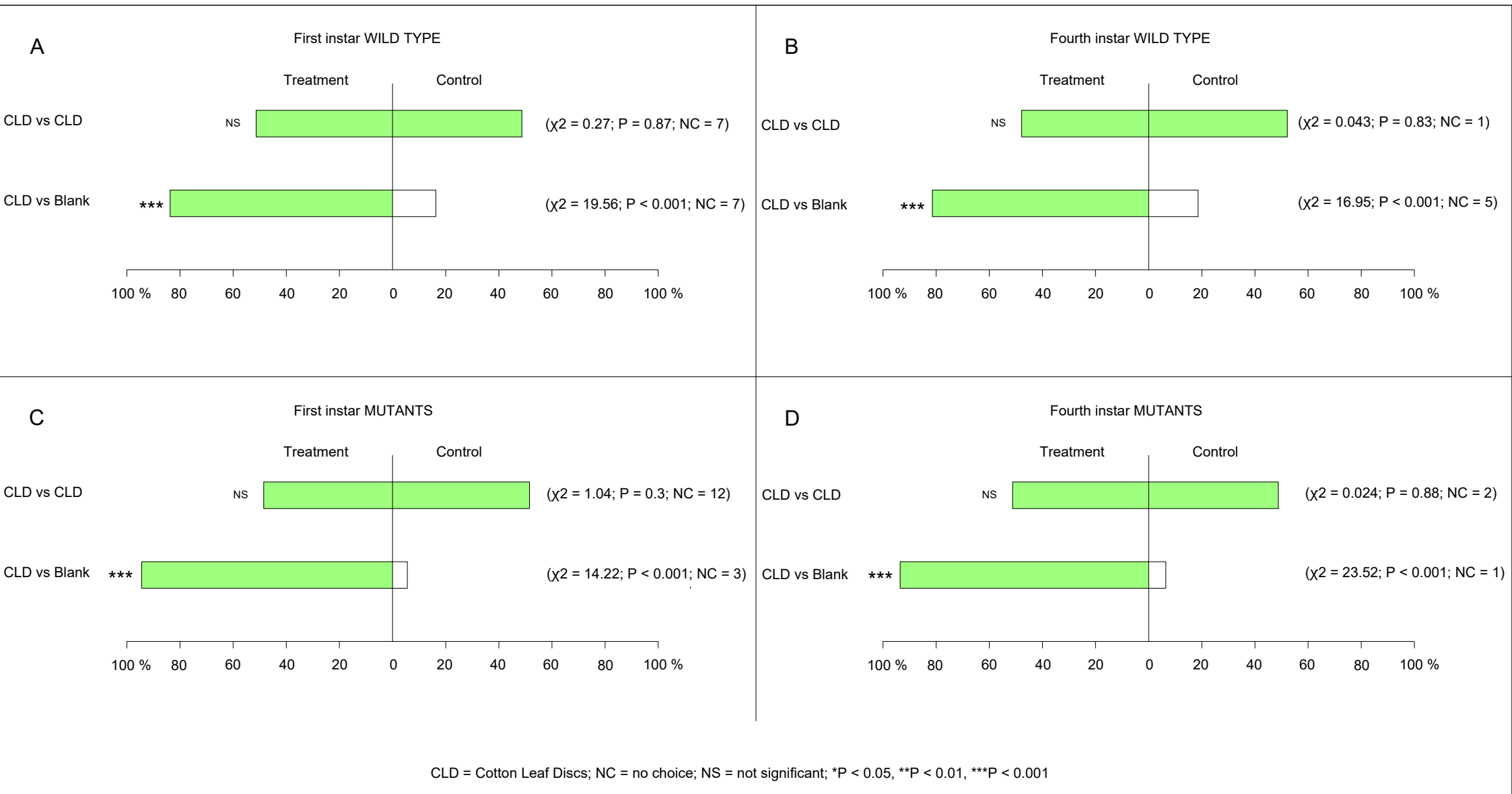

Supplement: Supplementary file 7 — Additional file 7: Figure S4. Attraction of Spodoptera littoralis wild type and SlitOR40-knockout larvae to positive control odors: Attraction of first and fourth instar WT and KO genotypes to positive control odors (CLD, cotton leaf discs) in both arms of the olfactometer (first instar WT: n = 44; fourth instar WT: n = 24; first instar KO: n = 59; fourth instar KO: n = 43) (top bar in Fig. S4A, S4B, S4C and S4D, respectively). Similarly, behavioral responses of WT and KO genotypes of S. littoralis first and fourth instar to the odor of cotton leaf discs versus clean air (control blank) (first instar WT: n = 50; fourth instar WT: n = 48; first instar KO: n = 21; fourth instar KO: n = 32) (bottom bar in Fig. S4A, S4B, S4C and S4D, respectively). [file 12915_2021_1159_MOESM7_ESM.pdf]

# Guaiacol

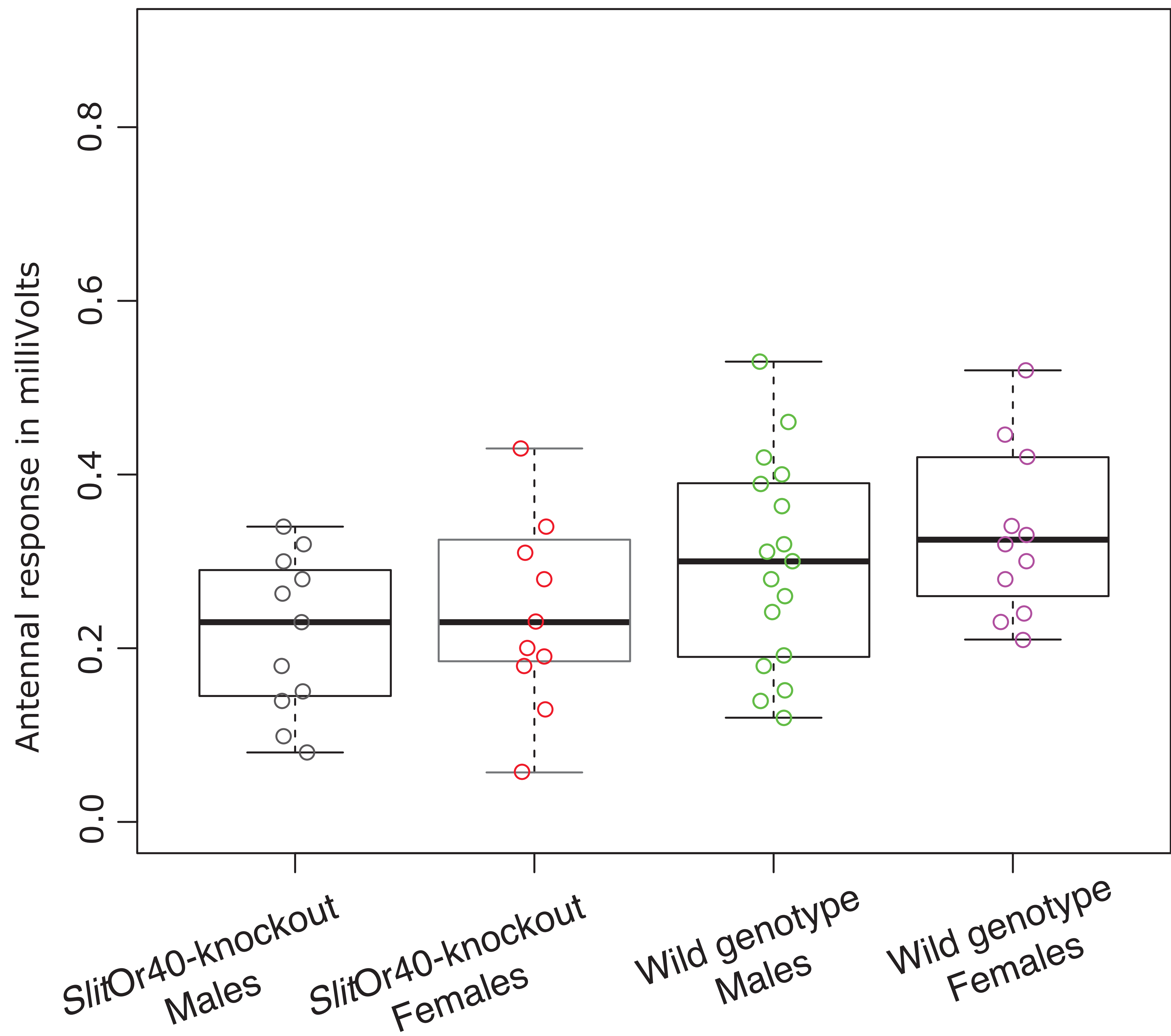

# (±)-linalool

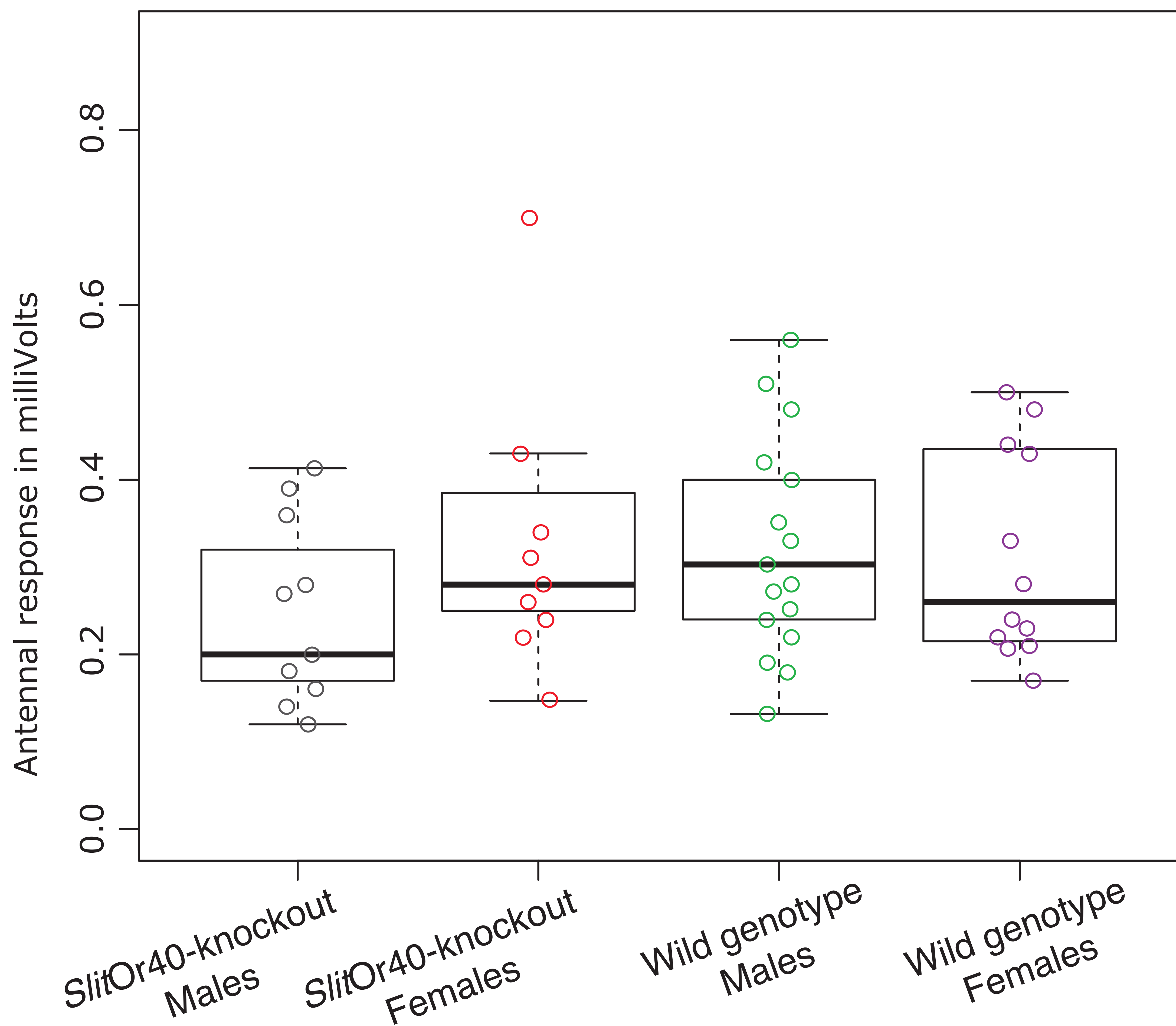

Supplement: Supplementary file 8 — Additional file 8: Figure S5. EAG responses of Spodoptera littoralis wild type and SlitOr40-knockout moth antennae to positive control odors: The EAG responses of WT and KO line adult moth antennae to positive control stimuli (guaiacol and (±)-linalool) were tested at 10 μg dose (minimum n = 9 per sex). There was no statistical difference in responses to positive control stimuli, guaiacol (ANOVA: Sex: Df = 1; F = 1.63; P = 0.2; Genotype: Df = 1; F = 3.55; P = 0.066; Interaction: Df = 1; F = 0.13; P = 0.72; Residual Df = 47) and (±)-linalool (ANOVA: Sex: Df = 1; F = 0.66; P = 0.42; Genotype: Df = 1; F = 0.812; P = 0.372; Interaction: Df = 1; F = 1.68; P = 0.202; Residual Df = 47) in the antennae from both genotypes. The boxplots represent mean values along with the minimum and maximum values. [file 12915_2021_1159_MOESM8_ESM.pdf]

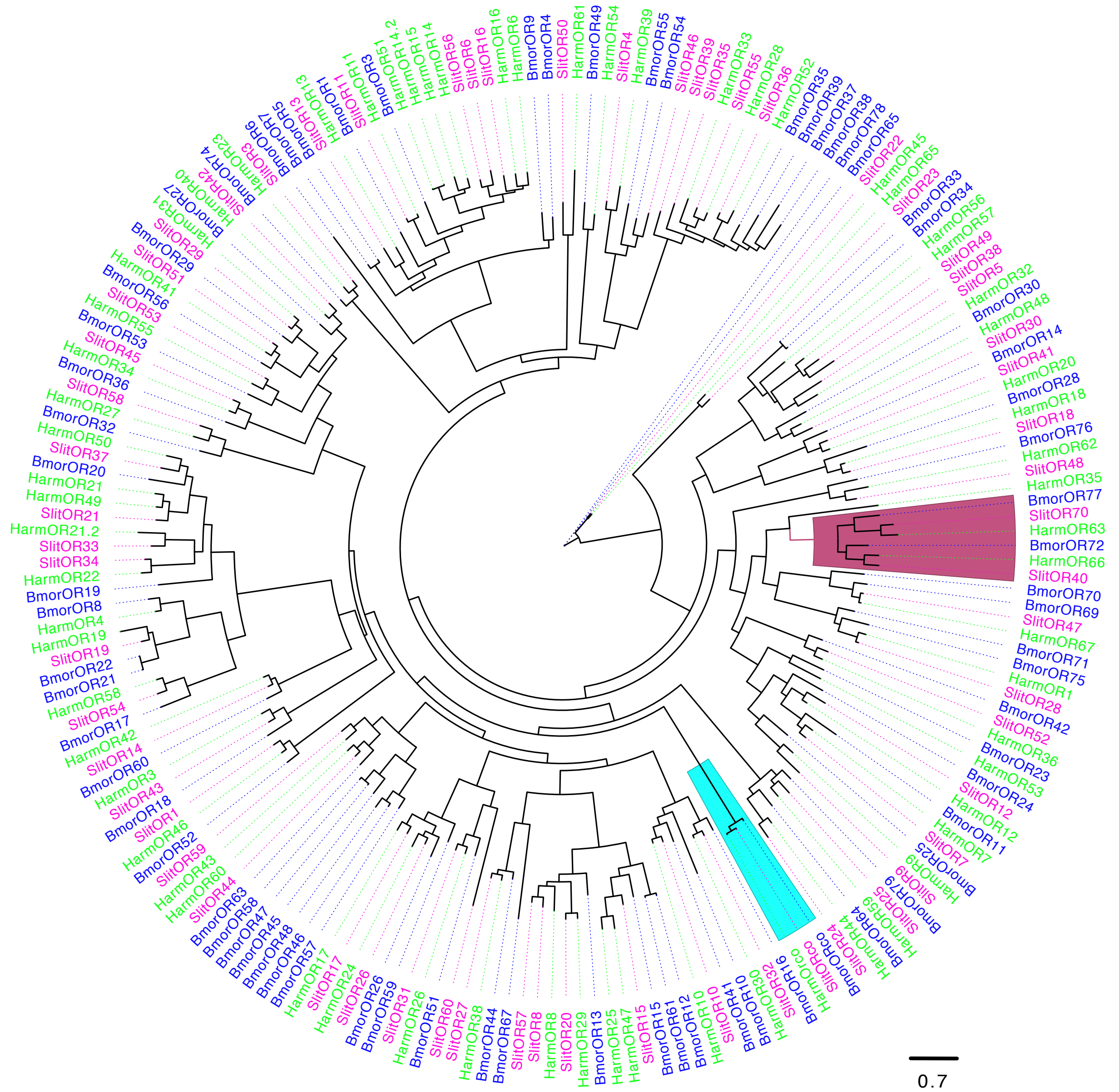

0.7

Supplement: Supplementary file 9 — Additional file 9: Figure S6. Maximum-likelihood phylogenetic tree of ORs expressed in larvae and adults of Spodoptera littoralis: The analyses included all described ORs from S. littoralis (purple), B. mori (blue) and H. armigera (green). Highlighted clades: Orco (cadet blue); SlitOR40 and SlitOR70 along with ORs from B. mori and H. armigera (lilac). The unrooted phylogenetic tree was built using online tool PhyML 3.0. [file 12915_2021_1159_MOESM9_ESM.pdf]
