# Supplementary material for: Stage-specific expression of an odorant receptor underlies olfactory behavioral plasticity in Spodoptera littoralis larvae
Source: BMC Biol. 2021 Oct 28;19:231. doi: 10.1186/s12915-021-01159-1 (PMC8555055; doi:10.1186/s12915-021-01159-1)
Supplement: Supplementary file 5 — Additional file 5: Figure S2. Expression pattern (RT-PCR) and relative expression levels (RT-qPCR) of Orco and SlitOR40 across different instars. (A) RT-PCR products obtained from SlitOrco and SlitOR40 amplification from the cDNA of first, second, third and fourth instar larval heads and adult antennae. (B) Quantitative RT-qPCR expression levels (relative fold-change) of Orco in first instar larval heads, fourth instar larval heads and adult male antennae using β-actin, L13A and Ef1A as reference genes. Male antennae samples expressed significantly higher levels of Orco compared to samples from first and fourth instar larvae (Df = 28, t= -22.622, P<0.001; Df = 28, t= 50.441, P < 0.001, respectively). Similarly, in the fourth instar larvae, Orco expression was significantly higher compared to first instar (Df = 28, t = 9.33, P < 0.001). (C) SlitOR40 expression (using Orco as reference gene) in the first instar larval heads, fourth instar larval heads and adult male antennae. Relative expression of SlitOR40 in first instar larvae was significantly higher compared to adult male antennae (Df = 28, t = 26.532, P < 0.001), while in the fourth larval instar, SlitOR40 expression was not detected (Df = 28, t = 27.693, P < 0.001). The analyses are based on five biological samples. The bars indicate standard errors. Statistical differences between treatments were calculated using a non-paired Student’s t-test. *: P < 0.05. [file 12915_2021_1159_MOESM5_ESM.pdf]

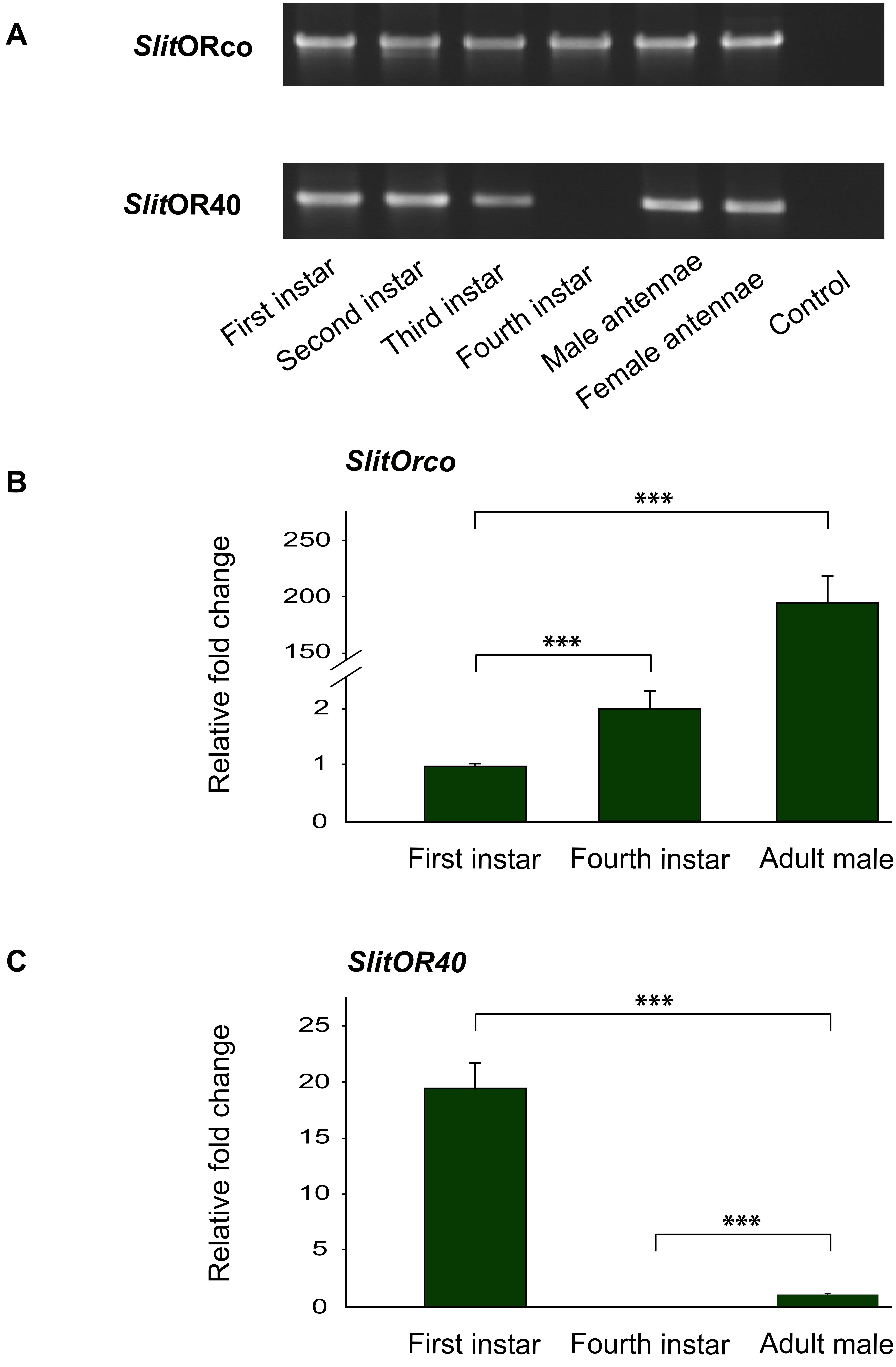

A
SlitORco
SlitOR40
First instar
Second instar
Third instar
Fourth instar
Male antennae
Female antennae
Control
B
SlitOrco
***
***
Relative fold change
250
200
150
2
1
0
First instar
Fourth instar
Adult male
C
SlitOR40
***
***
Relative fold change
25
20
15
10
5
0
First instar
Fourth instar
Adult male
