# Supplementary material for: Stage-specific expression of an odorant receptor underlies olfactory behavioral plasticity in Spodoptera littoralis larvae
Source: BMC Biol. 2021 Oct 28;19:231. doi: 10.1186/s12915-021-01159-1 (PMC8555055; doi:10.1186/s12915-021-01159-1)
Supplement: Supplementary file 6 — Additional file 6: Figure S3. SlitOR40 CRISPR/Cas9 induced indels (insertion and deletion). (A) Wild Type (WT) cDNA sequence including exon1 and exon2. An asterisk separates the two exons’ sequences. Blue letters indicate the primer sequences used for genotyping. Orange and purple boxes surround sequence regions where the Cas9 cleaved SlitOR40 and correspond to the same color boxes in B. (B) The three most prominent double mutational events obtained are shown around each of the two CRISPR guides. Indels’ produced between the two guides are described (NA means not applied) and insertion sequences are indicated in red letters. The number of nucleotide differences (Δ) between the WT line and the three mutant obtained lines are given between brackets. In both A and B, green letters show the CRISPR guide sequences with their PAM highlighted in yellow. (C) Electrophoregram traces for each of the three main double mutations obtained (BioEdit). Line 10 mutants were chosen for further crossing and phenotypic analysis. [file 12915_2021_1159_MOESM6_ESM.pdf]

A

Line WT, cDNA 601 bp exon1 * exon2:

ATGGAAGAGCTGCCTGAAATTTCAAAAGAATTCATCGAACCGATATTACCATGTCTGGGTTTATTGAGAAATGCCCATATTTTGATTTATGCACATGGAAAACCTTTTTGG
AATGAAAAAATGTCATTTGACCTTAAACATAGTTTCAGTTGTAACTTATCTATTTTCTTTGACTATGTATATGGGAAAAGTATTTAGAGGAGAATTACAACTTGAGGAGTTGGCTT
ATGTTATCAGCGTTTATGTCGTCAGTGTACAAG * GTAAGCTAATTTTTTTTAAACTTTATTTCTTAAAATAGTGTTTATTGTTGAACAAACCATAACGGATTTTATTCTATTTGAC
AAAGCCAAAAATATTACTCTATCCTCTGCATTACTATTCAAACATCACGTCGACCTCGTTCTCACGTCACAAAACTCTCAAATATACCAGAACATCCCAATTTCCCGTACATTTT
TCCTGCATATAATATTTTCTCTAAATTCAAAGAACAAAAGACGTTTTCGCCAACGTTTTATATCAACACCTTGCATACGGAAACATGCTGTATACTCTTAACTGAACTCTGAATT
TCTCAGCCATCCTCAAAGGTTCGGTTGTCGTCCTTTACAAGAAGGAAATAAGGGCTATAATCCTGCAGCTGGGAGGTATGTGGAGGATGGAACATCTAACTGAAGTACAGA
TTAACAAGAAGAATACGTTACTGAGGAGACTAAAGTTCTGTTATGCAGTTTTCTACTGGATTAATATGATTGGATCCTGGCAATACATACTGGCTCCTTTATTGGAAACAGTTA
CCCGCAAATTTATTTTACTGCAGGAATGTGGGCTGTTACTTCCATTTTCTTGCAGCTTTCCTTTTGATCCTACTGGGAACTGGGCAAGATATATCGGTGTATATATTTTTGAGA
CTTACAGCA

B

	exon1 mutation	coding region indels	exon2 mutation
Line WT, Δ (NA):	GGGAAAAGTATTTAGAGGAGAATTACAACTTGAGGAGTTGGCTT	NA	GGGCTATAATCCTGCAGCTGGGAGGTATGTGGAGGATGGAACATCTAA
Line 38, Δ (420):	GGGAAAAGTATTTAGAGGAGAATTACAA:::::::::::::::::::::::::::::::	- 440 bp + 20 bp	::::::::::::::::::::::::GGATTATTTGGAGAATTATTTGGAGGATGGAACATCTAA
Line 10, Δ (445):	GGGAAAAGTATTTAGAGGAGAATTAC::::::::::::::::::::::::::::::::::	- 445 bp	:::AGGATGGAACATCTAA
Line 2, Δ(441):	GGGAAAAGTATTTAGAGGAGAA::	- 446 bp + 5 bp	::GTATTTGGAGGATGGAACATCTAA

C

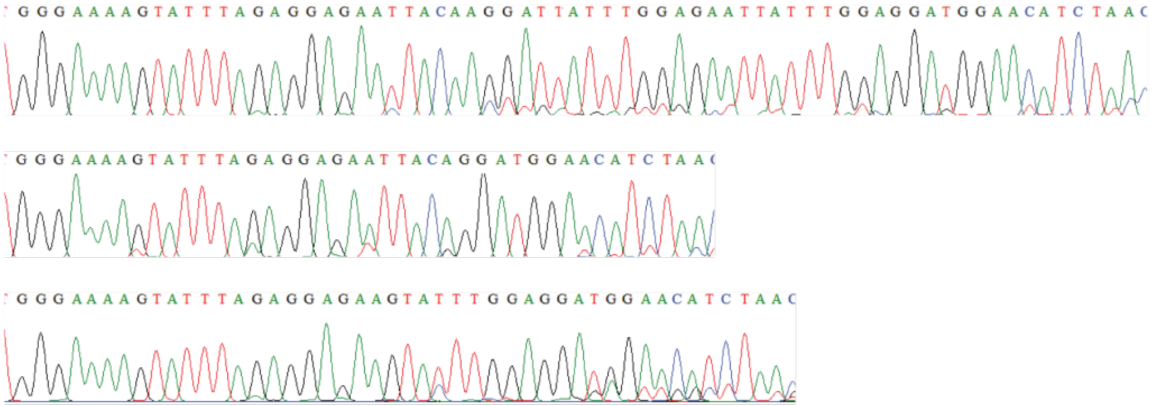

Line 38, Δ(420):

Line 10, Δ(445):

Line 2, Δ(441):
