# Supplementary material for: Stage-specific expression of an odorant receptor underlies olfactory behavioral plasticity in Spodoptera littoralis larvae
Source: BMC Biol. 2021 Oct 28;19:231. doi: 10.1186/s12915-021-01159-1 (PMC8555055; doi:10.1186/s12915-021-01159-1)
Supplement: Supplementary file 10 — Additional file 10: Table S4. Primers used in the study: List of all primers used to confirm the expression of SlitORs, qRT-PCR assays in first and fourth larval instars, and primers used for genotyping. [file 12915_2021_1159_MOESM10_ESM.pdf]

Annotation	Forward primer	Temperature	Reverse primer	Temperature
ORCo	TACGACGGCTCCGAGGAA	58.2	TTACTTCAGCTGTACCAACACCAT	59.3
SlitOR1	ATGGACTCGAATGTAGATAAGA	54.7	TTACTTTTCATTGACATTAACTAGAAGAG	58.2
SlitOR3	ATGGATGAAGCTTTTCTCGCG	57.9	AAACGGACATCACGAGTGTAAAGA	59.3
SlitOR4		57.9	ATAGACTGCATAGTGCTCGCG	59.8
SlitOR5	TGACGAACCAAAAGGACTTGG	57.9	TGTTGAAACAAGTCAGTTAGGGCA	59.3
SlitOR6	ATGGGTTTAAAAAAGTTTCTTTTTG	57.9	TCAAATGCTGCGTAGGAAGGT	53.1
SlitOR7	ATGCCCAAACCACTGTTATTTGAC	59.3	TTATCTTCCGTACACAGTCTGCAG	61
SlitOR8	CTGATGCTCTGCCACTTCTAGTT	60.6	GCAAAATGATTTGGTTTCCATAA	53.5
SlitOR9	AGATTATCGTGGTGATAGCGAGAC	61	CGAAGTACGACCAGGCTGTAC	61.8
SlitOR10	ATGGAGGCAGAAAAAAACACG	55.9	TTATTCTGTGTTCACCCTGTTCA	57.1
SlitOR11	TCGAGACCAACTTCAAGGGC	59.4	GTACATCTCGCTGCCGATCA	59.4
SlitOR12	ATGGAAGAAGAACCTCTGTTAA	54.7	TTATGGAGGCGAACCATACAA	55.9
SlitOR13	ATCATGTGGGTGATCGACGG	59.4	AGTTGATCACCAGGCTGTGG	59.4
SlitOR14	TAGGTCTGGGATGGTGGC	58.2	TAAGCTGCCTTCATGATAGCTAGA	59.3
SlitOR15	CATTTGGCCCGGACTCAA	56	TAGAGGAAACATCTTCAACCCTGA	59.3
SlitOR16	ATGTACATAAAAATAGTGCGGTTCA	56.4	TCACATGCTTCGTAAGAAGG	55.3
SlitOR17	ATGTCGGTGTGTGCCGG	57.6	TTATTTAGTTTGCCGTAGAACTGCA	58.1
SlitOR18	ATGGAAATGAAATCAGATATTCTGA	54.8	TCAAGCNGTAATCAAAGTGAAGA	56.2
SlitOR20	AAATAACATTTGCGGTGTACGACT	57.6	CGCCGTAAAAGTAGCAAGTGATAG	61
SlitOR19	AAGAATTTTCTTAATCGGCTCCGG	59.3	CAGTTGATCCGAGAGGGCAAT	59.8
SlitOR21	CCACGACAGGTCTGTGCTTA	59.4	TCTTGAACTGATGGCTGACG	57.3
SlitOR22	GAAGGCATCCTTTGATTTCGACAG	61	GAATATGAAGAGCTCATGCACGAG	61
SlitOR23	CTGCACCACCAGAGCATGTA	59.4	CAGAAGTAGCAGGGCACGAA	59.4
SlitOR24	ATGAGGGTCTTAAGCCATGTGT	58.4	TCATTCGTGGCTCATCGTTAGA	58.4
SlitOR25	AGCTTTCTGTTCCTGGCGTA	57.3	ATGATGGTAGACCGCACTCC	59.4
SlitOR26	ATGTCTCTGTCGTCGGGC	58.2	GGCGCAGTACTGCGTAGA	58.2
SlitOR27	GAAAACCAAATTGGCGTTTCTGTC	59.3	TCCGCATTACGGCAAAGG	56
SlitOR28	ATGACGTCTCTTTATAGAAAATTTTT[illegible]	58.6	TTAATTAACACTTTTCTCTTTGAAGAAGTT	57.2
SlitOR29	GAGCTTAGAAGATCCAGCGCG	61.8	TGAAGGTCGGCAGAGTAAGGT	59.8
SlitOR30	CGATTAAACCGATCACATCCGTCT	61	AGCTCAGGATCGCGTAGGATT	59.8
SlitOR31	ATGGAAGATAATGTAGCATACTCTAC[illegible]	61	CTAGCGATTCAAGAACGTAAACAATGTG	62.2
SlitOR32	ATGGTCTCCTCAGAAGACCTTTTT	59.3	GCAACAAAAGTTTGTCGCGAC	57.9
SlitOR33	CACCATGTTCATCACCAGCG	59.4	CAGGGTCTTGTACACCACGG	61.4
SlitOR34	TTCCTATGCTGCTGTCCAGATTTC	61	CGAGAATGTCAAGTGTCCAAAGGC	62.7
SlitOR35	TGGTTCGTGTTCGTGAGGAG	59.4	CGATCTCGTAGTTGGGGCTC	61.4
SlitOR36	AGTTTGGTTTGGAGTACTGTGACC	61	GGAATAAATGCCTCTGAAGACGGA	61
SlitOR37	AAGCATTTCGGTAATACCTGTGCT	59.3	AAGCATTTCGGTAATACCTGTGCT	59.3
SlitOR38	ATGGCTGACCAATTCGAGAAAACT	59.3	AGATAGTGTAGGCCTGCTGAAACA	61
SlitOR39	GGTTTGGAGTATTGTGAGCTACCG	62.7	GCTGTCAGGGTCATAGTCTTCTGT	62.7
SlitOR40	TGCAGGAATGGCAGATTCAGT	57.9	CGACCAAGTTGTGCTCAGTAC	59.8

<table>
<tr><th>Annotation</th><th>Forward primer</th><th>Temperature</th><th>Reverse primer</th><th>Temperature</th></tr>
<tr><td>SlitOR41</td><td>ACCACCTTCCACGAGACCTA</td><td>59.4</td><td>CAGGTCCACGAACTGGAACA</td><td>59.4</td></tr>
<tr><td>SlitOR42</td><td>GCCAAGAAGGACGACACCAT</td><td>59.4</td><td>TGCTCAGGTAGAACACGTCG</td><td>59.4</td></tr>
<tr><td>SlitOR43</td><td>ATGGTGCTCTCCGGACTAGAA</td><td>59.8</td><td>TCGTTGAGAACTGCGAAGATGGAA</td><td>61</td></tr>
<tr><td>SlitOR44</td><td>GCGACCCAATCAGTCAGC</td><td>58.2</td><td>TACGAAGCTTTGATGATTGCCATG</td><td>59.3</td></tr>
<tr><td>SlitOR45</td><td>ATGTCGGACAAATGTTCGATTGGA</td><td>59.3</td><td>CTGGCCCGTAAGATCGCAATA</td><td>59.8</td></tr>
<tr><td>SlitOR46</td><td>TGGCAACAATAGTGTGTGGTTTGT</td><td>59.3</td><td>GGTGGAACTTCTGGCAGC</td><td>58.2</td></tr>
<tr><td>SlitOR47</td><td>ACAATAGAACAAGCGAAACGGGAA</td><td>59.3</td><td>GAAGTACGAGTACGACCTCGT</td><td>59.8</td></tr>
<tr><td>SlitOR48</td><td>ACAACACCGCCTACATCTGG</td><td>59.4</td><td>TCAGCAGCATCCTCTTGGTG</td><td>59.4</td></tr>
<tr><td>SlitOR49</td><td>GAAGGGCGAGTTCTTCGTGT</td><td>59.4</td><td>GTGGCCTCGTAGAACACCTT</td><td>59.4</td></tr>
<tr><td>SlitOR50</td><td>CACCGTGTTCCTGTTCGAGA</td><td>59.4</td><td>TGATGCTGGGCCACTTGTAG</td><td>59.4</td></tr>
<tr><td>SlitOR51</td><td>GATGTTCAAAAATATTCTCCAGAAATTGG</td><td>59.6</td><td>TTCATTTGAGACTGACTCAGCAGC</td><td>61</td></tr>
<tr><td>SlitOR52</td><td>TATGGAAAATATTCCCCCATACAAAGATT</td><td>59.6</td><td>TTTGCACAAACGTGGGAAGTCAGA</td><td>61</td></tr>
<tr><td>SlitOR53</td><td>GGACAGGATCAAGGAGTGCC</td><td>61.4</td><td>TGTACTGGAACACCCACAGC</td><td>59.4</td></tr>
<tr><td>SlitOR54</td><td>ATGGGACCTCTATACAGTATCCAGGTG</td><td>65</td><td>TGCCACTACAGCCAAGGTTCG</td><td>61.8</td></tr>
<tr><td>SlitOR55</td><td>ATGATGGAGACGCTCCGAAGG</td><td>61.8</td><td>GCCAACTCCTAGAATAACAAATTCTAAAG</td><td>61</td></tr>
<tr><td>SlitOR56</td><td>GATGGGTTTAAGAAACTTTCTTTTTGGAAA</td><td>59.9</td><td>AATGTGAAGTAAGACAATGAAGTTCTCA</td><td>59.3</td></tr>
<tr><td>SlitOR57</td><td>GGTGACCGACGAGAACAAGA</td><td>59.4</td><td>CTGGTCCAGTCGCAGTTGTA</td><td>59.4</td></tr>
<tr><td>SlitOR58</td><td>ATGCGAGGGATGGAATGTTTG</td><td>57.9</td><td>TTCTTTCTTTCCCAACACAGCGTA</td><td>59.3</td></tr>
<tr><td>SlitOR59</td><td>GAGTTCTTCAGTGACGATAGCGTC</td><td>62.7</td><td>TGGAATGCCTTCACAGAGCCT</td><td>59.8</td></tr>
<tr><td>SlitOR60</td><td>AAATGTCCGAACAAACAATAACGCCGT</td><td>61.9</td><td>GTTTCGTACTGAGAACCGCAAACA</td><td>61</td></tr>
<tr><td>SlitOR70</td><td>ATGGAGGTTCTTAAAGACTTCCCCGAA</td><td>63.4</td><td>GAGAGAGCCAAGCTCTGATATAAATGT</td><td>61.9</td></tr>
<tr><td>>gi|600074|emb|Z46873.1| Spodoptera littoralis mRNA for partial beta-actin</td><td>GTACGTCGCCATCCAGGC</td><td>60.5</td><td>CGTGGGGCAGAGCGTAAC</td><td>60.5</td></tr>
<tr><td>>gi|288226776|gb|FJ979921.1| Spodoptera littoralis 60S ribosomal protein L13a mRNA, complete cds</td><td>CAAGTCAGTCAAGCTTATGGCCTA</td><td>61</td><td>CATAGGATTGGATCACTGCGG</td><td>59.8</td></tr>
<tr><td>>gi|805487969|gb|KP682697.1| Spodoptera littoralis voucher SPCAM0144 elongation factor 1a gene, partial cds</td><td>GCTGGTGACTCCAAGAACAAC</td><td>59.8</td><td>ACTTGCAGGCAATGTGGG</td><td>56</td></tr>
<tr><td>SlitOR40 (qPCR)</td><td>TGCAGGAATGGCAGATTCAGT</td><td>57.9</td><td>CGACCAAGTTGTGCTCAGTAC</td><td>59.8</td></tr>
<tr><td>SlitORCo (qPCR)</td><td>TACGACGGCTCCGAGGAA</td><td>58.2</td><td>TTACTTCAGCTGTACCAACACCAT</td><td>59.3</td></tr>
<tr><td>SlitOR40 (Genotyping)</td><td>TCGAACCGATATTACCATGTCTG</td><td>58.9</td><td>ACTTTAGTCTCCTCAGTAACGT</td><td>56.5</td></tr>
</table>
