# Supplementary material for: Stage-specific expression of an odorant receptor underlies olfactory behavioral plasticity in Spodoptera littoralis larvae
Source: BMC Biol. 2021 Oct 28;19:231. doi: 10.1186/s12915-021-01159-1 (PMC8555055; doi:10.1186/s12915-021-01159-1)
Supplement: Supplementary file 11 — Additional file 11: Table S5. List of chemical compounds used in the electrophysiological recordings. [file 12915_2021_1159_MOESM11_ESM.pdf]

	Compounds	CAS No.		Compounds	CAS No.
1	Isoamyl acetate	123-92-2	28	Ethyl butyrate	105-54-4
2	Geraniol	106-24-1	29	Ethyl hexanoate	123-66-0
3	1-heptanol	111-70-6	30	Ethyl lactate	687-47-8
4	1-hexanol	111-27-3	31	Ethyl-3-hydroxy butyrate	5405-41-4
5	1-indanone	83-33-0	32	Eugenol	97-53-0
6	1-nonanol	143-08-8	33	Farnesol acetate	4128-17-0
7	1-octanol	111-87-5	34	Geranyl acetate	105-87-3
8	1-octen-3-ol	3391-86-4	35	Guaiacol	90-05-01
9	2-heptanone	110-43-0	36	Hexyl acetate	142-92-7
10	2-pentanol	6032-29-7	37	Alpha-humulene	6753-98-6
11	2-Phenyl ethanol	60-12-08	38	Isobutyl acetate	110-19-0
12	2,3-butendiol	513-85-9	39	Jasmonone	137-03-1
13	3-methyl-2-buten-1-ol	556-82-1	40	Linalool	126-91-0
14	3-octanol	589-98-0	41	m-cresol	108-39-4
15	Acetoin	513-86-0	42	Methyl benzoate	93-58-3
16	Benzaldehyde	100-52-7	43	Methyl hexanoate	106-70-7
17	Benzyl alcohol	100-51-6	44	Methyl salicylate	119-36-8
18	Butyl acetate	123-86-4	45	Myrcene	123-35-3
19	Carvacrol	499-75-2	46	Ocimene	13877-91-3
20	Beta-caryophyllene	87-44-5	47	p-cresol	106-44-5
21	Citronellol	106-22-9	48	Pentyl acetate	628-63-7
22	DMNT	19945-61-0	49	Sulcatone	110-93-0
23	E2-hexenal	6728-26-3	50	Alpha-Terpeniol	10482-56-1
24	E2-hexenol	928-95-0	51	Thymol	89-83-8
25	E3-hexenol	928-97-2	52	Z2-hexanol	928-94-9
26	Estragole	140-67-0	53	Z3-hexanol	928-96-1
27	Ethyl benzoate	93-89-0	54	Z3-hexenyl acetate	3681-71-8
